# Supplementary material for: High-speed 3D DNA PAINT and unsupervised clustering for unlocking 3D DNA origami cryptography
Source: Nat Commun. 2025 Dec 13;16:11514. doi: 10.1038/s41467-025-66338-y (PMC12749235; doi:10.1038/s41467-025-66338-y)
Supplement: Supplementary file 1 — Supplementary Information [file 41467_2025_66338_MOESM1_ESM.pdf]

Supplementary Information

# High-speed 3D DNA PAINT and unsupervised clustering for unlocking 3D DNA origami cryptography

**Gde Bimananda Mahardika Wisna** [a,b,✉], **Daria Sukhareva**[b,c], **Jonathan Zhao**[c,d], **Prathamesh Chopade** [b], **Deeksha Satyabola** [b,c], **Michael Matthies** [b], **Subhajit Roy**[a,b], **Chao Wang** [b,e], **Petr Šulc** [b,c], **Hao Yan**[b,c], **and Rizal F. Hariadi** [a,b,✉]

[a]Department of Physics, Arizona State University, Tempe, Arizona, USA.; [b]Center for Molecular Design and Biomimetics at the Biodesign Institute, Arizona State University, Tempe, Arizona, USA.; [c]School of Molecular Sciences, Arizona State University, Tempe, Arizona, USA.; [d]School of Computing and Augmented Intelligence, Arizona State University, Tempe, Arizona, USA.; [e]School of Electrical, Computer and Energy Engineering, Arizona State University, Tempe, Arizona, USA.

✉ E-mail: gwisna@asu.edu ,rhariadi@asu.edu

## Contents

**Supplementary Methods** **2**
Unsupervised classification . . . . . 2
ResNet CNN supervised classification. . . . . 2

**Supplementary Tables** **3**
Supplementary Table 1. 2D RRO strands . . . . . 3
Supplementary Table 2. 3D wireframe cuboctahedron DNA origami strands . . . . . 8
Supplementary Table 3. Letters to binary and numbers to binary. . . . . 16
Supplementary Table 4. Mixing concentrations for all experiments in the figures in the main text. 16
Supplementary Table 5. DNA-PAINT super-resolution imaging parameters for each experiment. 16
Supplementary Table 6. DNA origami tunnel strands . . . . . 18
Supplementary Table 7. Tetrapod strands . . . . . 22
Supplementary Table 8. Tetrapod Pattern Mixing. . . . . . 27
Supplementary Table 9. Picasso Localize module parameters. . . . . . 29
Supplementary Table 10. Parameter selection. . . . . . 29
Supplementary Table 11. Benchmarking of nanoscale readout modalities for DNA-based data storage . . . . . 30

**Supplementary Figures** **31**
Supplementary Fig. 1: Additional AFM images of 2D RRO and 3D wireframe cuboctahedron DNA origami. . . . . . 31
Supplementary Fig. 2: 2D RRO cadnano design showing scaffold routing and staple strands interlacing. . . . . . 32
Supplementary Fig. 3: Detection efficiency analysis procedure in 20 nm RRO with 32nt binder. . 33
Supplementary Fig. 4: Theoretical design of 10 nm resolution encryption pattern resulting in 228 combinations of numbers, letters, and punctuation marks forming texts assuming 100% incorporation efficiency. . . . . . 34
Supplementary Fig. 5: All picks in the analyzed NSF dataset. . . . . . 35
Supplementary Fig. 6: All picks in the analyzed ASU one redundancy dataset. . . . . . 36

Supplementary Fig. 7: ASU two redundancy dataset on higher density 2D RRO along with All analyzed picks. . . . . 37
Supplementary Fig. 8: All analyzed picks in ASU two redundancy dataset on higher density 2D RRO. . . . . 38
Supplementary Fig. 9: The argument regarding the readout accuracy increases as more bits are used by a pattern. . . . . 39
Supplementary Fig. 10: Schematics of confused patterns due to 2D projections from 3D DNA origami encryption design. . . . . 40
Supplementary Fig. 11: 3D wireframe cuboctahedron DNA origami design. . . . . 41
Supplementary Fig. 12: 2D view of all picks analyzed in 3D DNA-PAINT 0407 dataset. . . . . 42
Supplementary Fig. 13: 3D clustering and alignment of DNA-PAINT experimental data and 3D cuboctahedron DNA origami structure. . . . . 43
Supplementary Fig. 14: 3D tunnel DNA origami. . . . . 43
Supplementary Fig. 15: 2D view of all picks analyzed in 3D DNA-PAINT 1776 dataset. . . . . 44
Supplementary Fig. 16: Design of two color 2D RRO encryption for high density information. . . 45
Supplementary Fig. 17: Simulated DNA-PAINT using the Picasso Simulate module . . . . . 47
Supplementary Fig. 18: Incomplete staple incorporation prevents encrypted pattern formation. . 48
Supplementary Fig. 19: Unsupervised classification result on NSF dataset. . . . . 49
Supplementary Fig. 20: ResNet CNN results on synthetic data generated by Picasso Simulate module of letters A-Z without position encoding. . . . . 50
Supplementary Fig. 21: 3D calibration curve generated by the Localize feature of Picasso. . . . . 51

## Supplementary Methods

**Unsupervised classification.** We run unsupervised classification[1] to classify a pre-labeled mixture of NSF and ASU datasets into several classes. The super particles of each class are then fed into our template alignment method to read out the bit. We counted the number of each label in each class to calculate the accuracy (Supplementary Fig. 19).

**ResNet CNN supervised classification[2].** Picasso's Simulate and Render modules were utilized to generate 75x75 images of each of the 26 letters from the alphabet. Images were then filtered using the root mean squared distance between each pixel and the center of mass of the image, for a total of 41,096 images. The dataset was split into a training dataset of 22,749 images and testing dataset of 18,347 images. Twenty percent of the training set was further split into a validation dataset that was used to select the final model. Our ResNet implementation used transfer learning on ResNet-50. The final layer was replaced with a linear layer that gives 12 outputs with a sigmoid activation, one for each binding site in the encryption template of the NSF pattern. A threshold of 0.5 was used to distinguish between bits that are on and off, which are then decoded into the corresponding letter (Supplementary Fig. 20). The network was trained using the Pytorch implementation of the Adam optimizer at a learning rate of $10^{-3}$ for the linear layer and $10^{-4}$ for the other layers for 20 epochs. A cosine annealing learning rate scheduler and binary cross-entropy loss function were used. After training, the epoch with the lowest loss over the validation set was selected as the final model. The model then ran the predictions over the testing dataset.

## Supplementary Tables

| Name | Sequence | Note |
|---|---|---|
| 0[47]1[31] | AGAAAGGAACAACTAAAGGAATTCAAAAAAA | Core staple |
| 1[96]3[95] | AAACAGCTTTTTGCGGGATCGTCAACACTAAA | Core staple |
| 2[111]0[112] | AAGGCCGCTGATACCGATAGTTGCGACGTTAG | Core staple |
| 3[160]4[144] | TTGACAGGCCACCACCAGAGCCGCGATTTGTA | Core staple |
| 5[160]6[144] | GCAAGGCCTCACCAGTAGCACCATGGGCTTGA | Core staple |
| 6[239]4[240] | GAAATTATTGCCTTTAGCGTCAGACCGGAACC | Core staple |
| 7[224]9[223] | AACGCAAAGATAGCCGAACAAACCCTGAAC | Core staple |
| 8[239]6[240] | AAGTAAGCAGACACCACGGAATAATATTGACG | Core staple |
| 9[224]11[223] | AAAGTCACAAAATAAACAGCCAGCGTTTTA | Core staple |
| 10[239]8[240] | GCCAGTTAGAGGGTAATTGAGCGCTTTAAGAA | Core staple |
| 11[224]13[223] | GCGAACCTCCAAGAACGGGTATGACAATAA | Core staple |
| 13[96]15[95] | TAGGTAAACTATTTTTGAGAGATCAAACGTTA | Core staple |
| 0[79]1[63] | ACAACTTTCAACAGTTTCAGCGGATGTATCGG | Core staple |
| 1[128]4[128] | TGACAACTCGCTGAGGCTTGCATTATACCAAGCGCGATGATAAA | Core staple |
| 2[143]1[159] | ATATTCGGAACCATCGCCCACGCAGAGAAGGA | Core staple |
| 3[224]5[223] | TTAAAGCCAGAGCCGCCACCCTCGACAGAA | Core staple |
| 5[224]7[223] | TCAAGTTTCATTAAAGGTGAATATAAAAGA | Core staple |
| 6[271]4[272] | ACCGATTGTCGGCATTTTCGGTCATAATCA | Core staple |
| 7[248]9[255] | GTTTATTTTGTCACAATCTTACCGAAGCCCTTTAATATCA | Core staple |
| 8[271]6[272] | AATAGCTATCAATAGAAAATTCAACATTCA | Core staple |
| 9[256]11[255] | GAGAGATAGAGCGTCTTTCCAGAGGTTTTGAA | Core staple |
| 10[271]8[272] | ACGCTAACACCCACAAGAATTGAAAATAGC | Core staple |
| 11[256]13[255] | GCCTTAAACCAATCAATAATCGGCACGCGCCT | Core staple |
| 13[128]15[127] | GAGACAGCTAGCTGATAAATTAATTTTTGT | Core staple |
| 0[111]1[95] | TAAATGAATTTTCTGTATGGGATTAATTTCTT | Core staple |
| 1[160]2[144] | TTAGGATTGGCTGAGACTCCTCAATAACCGAT | Core staple |
| 2[175]0[176] | TATTAAGAAGCGGGGTTTTGCTCGTAGCAT | Core staple |
| 4[79]2[80] | GCGCAGACAAGAGGCAAAAGAATCCCTCAG | Core staple |
| 6[47]4[48] | TACGTTAAAGTAATCTTGACAAGAACCGAACT | Core staple |
| 7[32]9[31] | TTTAGGACAAATGCTTTAAACAATCAGGTC | Core staple |
| 8[47]6[48] | ATCCCCCTATACCACATTCAACTAGAAAAATC | Core staple |
| 9[32]11[31] | TTTACCCCAACATGTTTTAAATTTCCATAT | Core staple |
| 10[47]8[48] | CTGTAGCTTGACTATTATAGTCAGTTCATTGA | Core staple |
| 11[32]13[31] | AACAGTTTTGTACCAAAAACATTTTATTTC | Core staple |
| 12[79]10[80] | AAATTAAGTTGACCATTAGATACTTTTGCG | Core staple |
| 13[160]14[144] | GTAATAAGTTAGGCAGAGGCATTTATGATATT | Core staple |
| 0[175]0[144] | TCCACAGACAGCCCTCATAGTTAGCGTAACGA | Core staple |
| 1[192]4[192] | GCGGATAACCTATTATTCTGAAACAGACGATTGGCCTTGAAGAGCCAC | Core staple |
| 2[207]0[208] | TTTCGGAAGTGCCGTCGAGAGGGTGAGTTTCG | Core staple |
| 4[143]3[159] | TCATCGCCAACAAAGTACAACGGACGCCAGCA | Core staple |
| 6[79]4[80] | TTATACCACCAAATCAACGTAACGAACGAG | Core staple |
| 7[56]9[63] | ATGCAGATACATAACGGGAATCGTCATAAATAAAGCAAAG | Core staple |
| 8[79]6[80] | AATACTGCCCAAAAGGAATTACGTGGCTCA | Core staple |
| 9[64]11[63] | CGGATTGCAGAGCTTAATTGCTGAAACGAGTA | Core staple |
| 10[79]8[80] | GATGGCTTATCAAAAAGATTAAGAGCGTCC | Core staple |
| 11[64]13[63] | GATTTAGTCAATAAAGCCTCAGAGAACCCTCA | Core staple |
| 12[143]11[159] | TTCTACTACGCGAGCTGAAAAGGTTACCGCGC | Core staple |
| 13[192]15[191] | GTAAAGTAATCGCCATATTTAACAAAACTTTT | Core staple |
| 0[239]1[223] | AGGAACCCATGTACCGTAACACTTGATATAA | Core staple |
| 1[224]3[223] | GTATAGCAAACAGTTAATGCCCAATCCTCA | Core staple |
| 2[239]0[240] | GCCCGTATCCGGAATAGGTGTATCAGCCCAAT | Core staple |
| 4[207]2[208] | CCACCCTCTATTCACAAACAAATACCTGCCTA | Core staple |
| 6[111]4[112] | ATTACCTTTGAATAAGGCTTGCCCAAATCCGC | Core staple |
| 7[96]9[95] | TAAGAGCAAATGTTTAGACTGGATAGGAAGCC | Core staple |
| 8[111]6[112] | AATAGTAAACACTATCATAACCCTCATTGTGA | Core staple |
| 9[96]11[95] | CGAAAGACTTTGATAAGAGGTCATATTTCGCA | Core staple |
| 10[111]8[112] | TTGCTCCTTTCAAATATCGCGTTTGAGGGGGT | Core staple |
| 11[96]13[95] | AATGGTCAACAGGCAAGGCAAAGAGTAATGTG | Core staple |
| 12[207]10[208] | GTACCGCAATTCTAAGAACGCGAGTATTATTT | Core staple |
| 13[224]15[223] | ACAACATGCCAACGCTCAACAGTCTTCTGA | Core staple |
| 0[271]1[255] | CCACCCTCATTTTCAGGGATAGCAACCGTACT | Core staple |
| 1[256]4[256] | CAGGAGGTGGGGTCAGTGCCTTGAGTCTCTGAATTTACCGGGAACCAG | Core staple |
| 2[271]0[272] | GTTTTAACTTAGTACCGCCACCCAGAGCCA | Core staple |
| 4[271]2[272] | AAATCACCTTCCAGTAAGCGTCAGTAATAA | Core staple |
| 6[143]5[159] | GATGGTTTGAACGAGTAGTAAATTTACCATTA | Core staple |
| 7[120]9[127] | CGTTTACCAGACGACAAAGAAGTTTTGCCATAATTCGA | Core staple |
| 8[143]7[159] | CTTTTGCAGATAAAAACCAAAATAAAGACTCC | Core staple |

| | | |
|---|---|---|
| 9[128]11[127] | GCTTCAATCAGGATTAGAGAGTTATTTTCA | Core staple |
| 10[143]9[159] | CCAACAGGAGCGAACCAGACCGGAGCCTTTAC | Core staple |
| 11[128]13[127] | TTTGGGGATAGTAGTAGCATTAAAAGGCCG | Core staple |
| 12[271]10[272] | TGTAGAAATCAAGATTAGTTGCTCTTACCA | Core staple |
| 13[256]15[255] | GTTTATCAATATGCGTTATACAAACCGACCGT | Core staple |
| 1[32]3[31] | AGGCTCCAGAGGCTTTGAGGACACGGGTAA | Core staple |
| 2[47]0[48] | ACGGCTACAAAAGGAGCCTTTAATGTGAGAAT | Core staple |
| 3[32]5[31] | AATACGTTTGAAAGAGGACAGACTGACCTT | Core staple |
| 5[32]7[31] | CATCAAGTAAAACGAACTAACGAGTTGAGA | Core staple |
| 6[175]4[176] | CAGCAAAAGGAAACGTCACCAATGAGCCGC | Core staple |
| 7[160]8[144] | TTATTACGAAGAACTGGCATGATTGCGAGAGG | Core staple |
| 8[175]6[176] | ATACCCAACAGTATGTTAGCAAATTAGAGC | Core staple |
| 9[160]10[144] | AGAGAGAAAAAAATGAAAATAGCAAGCAAACT | Core staple |
| 10[175]8[176] | TTAACGTCTAACATAAAAACAGGTAACGGA | Core staple |
| 11[160]12[144] | CCAATAGCTCATCGTAGGAATCATGGCATCAA | Core staple |
| 13[32]15[31] | AACGCAAAATCGATGAACGGTACCGGTTGA | Core staple |
| 14[47]12[48] | AACAAGAGGGATAAAAATTTTTAGCATAAAGC | Core staple |
| 1[64]4[64] | TTTATCAGGACAGCATCGGAACGACACCAACCTAAAACGAGGTCAATC | Core staple |
| 2[79]0[80] | CAGCGAAACTTGCTTTCGAGGTGTTGCTAA | Core staple |
| 3[96]5[95] | ACACTCATCCATGTTACTTAGCCGAAAGCTGC | Core staple |
| 5[96]7[95] | TCATTCAGATGCGATTTTAAGAACAGGCATAG | Core staple |
| 6[207]4[208] | TCACCGACGCACCGTAATCAGTAGCAGAACCG | Core staple |
| 7[184]9[191] | CGTAGAAAATACATACCGAGGAAACGCAATAAGAAGCGCA | Core staple |
| 8[207]6[208] | AAGGAAACATAAAGGTGGCAACATTATCACCG | Core staple |
| 9[192]11[191] | TTAGACGGCCAAATAAGAAACGATAGAAGGCT | Core staple |
| 10[207]8[208] | ATCCCAATGAGAATTAACTGAACAGTTACCAG | Core staple |
| 11[192]13[191] | TATCCGGTCTCATCGAGAACAAGCGACAAAAG | Core staple |
| 13[64]15[63] | TATATTTTGTCATTGCCTGAGAGTGGAAGATT | Core staple |
| 14[79]12[80] | GCTATCAGAAATGCAATGCCTGAATTAGCA | Core staple |
| 14[111]12[112] | GAGGGTAGGATTCAAAAGGGTGAGACATCCAA | Core staple |
| 15[96]17[95] | ATATTTTGGCTTTCATCAACATTATCCAGCCA | Core staple |
| 16[111]14[112] | TGTAGCCATTAAAATTCGCATTAAATGCCGGA | Core staple |
| 17[224]19[223] | CATAAATCTTTGAATACCAAGTGTTAGAAC | Core staple |
| 19[32]21[31] | GTCGACTTCGGCCAACGCGCGGGGTTTTTC | Core staple |
| 21[56]23[63] | AGCTGATTGCCCTTCAGAGTCCACTATTAAAGGGTGCCGT | Core staple |
| 22[79]20[80] | TGGAACAACCGCCTGGCCCTGAGGCCCGCT | Core staple |
| 23[96]22[112] | CCCGATTTAGAGCTTGACGGGGAAAAAGAATA | Core staple |
| 16[271]14[272] | CTTAGATTTAAGGCGTTAAATAAAGCCTGT | Core staple |
| 14[143]13[159] | CAACCGTTTCAAATCACCATCAATTCGAGCCA | Core staple |
| 15[128]18[128] | TAAATCAAAATAATTCGCGTCTCGGAAACCAGGCAAAGGGAAGG | Core staple |
| 16[143]15[159] | GCCATCAAGCTCATTTTTTAACCACAAATCCA | Core staple |
| 18[47]16[48] | CCAGGGTTGCCAGTTTGAGGGGACCCGTGGGA | Core staple |
| 19[96]21[95] | CTGTGTGATTGCGTTGCGCTCACTAGAGTTGC | Core staple |
| 21[96]23[95] | AGCAAGCGTAGGGTTGAGTGTTGTAGGGAGCC | Core staple |
| 22[111]20[112] | GCCCGAGAGTCCACGCTGGTTTGCAGCTAACT | Core staple |
| 23[128]23[159] | AACGTGGCGAGAAAGGAAGGGAAACCAGTAA | Core staple |
| 18[271]16[272] | CTTTTACAAAATCGTCGCTATTAGCGATAG | Core staple |
| 14[175]12[176] | CATGTAATAGAATATAAAGTACCAAGCCGT | Core staple |
| 15[160]16[144] | ATCGCAAGTATGTAAATGCTGATGATAGGAAC | Core staple |
| 16[175]14[176] | TATAACTAACAAAGAACGCGAGAACGCCAA | Core staple |
| 18[79]16[80] | GATGTGCTTCAGGAAGATCGCACAATGTGA | Core staple |
| 19[160]20[144] | GCAATTCACATATTCCTGATTATCAAAGTGTA | Core staple |
| 21[120]23[127] | CCCAGCAGGCGAAAAATCCCTTATAAATCAAGCCGGCG | Core staple |
| 22[143]21[159] | TCGGCAAATCCTGTTTGATGGTGGACCCTCAA | Core staple |
| 23[160]22[176] | TAAAAGGGACATTCTGGCCAACAAAGCATC | Core staple |
| 20[271]18[272] | CTCGTATTAGAAATTGCGTAGATACAGTAC | Core staple |
| 14[207]12[208] | AATTGAGAATTCTGTCCAGACGACTAAACCAA | Core staple |
| 15[192]18[192] | TCAAATATAACCTCCGGCTTAGGTAACAATTTCATTTGAAGGCGAATT | Core staple |
| 16[207]14[208] | ACCTTTTTATTTTAGTTAATTTCATAGGGCTT | Core staple |
| 18[111]16[112] | TCTTCGCTGCACCGCTTCTGGTGCGGCCTTCC | Core staple |
| 19[224]21[223] | CTACCATAGTTTGAGTAACATTTAAAATAT | Core staple |
| 21[160]22[144] | TCAATATCGAACCTCAAATATCAATTCCGAAA | Core staple |
| 22[175]20[176] | ACCTTGCTTGGTCAGTTGGCAAAGAGCGGA | Core staple |
| 23[192]22[208] | ACCCTTCTGACCTGAAAGCGTAAGACGCTGAG | Core staple |
| 22[271]20[272] | CAGAAGATTAGATAATACATTTGTCGACAA | Core staple |
| 14[239]12[240] | AGTATAAAGTTCAGCTAATGCAGATGTCTTTC | Core staple |
| 15[224]17[223] | CCTAAATCAAAATCATAGGTCTAAACAGTA | Core staple |
| 16[239]14[240] | GAATTTATTTAATGGTTTGAAATATTCTTACC | Core staple |
| 18[143]17[159] | CAACTGTTGCGCCATTCGCCATTCAAACATCA | Core staple |
| 20[79]18[80] | TTCCAGTCGTAATCATGGTCATAAAAGGGG | Core staple |

| | | |
|---|---|---|
| 21[184]23[191] | TCAACAGTTGAAAGGAGCAAATGAAAAATCTAGAGATAGA | Core staple |
| 22[207]20[208] | AGCCAGCAATTGAGGAAGGTTATCATCATTTT | Core staple |
| 23[224]22[240] | GCACAGACAATATTTTTGAATGGGGTCAGTA | Core staple |
| 14[271]12[272] | TTAGTATCACAATAGATAAGTCCACGAGCA | Core staple |
| 15[256]18[256] | GTGATAAAAAGACGCTGAGAAGAGATAACCTTGCTTCTGTTCGGGAGA | Core staple |
| 17[32]19[31] | TGCATCTTTCCCAGTCACGACGGCCTGCAG | Core staple |
| 18[175]16[176] | CTGAGCAAAAATTAATTACATTTTGGGTTA | Core staple |
| 20[143]19[159] | AAGCCTGGTACGAGCCGGAAGCATAGATGATG | Core staple |
| 21[224]23[223] | CTTTAGGGCCTGCAACAGTGCCAATACGTG | Core staple |
| 22[239]20[240] | TTAACACCAGCACTAACAACTAATCGTTATTA | Core staple |
| 23[256]22[272] | CTTTAATGCGCGAACTGATAGCCCCACCAG | Core staple |
| 15[32]17[31] | TAATCAGCGGATTGACCGTAATCGTAACCG | Core staple |
| 16[47]14[48] | ACAAACGGAAAAGCCCCAAAAACACTGGAGCA | Core staple |
| 17[96]19[95] | GCTTTCCGATTACGCCAGCTGGCGGCTGTTTC | Core staple |
| 18[207]16[208] | CGCGCAGATTACCTTTTTTAATGGGAGAGACT | Core staple |
| 20[207]18[208] | GCGGAACATCTGAATAATGGAAGGTACAAAAT | Core staple |
| 21[248]23[255] | AGATTAGAGCCGTCAAAAAACAGAGGTGAGGCCTATTAGT | Core staple |
| 23[32]22[48] | CAAATCAAGTTTTTTGGGGTCGAAACGTGGA | Core staple |
| 0[143]1[127] | TCTAAAGTTTTGTCGTCTTTCCAGCCGACAA | Core staple |
| 15[64]18[64] | GTATAAGCCAACCCGTCGGATTCTGACGACAGTATCGGCCGCAAGGCG | Core staple |
| 16[79]14[80] | GCGAGTAAAAATATTTAAATTGTTACAAAG | Core staple |
| 17[160]18[144] | AGAAAACAAAGAAGATGATGAAACAGGCTGCG | Core staple |
| 18[239]16[240] | CCTGATTGCAATATATGTGAGTGATCAATAGT | Core staple |
| 21[32]23[31] | TTTTCACTCAAAGGGCGAAAAACCATCACC | Core staple |
| 22[47]20[48] | CTCCAACGCAGTGAGACGGGCAACCAGCTGCA | Core staple |
| 23[64]22[80] | AAAGCACTAAATCGGAACCCTAATCCAGTT | Core staple |
| 0[207]1[191] | TCACCAGTACAAACTACAACGCCTAGTACCAG | Core staple |
| 4[47]2[48] | GACCAACTAATGCCACTACGAAGGGGGTAGCA | Core staple |
| 20[47]18[48] | TTAATGAACTAGAGGATCCCCGGGGGGTAACG | Core staple |
| 4[111]2[112] | GACCTGCTCTTTGACCCCCAGCGAGGGAG | Core staple |
| 20[111]18[112] | CACATTAAAATTGTTATCCGCTCATGCGGGCC | Core staple |
| 4[175]2[176] | CACCAGAAAGGTTGAGGCAGGTCATGAAAG | Core staple |
| 20[175]18[176] | ATTATCATTCAATATAATCCTGACAATTAC | Core staple |
| 4[239]2[240] | GCCTCCCTCAGAATGGAAAGCGCAGTAACAGT | Core staple |
| 20[239]18[240] | ATTTTAAAATCAAAATTATTTGCACGGATTCG | Core staple |
| 12[47]10[48] | TAAATCGGGATTCCCAATTCTGCGATATAATG | Core staple |
| 12[111]10[112] | TAAATCATATAACCTGTTTAGCTAACCTTTAA | Core staple |
| 12[175]10[176] | TTTTATTTAAGCAAATCAGATATTTTTTGT | Core staple |
| 12[239]10[240] | CTTATCATTCCCGACTTGCGGGAGCCTAATT | Core staple |
| 4[63]6[56] | TTTTATAAGGGAACCGGATATTCATTACGTCAGGACGTTGGGAA | Core staple |
| 4[127]6[120] | TTTTTTGTGTCGTGACGAGAAACACCAAATTTCAACTTTAAT | Core staple |
| 4[191]6[184] | TTTTCACCCTCAGAAACCATCGATAGCATTGAGCCATTTGGGAA | Core staple |
| 4[255]6[248] | TTTTAGCCACCACTGTAGCGCGTTTTCAAGGGAGGGAAGGTAAA | Core staple |
| 18[63]20[56] | TTTTATTAAGTTTACCGAGCTCGAATTCGGGAAACCTGTCGTGC | Core staple |
| 18[127]20[120] | TTTTGCGATCGGCAATTCCACACAACAGGTGCCTAATGAGTG | Core staple |
| 18[191]20[184] | TTTTATTCATTTTTGTTTGGATTATACTAAGAAACCACCAGAAG | Core staple |
| 18[255]20[248] | TTTTAACAATAACGTAAAACAGAAATAAAAATCCTTTGCCCGAA | Core staple |
| biotin-4[63]6[56] | /5Biosg/TTTTATAAGGGAACCGGATATTCATTACGTCAGGACGTTGGGAA | Biotinylated staple |
| biotin-4[127]6[120] | /5Biosg/TTTTTTGTGTCGTGACGAGAAACACCAAATTTCAACTTTAAT | Biotinylated staple |
| biotin-4[191]6[184] | /5Biosg/TTTTCACCCTCAGAAACCATCGATAGCATTGAGCCATTTGGGAA | Biotinylated staple |
| biotin-4[255]6[248] | /5Biosg/TTTTAGCCACCACTGTAGCGCGTTTTCAAGGGAGGGAAGGTAAA | Biotinylated staple |
| biotin-18[63]20[56] | /5Biosg/TTTTATTAAGTTTACCGAGCTCGAATTCGGGAAACCTGTCGTGC | Biotinylated staple |
| biotin-18[127]20[120] | /5Biosg/TTTTGCGATCGGCAATTCCACACAACAGGTGCCTAATGAGTG | Biotinylated staple |
| biotin-18[191]20[184] | /5Biosg/TTTTATTCATTTTTGTTTGGATTATACTAAGAAACCACCAGAAG | Biotinylated staple |
| biotin-18[255]20[248] | /5Biosg/TTTTAACAATAACGTAAAACAGAAATAAAAATCCTTTGCCCGAA | Biotinylated staple |
| 4[47]2[48]-2T-R1 | GACCAACTAATGCCACTACGAAGGGGGTAGCATTTCCTCCTCCTCCTCCTCCT | 20 nm docking strand with 32 nt binder |
| 20[47]18[48]-2T-R1 | TTAATGAACTAGAGGATCCCCGGGGGGTAACGTTTCCTCCTCCTCCTCCTCCT | 20 nm docking strand with 32 nt binder |
| 4[111]2[112]-2T-R1 | GACCTGCTCTTTGACCCCCAGCGAGGGAGTTATTTCCTCCTCCTCCTCCTCCT | 20 nm docking strand with 32 nt binder |
| 20[111]18[112]-2T-R1 | CACATTAAAATTGTTATCCGCTCATGCGGGCCTTTCCTCCTCCTCCTCCTCCT | 20 nm docking strand with 32 nt binder |
| 4[175]2[176]-2T-R1 | CACCAGAAAGGTTGAGGCAGGTCATGAAAGTTTCCTCCTCCTCCTCCTCCT | 20 nm docking strand with 32 nt binder |
| 20[175]18[176]-2T-R1 | ATTATCATTCAATATAATCCTGACAATTACTTTCCTCCTCCTCCTCCTCCT | 20 nm docking strand with 32 nt binder |
| 4[239]2[240]-2T-R1 | GCCTCCCTCAGAATGGAAAGCGCAGTAACAGTTTTCCTCCTCCTCCTCCTCCT | 20 nm docking strand with 32 nt binder |
| 20[239]18[240]-2T-R1 | ATTTTAAAATCAAAATTATTTGCACGGATTCGTTTCCTCCTCCTCCTCCTCCT | 20 nm docking strand with 32 nt binder |

| | | |
|---|---|---|
| 12[47]10[48]-2T-R1 | TAAATCGGGATTCCCAATTCTGCGATATAATGTTTCCTCCTCCTCCTCCTCCT | 20 nm docking strand with 32 nt binder |
| 12[111]10[112]-2T-R1 | TAAATCATATAACCTGTTTAGCTAACCTTTAATTTCCTCCTCCTCCTCCTCCT | 20 nm docking strand with 32 nt binder |
| 12[175]10[176]-2T-R1 | TTTTATTTAAGCAAATCAGATATTTTTTGTTTTCCTCCTCCTCCTCCTCCT | 20 nm docking strand with 32 nt binder |
| 12[239]10[240]-2T-R1 | CTTATCATTCCCGACTTGCGGGAGCCTAATTTTTTCCTCCTCCTCCTCCTCCT | 20 nm docking strand with 32 nt binder |
| 6[47]2[48]-2T-R1 | TACGTTAAAGTAATCTTGACAAGAACCGAACTGACCAACTAATGCCACTACGAAGGGGGTAGCATTTCCTCCTCCTCCTCCTCCT | 10 nm, 14 nm, and 20 nm docking strand with 64 nt binder |
| 14[47]10[48]-2T-R1 | AACAAGAGGGATAAAAATTTTTAGCATAAAGCTAAATCGGGATTCCCAATTCTGCGATATAATGTTTCCTCCTCCTCCTCCTCCT | 10 nm, 14 nm, and 20 nm docking strand with 64 nt binder |
| 22[47]18[48]-2T-R1 | CTCCAACGCAGTGAGACGGGCAACCAGCTGCATTAATGAACTAGAGGATCCCCGGGGGGTAACGTTTCCTCCTCCTCCTCCTCCT | 10 nm, 14 nm, and 20 nm docking strand with 64 nt binder |
| 6[111]2[112]-2T-R1 | ATTACCTTTGAATAAGGCTTGCCCAAATCCGCGACCTGCTCTTTGACCCCCAGCGAGGGAGTTATTTCCTCCTCCTCCTCCTCCT | 10 nm, 14 nm, and 20 nm docking strand with 64 nt binder |
| 14[111]10[112]-2T-R1 | GAGGGTAGGATTCAAAAGGGTGAGACATCCAATAAATCATATAACCTGTTTAGCTAACCTTTAATTTCCTCCTCCTCCTCCTCCT | 10 nm, 14 nm, and 20 nm docking strand with 64 nt binder |
| 22[111]18[112]-2T-R1 | GCCCGAGAGTCCACGCTGGTTTGCAGCTAACTCACATTAAAATTGTTATCCGCTCATGCGGGCCTTTCCTCCTCCTCCTCCTCCT | 10 nm, 14 nm, and 20 nm docking strand with 64 nt binder |
| 6[175]2[176]-2T-R1 | CAGCAAAAGGAAACGTCACCAATGAGCCGCCACCAGAAAGGTTGAGGCAGGTCATGAAAGTTTCCTCCTCCTCCTCCTCCT | 10 nm, 14 nm, and 20 nm docking strand with 64 nt binder |
| 14[175]10[176]-2T-R1 | CATGTAATAGAATATAAAGTACCAAGCCGTTTTTATTTAAGCAAATCAGATATTTTTTGTTTTCCTCCTCCTCCTCCTCCT | 10 nm, 14 nm, and 20 nm docking strand with 64 nt binder |
| 22[175]18[176]-2T-R1 | ACCTTGCTTGGTCAGTTGGCAAAGAGCGGAATTATCATTCAATATAATCCTGACAATTACTTTCCTCCTCCTCCTCCTCCT | 10 nm, 14 nm, and 20 nm docking strand with 64 nt binder |
| 6[239]2[240]-2T-R1 | GAAATTATTGCCTTTAGCGTCAGACCGGAACCGCCTCCCTCAGAATGGAAAGCGCAGTAACAGTTTTCCTCCTCCTCCTCCTCCT | 10 nm, 14 nm, and 20 nm docking strand with 64 nt binder |
| 14[239]10[240]-2T-R1 | AGTATAAAGTTCAGCTAATGCAGATGTCTTTCCTTATCATTCCCGACTTGCGGGAGCCTAATTTTTTCCTCCTCCTCCTCCTCCT | 10 nm, 14 nm, and 20 nm docking strand with 64 nt binder |
| 22[239]18[240]-2T-R1 | TTAACACCAGCACTAACAACTAATCGTTATTAATTTTAAAATCAAAATTATTTGCACGGATTCGTTTCCTCCTCCTCCTCCTCCT | 10 nm, 14 nm, and 20 nm docking strand with 64 nt binder |
| 10[47]6[48]-2T-R1 | CTGTAGCTTGACTATTATAGTCAGTTCATTGAATCCCCCTATACCACATTCAACTAGAAAAATCTTTCCTCCTCCTCCTCCTCCT | 10 nm, and 14 nm docking strand with 64 nt binder |
| 18[79]14[80]-2T-R1 | GATGTGCTTCAGGAAGATCGCACAATGTGAGCGAGTAAAAATATTTAAATTGTTACAAAGTTTCCTCCTCCTCCTCCTCCT | 10 nm, and 14 nm docking strand with 64 nt binder |
| 21[56]22[80]-2T-R1 | GCTGATTGCCCTTCAGAGTCCACTATTAAAGGGTGCCGTAAAGCACTAAATCGGAACCCTAATCCAGTTTTTCCTCCTCCTCCTCCTCCT | 10 nm, and 14 nm docking strand with 64 nt binder |
| 6[143]6[144]-2T-R1 | GATGGTTTGAACGAGTAGTAAATTTACCATTAGCAAGGCCTCACCAGTAGCACCATGGGCTTGATTTCCTCCTCCTCCTCCTCCT | 10 nm, and 14 nm docking strand with 64 nt binder |
| 14[143]14[144]-2T-R1 | CAACCGTTTCAAATCACCATCAATTCGAGCCAGTAATAAGTTAGGCAGAGGCATTTATGATATTTTTCCTCCTCCTCCTCCTCCT | 10 nm, and 14 nm docking strand with 64 nt binder |
| 22[143]22[144]-2T-R1 | TCGGCAAATCCTGTTTGATGGTGGACCCTCAATCAATATCGAACCTCAAATATCAATTCCGAAATTTCCTCCTCCTCCTCCTCCT | 10 nm, and 14 nm docking strand with 64 nt binder |
| 10[207]6[208]-2T-R1 | ATCCCAATGAGAATTAACTGAACAGTTACCAGAAGGAAACATAAAGGTGGCAACATTATCACCGTTTCCTCCTCCTCCTCCTCCT | 10 nm, and 14 nm docking strand with 64 nt binder |
| 18[207]14[208]-2T-R1 | CGCGCAGATTACCTTTTTTAATGGGAGAGACTACCTTTTTATTTTAGTTAATTTCATAGGGCTTTTTCCTCCTCCTCCTCCTCCT | 10 nm, and 14 nm docking strand with 64 nt binder |
| 21[184]22[208]-2T-R1 | ACAGTTGAAAGGAGCAAATGAAAAATCTAGAGATAGAACCCTTCTGACCTGAAAGCGTAAGACGCTGAGTTTCCTCCTCCTCCTCCTCCT | 10 nm, and 14 nm docking strand with 64 nt binder |
| 10[271]6[272]-2T-R1 | ACGCTAACACCCACAAGAATTGAAAATAGCAATAGCTATCAATAGAAAATTCAACATTCATTTCCTCCTCCTCCTCCTCCT | 10 nm, and 14 nm docking strand with 64 nt binder |
| 18[271]14[272]-2T-R1 | CTTTTACAAAATCGTCGCTATTAGCGATAGCTTAGATTTAAGGCGTTAAATAAAGCCTGTTTTCCTCCTCCTCCTCCTCCT | 10 nm, and 14 nm docking strand with 64 nt binder |
| 21[248]22[272]-2T-R1 | GATTAGAGCCGTCAAAAAACAGAGGTGAGGCCTATTAGTCTTTAATGCGCGAACTGATAGCCCCACCAGTTTCCTCCTCCTCCTCCTCCT | 10 nm, and 14 nm docking strand with 64 nt binder |
| 10[111]6[112]-2T-R1 | TTGCTCCTTTCAAATATCGCGTTTGAGGGGGTAATAGTAAACACTATCATAACCCTCATTGTGATTTCCTCCTCCTCCTCCTCCT | 10 nm docking strand with 64 nt binder |

| | | |
|---|---|---|
| 10[143]10[144]-2T-R1 | CCAACAGGAGCGAACCAGACCGGAGCCTTTACAGAGAGAAAAAAATGAA AATAGCAAGCAAACTTTTCCTCCTCCTCCTCCT | 10 nm docking strand with 64 nt binder |
| 10[175]6[176]-2T-R1 | TTAACGTCTAACATAAAAACAGGTAACGGAATACCCAACAGTATGTTAG CAAATTAGAGCTTTCCTCCTCCTCCTCCT | 10 nm docking strand with 64 nt binder |
| 10[239]6[240]-2T-R1 | GCCAGTTAGAGGGTAATTGAGCGCTTTAAGAAAAGTAAGCAGACACCAC GGAATAATATTGACGTTTCCTCCTCCTCCTCCT | 10 nm docking strand with 64 nt binder |
| 14[207]10[208]-2T-R1 | AATTGAGAATTCTGTCCAGACGACTAAACCAAGTACCGCAATTCTAAGA ACGCGAGTATTATTTTTTCCTCCTCCTCCTCCT | 10 nm docking strand with 64 nt binder |
| 14[271]10[272]-2T-R1 | TTAGTATCACAATAGATAAGTCCACGAGCATGTAGAAATCAAGATTAGT TGCTCTTACCATTTCCTCCTCCTCCTCCT | 10 nm docking strand with 64 nt binder |
| 14[79]10[80]-2T-R1 | GCTATCAGAAATGCAATGCCTGAATTAGCAAAATTAAGTTGACCATTAG ATACTTTTGCGTTTCCTCCTCCTCCTCCT | 10 nm docking strand with 64 nt binder |
| 18[111]14[112]-2T-R1 | TCTTCGCTGCACCGCTTCTGGTGCGGCCTTCCTGTAGCCATTAAAATTC GCATTAAATGCCGGATTTCCTCCTCCTCCTCCT | 10 nm docking strand with 64 nt binder |
| 18[143]18[144]-2T-R1 | CAACTGTTGCGCCATTCGCCATTCAAACATCAAGAAAACAAAGAAGATG ATGAAACAGGCTGCGTTTCCTCCTCCTCCTCCT | 10 nm docking strand with 64 nt binder |
| 18[175]14[176]-2T-R1 | CTGAGCAAAAATTAATTACATTTTGGGTTATATAACTAACAAAGAACGC GAGAACGCCAATTTCCTCCTCCTCCTCCT | 10 nm docking strand with 64 nt binder |
| 18[239]14[240]-2T-R1 | CCTGATTGCAATATATGTGAGTGATCAATAGTGAATTTATTTAATGGTT TGAAATATTCTTACCTTTCCTCCTCCTCCTCCT | 10 nm docking strand with 64 nt binder |
| 18[47]14[48]-2T-R1 | CCAGGGTTGCCAGTTTGAGGGGACCCGTGGGAACAAACGGAAAAGCCCC AAAAACACTGGAGCATTTCCTCCTCCTCCTCCT | 10 nm docking strand with 64 nt binder |
| 2[143]2[144]-2T-R1 | ATATTCGGAACCATCGCCCACGCAGAGAAGGATTAGGATTGGCTGAGAC TCCTCAATAACCGATTTTCCTCCTCCTCCTCCT | 10 nm docking strand with 64 nt binder |
| 21[184]22[208]-2T-R1 | ACAGTTGAAAGGAGCAAATGAAAAATCTAGAGATAGAACCCTTCTGACC TGAAAGCGTAAGACGCTGAGTTTCCTCCTCCTCCTCCT | 10 nm docking strand with 64 nt binder |
| 21[224]22[240]-2T-R1 | CTTTAGGGCCTGCAACAGTGCCAATACGTGGCACAGACAATATTTTTGA ATGGGGTCAGTATTTCCTCCTCCTCCTCCT | 10 nm docking strand with 64 nt binder |
| 21[32]22[48]-2T-R1 | TTTTCACTCAAAGGGCGAAAAACCATCACCCAAATCAAGTTTTTTGGGG TCGAAACGTGGATTTCCTCCTCCTCCTCCT | 10 nm docking strand with 64 nt binder |
| 21[96]22[112]-2T-R1 | AGCAAGCGTAGGGTTGAGTGTTGTAGGGAGCCCCCGATTTAGAGCTTGA CGGGGAAAAAGAATATTTCCTCCTCCTCCTCCT | 10 nm docking strand with 64 nt binder |
| 22[207]18[208]-2T-R1 | AGCCAGCAATTGAGGAAGGTTATCATCATTTTGCGGAACATCTGAATAA TGGAAGGTACAAAATTTTCCTCCTCCTCCTCCT | 10 nm docking strand with 64 nt binder |
| 22[271]18[272]-2T-R1 | CAGAAGATTAGATAATACATTTGTCGACAACTCGTATTAGAAATTGCGT AGATACAGTACTTTCCTCCTCCTCCTCCT | 10 nm docking strand with 64 nt binder |
| 22[79]18[80]-2T-R1 | TGGAACAACCGCCTGGCCCTGAGGCCCGCTTTCCAGTCGTAATCATGGT CATAAAAGGGGTTTCCTCCTCCTCCTCCT | 10 nm docking strand with 64 nt binder |
| 23[128]22[176]-2T-R1 | AACGTGGCGAGAAAGGAAGGGAAACCAGTAATAAAAGGGACATTCTGG CCAACAAAGCATCTTTCCTCCTCCTCCTCCT | 10 nm docking strand with 64 nt binder |
| 6[207]2[208]-2T-R1 | TCACCGACGCACCGTAATCAGTAGCAGAACCGCCACCCTCTATTCACAA ACAAATACCTGCCTATTTCCTCCTCCTCCTCCT | 10 nm docking strand with 64 nt binder |
| 6[271]2[272]-2T-R1 | ACCGATTGTCGGCATTTTCGGTCATAATCAAAATCACCTTCCAGTAAGC GTCAGTAATAATTTCCTCCTCCTCCTCCT | 10 nm docking strand with 64 nt binder |
| 6[79]2[80]-2T-R1 | TTATACCACCAAATCAACGTAACGAACGAGGCGCAGACAAGAGGCAAAA GAATCCCTCAGTTTCCTCCTCCTCCTCCT | 10 nm docking strand with 64 nt binder |

**Supplementary Table 1 2D RRO strands.** Scaffold is MP13mp18. Core staples are all the strands that form the structure. Biotinylated staples were modified with biotin to immobilize origami on coverslip surfaces through a BSA-Biotin-Streptavidin-Biotin-DNA origami arrangement. To fold a 20 nm pattern on 2D RRO with 64 binders, we mixed scaffold strands; the 10 nm, 14 nm, and 20 nm strands with 64 nt binders along with core staples (positions corresponding to 20 nm docking positions and biotin positions should be excluded beforehand) and biotinylated staple strands. To fold a 14 nm pattern on 2D RRO with 64 binder, we mixed scaffold strands, the 10 nm, 14 nm, and 20 nm strands with 64 nt binder, the 10 nm, and 14 nm docking strands with 64 nt binder, along with core staples (positions corresponding to 20 nm docking positions and biotin positions should be excluded beforehand). To fold a 10 nm pattern on 2D RRO with 64 binder, we mix scaffold strands, the 10 nm, 14 nm, and 20 nm strands with 64 nt binder, the 10 nm, and 14 nm docking strands with 64 nt binder, the 10 nm docking strands with 64 nt binder along with core staples (positions corresponding to 20 nm docking positions and biotin positions should be excluded beforehand).

| Name | Sequence | **Note** |
|---|---|---|
| 06_cuboctahedron_147_1-1913-V | ATCACCGTACTTTTTTCAGGAGGTTTTAAAGATTCAATTTTTAAGGGTGAGA | Core staple |
| 06_cuboctahedron_147_1-4390-E | CAGTAACAGTAGTATAGCCCGGAATAGGTGTAGATGAATATA | Core staple |
| 06_cuboctahedron_147_1-4369-E | CGGGAGAAACGCCGTCGAGAGGGTTGATATAACCTTTTACAT | Core staple |
| 06_cuboctahedron_147_1-4348-E | CGCCTGATTGCTCAGTACCAGGCGGATAAGTAATAACGGATT | Core staple |
| 06_cuboctahedron_147_1-4327-E | AAGTTACAAAATTAGGATTAGCGGGGTTTTGCTTTGAATACC | Core staple |
| 06_cuboctahedron_147_1-4306-E | GCGAATTATTCTGAGACTCCTCAAGAGAAGGATCGCGCAGAG | Core staple |
| 06_cuboctahedron_147_1-4285-E | CCTGAGCAAAAAACATGAAAGTATTAAGAGGCATTTCAATTA | Core staple |
| 06_cuboctahedron_147_2-2060-V | ATTTCGGAACCTTTTTTATTATTCTGAGAAGATGATGTTTTTAAACAAACAT | Core staple |
| 06_cuboctahedron_147_2-1609-V | ACTAAAGGAATTTTTTTGCGAATAATGTTTAATTTCATTTTTACTTTAATCA | Core staple |
| 06_cuboctahedron_147_2-1669-E | GTATGGGACAGACGTTAGTAAATGTAACGGGG | Core staple |
| 06_cuboctahedron_147_2-2110-E | TCAGTGCCTACTGGTAATAAGTTTAATTTTCT | Core staple |
| 06_cuboctahedron_147_2-2165-E | GTCATACATGAACAGTTAATGCCCCCTGCCTTTCCAGTAAGC | Core staple |
| 06_cuboctahedron_147_2-2144-E | TACAGGAGTGTTGAGTAACAGTGCCCGTATAGCTTTTGATGA | Core staple |
| 06_cuboctahedron_147_2-1650-E | AACTTTCAACTCTAAAGTTTTGTCGTCTTTCTTTTGCTAAAC | Core staple |
| 06_cuboctahedron_147_2-1629-E | AGTGAGAATACCCTCATAGTTAGCGTAACGAAGTTTCAGCGG | Core staple |
| 06_cuboctahedron_147_2-1619-E | GAAAGGAACACCACAGACAG | Core staple |
| 06_cuboctahedron_147_3-5755-V | AGGGCGAAAAATTTTTCCGTCTATCATAGATTTTCAGTTTTTGTTTAACGTC | Core staple |
| 06_cuboctahedron_147_3-7016-E | CAGTCAAATCGAACGTGGACTCCAACGTCAAAAGGCCGGAGA | Core staple |
| 06_cuboctahedron_147_3-6995-E | GATATTCAACGAACAAGAGTCCACTATTAAAACCATCAATAT | Core staple |
| 06_cuboctahedron_147_3-6974-E | ATAAATTAATGTTGAGTGTTGTTCCAGTTTGCGTTCTAGCTG | Core staple |
| 06_cuboctahedron_147_3-6953-E | TAGCTATTTTAAAAGAATAGCCCGAGATAGGGCCGGAGAGGG | Core staple |
| 06_cuboctahedron_147_3-6932-E | CAAAGGCTATCGGCAAAATCCCTTATAAATCTGAGAGATCTA | Core staple |
| 06_cuboctahedron_147_3-6911-E | CTGAGAGTCTGTTTGATGGTGGTTCCGAAATCAGGTCATTGC | Core staple |
| 06_cuboctahedron_147_4-5902-V | GCCCCAGCAGGTTTTTCGAAAATCCTGGAGCAAACAATTTTTGAGAATCGAT | Core staple |
| 06_cuboctahedron_147_4-5451-V | TTCCTCGTTAGTTTTTAATCAGAGCGACATTTGAGGATTTTTTTTAGAAGTA | Core staple |
| 06_cuboctahedron_147_4-5511-E | CTTAATGCCGCGTAACCACCACACTGATTGCC | Core staple |
| 06_cuboctahedron_147_4-5952-E | CTTCACCGTGAGACGGGCAACAGCCCGCCGCG | Core staple |
| 06_cuboctahedron_147_4-6007-E | TGGGCGCCAGGCAAGCGGTCCACGCTGGTTTGGTTTGCGTAT | Core staple |
| 06_cuboctahedron_147_4-5986-E | TTTTCACCAGCCTGGCCCTGAGAGAGTTGCAGGTGGTTTTTC | Core staple |
| 06_cuboctahedron_147_4-5492-E | GCGCGTACTAGCAAGTGTAGCGGTCACGCTGGCCGCTACAGG | Core staple |
| 06_cuboctahedron_147_4-5471-E | ACGAGCACGTGGAGCGGGCGCTAGGGCGCTGTGGTTGCTTTG | Core staple |
| 06_cuboctahedron_147_4-5461-E | ATAACGTGCTGAAAGCGAAA | Core staple |
| 06_cuboctahedron_147_5-3530-V | TAGAAACCAATTTTTTCAATAATCGGGCGCAGTCTCTTTTTTGAATTTACCG | Core staple |
| 06_cuboctahedron_147_5-4128-V | TTCCCTTAGAATTTTTTCCTTGAAAAAATCGCAAGACTTTTTAAAGAACGCG | Core staple |
| 06_cuboctahedron_147_5-4243-E | AATTAATTACCCATCCTAATTTACGAGCATGCAAGAAAACAA | Core staple |

| | | |
|---|---|---|
| 06_cuboctahedron_147_5-4222-E | TCATTTGAATCTGAACAAGAAAAATAATATCATTTAACAATT | Core staple |
| 06_cuboctahedron_147_5-4201-E | ATGGAAACAGTTTATCAACAATAGATAAGTCTACCTTTTTA | Core staple |
| 06_cuboctahedron_147_5-4180-E | ATATATGTGAAGCTAATGCAGAACGCGCCTGTACATAAATCA | Core staple |
| 06_cuboctahedron_147_5-4159-E | TGCTTCTGTAACGACAATAAACAACATGTTCGTGAATAACCT | Core staple |
| 06_cuboctahedron_147_5-4138-E | TTAATTAATTTAAAGTAATTCTGTCCAGACGAATCGTCGCTA | Core staple |
| 06_cuboctahedron_147_6-1462-V | GACAACAACCATTTTTTCGCCCACGCTTCATGAGGAATTTTTGTTTCCATTA | Core staple |
| 06_cuboctahedron_147_6-1577-E | GTTGAAAATCTAGTAAATTGGGCTTGAGATGAATTTTTTCAC | Core staple |
| 06_cuboctahedron_147_6-1556-E | GGCTCCAAAAGACGAGAAACACCAGAACGAGTCCAAAAAAAA | Core staple |
| 06_cuboctahedron_147_6-1535-E | TTGTATCGGTTCAGTGAATAAGGCTTGCCCTGGAGCCTTTAA | Core staple |
| 06_cuboctahedron_147_6-1514-E | CTTTCGAGGTCAACGTAACAAAGCTGCTCATTTATCAGCTTG | Core staple |
| 06_cuboctahedron_147_6-1493-E | ACAGCTTGATCCGGATATTCATTACCCAAATGAATTTCTTAA | Core staple |
| 06_cuboctahedron_147_6-1472-E | CGCCGACAATCAAGAGTAATCTTGACAAGAAACCGATAGTTG | Core staple |
| 06_cuboctahedron_147_7-6764-V | TGTTAAAATTCTTTTTGCATTAAATTGGCCAACGCGCTTTTTGGGGAGAGGC | Core staple |
| 06_cuboctahedron_147_7-286-V | AGAGTACCTTTTTTTTAATTGCTCCTTTGAGATTTAGTTTTTGAATACCACA | Core staple |
| 06_cuboctahedron_147_7-346-E | TTTTAATTGCCCGAAAGACTTCAAAGCCCCAA | Core staple |
| 06_cuboctahedron_147_7-6814-E | AAACAGGAGGTTGATAATCAGAAAATATCGCG | Core staple |
| 06_cuboctahedron_147_7-6869-E | GTAAAACTAGAAATTGTAAACGTTAATATTTGAACGGTAATC | Core staple |
| 06_cuboctahedron_147_7-6848-E | TATGTACCCCAGATTGTATAAGCAAATATTTCATGTCAATCA | Core staple |
| 06_cuboctahedron_147_7-327-E | GCGAACCAGACATCAAAAAGATTAAGAGGAACGAGCTTCAAA | Core staple |
| 06_cuboctahedron_147_7-306-E | CTCCAACAGGAGTCAGAAGCAAAGCGGATTGCCGGAAGCAAA | Core staple |
| 06_cuboctahedron_147_7-296-E | TCAGGATTAGTGACTATTAT | Core staple |
| 06_cuboctahedron_147_8-6460-V | AACCAGGCAAATTTTTGCGCCATTCGTTCCCAGTCACTTTTTGACGTTGTAA | Core staple |
| 06_cuboctahedron_147_8-6520-E | GTATCGGCTGCCAGTTTGAGGGGAATTCATTG | Core staple |
| 06_cuboctahedron_147_8-493-E | AATCCCCCCGGAATCGTCATAAATCGACGACA | Core staple |
| 06_cuboctahedron_147_8-548-E | AAATGTTTAGGAAAACGAGAATGACCATAAAGGGTAATAGTA | Core staple |
| 06_cuboctahedron_147_8-527-E | TCCAATACTGTCAAATGCTTTAAACAGTTCAACTGGATAGCG | Core staple |
| 06_cuboctahedron_147_8-6501-E | CGCACTCCAGGGCGCATCGTAACCGTGCATCCTCAGGAAGAT | Core staple |
| 06_cuboctahedron_147_8-6480-E | GCACCGCTTCTAGGTCACGTTGGTGTAGATGCCAGCTTTCCG | Core staple |
| 06_cuboctahedron_147_8-6470-E | TGGTGCCGGACGTAATGGGA | Core staple |
| 06_cuboctahedron_147_9-5304-V | TGTAGCAATACTTTTTTTCTTTGATTTGAAATGGATTTTTTTATTTACATTG | Core staple |
| 06_cuboctahedron_147_9-5419-E | GGAGGCCGATTTAGAGCCGTCAATAGATAATGGAGCTAAACA | Core staple |
| 06_cuboctahedron_147_9-5398-E | TAGACAGGAAGGAGCACTAACAACTAATAGATAAAGGGATTT | Core staple |
| 06_cuboctahedron_147_9-5377-E | AATCCTGAGAAGGTTATCTAAAATATCTTTACGGTACGCCAG | Core staple |
| 06_cuboctahedron_147_9-5356-E | AATCAGTGAGACAGTTGAAAGGAATTGAGGAAGTGTTTTTAT | Core staple |
| 06_cuboctahedron_147_9-5335-E | AAAGAGTCTGATCTGGTCAGTTGGCAAATCAGCCACCGAGTA | Core staple |

| | | |
|---|---|---|
| 06_cuboctahedron _147_9-5314-E | AATTAACCGTAATATCAAACCCTCAATCAATTCCATCACGCA | Core staple |
| 06_cuboctahedron _147_10-3099-V | AGCGCATTAGATTTTTCGGGAGAATTTAAGAAAAGTATTTTTAGCAGAT AGC | Core staple |
| 06_cuboctahedron _147_10-3214-E | GTTACAAAATCAGAATCAAGTTTGCCTTTAGCTAATTTGCCA | Core staple |
| 06_cuboctahedron _147_10-3193-E | TTATTTATCCCAGCACCGTAATCAGTAGCGAAAACAGCCATA | Core staple |
| 06_cuboctahedron _147_10-3172-E | AGAAACGATTCACCAATGAAACCATCGATAGCAATCCAAATA | Core staple |
| 06_cuboctahedron _147_10-3151-E | GTCAAAAATGCATTAGCAAGGCCGGAAACGTTTTTGTTTAAC | Core staple |
| 06_cuboctahedron _147_10-3130-E | CTTTACAGAGAATCACCAGTAGCACCATTACAAAATAGCAGC | Core staple |
| 06_cuboctahedron _147_10-3109-E | AAAACAGGGATTTGGGAATTAGAGCCAGCAAAGAATAACATA | Core staple |
| 06_cuboctahedron _147_11-2785-V | AAAAGAAACGCTTTTTAAAGACACCACACCGTCACCGTTTTTACTTGAG CCA | Core staple |
| 06_cuboctahedron _147_11-2845-E | TCCTTATTCAAAAGAACTGGCATGGCCAGCTG | Core staple |
| 06_cuboctahedron _147_11-6373-E | GCGAAAGGGCCTCTTCGCTATTACATTAAGAC | Core staple |
| 06_cuboctahedron _147_11-6428-E | GCGCAACTGTAAGTTGGGTAACGCCAGGGTTCCATTCAGGCT | Core staple |
| 06_cuboctahedron _147_11-6407-E | ATCGGTGCGGGGGATGTGCTGCAAGGCGATTTGGGAAGGGCG | Core staple |
| 06_cuboctahedron _147_11-2826-E | TAGCAAACGTACGCAATAATAACGGAATACCACGCAGTATGT | Core staple |
| 06_cuboctahedron _147_11-2805-E | ACATAAAGGTTACCAGAAGGAAACCGAGGAAAGAAAATACAT | Core staple |
| 06_cuboctahedron _147_11-2795-E | GGCAACATATCGAACAAAGT | Core staple |
| 06_cuboctahedron _147_12-1881-E | CCTCAGAACCTAGTAGTAGCATTTGTGTAGGAGTACCGCCAC | Core staple |
| 06_cuboctahedron _147_12-1860-E | AACCGCCACCAGGTGGCATCAATTCTACTAAGCCACCCTCAG | Core staple |
| 06_cuboctahedron _147_12-1839-E | CACCCTCATTCATTTGGGGCGCGAGCTGAAACTCAGAGCCAC | Core staple |
| 06_cuboctahedron _147_12-1818-E | CAAGCCCAATTAACCTGTTTAGCTATATTTTTTCAGGGATAG | Core staple |
| 06_cuboctahedron _147_12-1797-E | TACCGTAACAATACATTTCGCAAATGGTCAAAGGAACCCATG | Core staple |
| 06_cuboctahedron _147_12-1776-E | CACCAGTACATAGATTTAGTTTGACCATTAGCTGAGTTTCGT | Core staple |
| 06_cuboctahedron _147_13-139-V | ATTCCCAATTCTTTTTTGCGAACGAGAACTACAACGCTTTTTCTGTAGCA TT | Core staple |
| 06_cuboctahedron _147_13-832-E | CTTATGCGATTTTCATTCCATATAACAGTTGTTGTGAATTAC | Core staple |
| 06_cuboctahedron _147_13-811-E | GCTCATTATACTAAAGTACGGTGTCTGGAAGTTTAAGAACTG | Core staple |
| 06_cuboctahedron _147_13-790-E | GTTGGGAAGACAACATGTTTTAAATATGCAACCAGTCAGGAC | Core staple |
| 06_cuboctahedron _147_13-769-E | TAATAAAACGGCTGAATATAATGCTGTAGCTAAAATCTACGT | Core staple |
| 06_cuboctahedron _147_13-748-E | CAACATTATTCGGATGGCTTAGAGCTTAATTAACTAACGGAA | Core staple |
| 06_cuboctahedron _147_13-727-E | GATTCATCAGTTTGATAAGAGGTCATTTTTGACAGGTAGAAA | Core staple |
| 06_cuboctahedron _147_14-5723-E | CACTACGTGAAACAGAAATAAAGAAATTGCGGGGCGATGGCC | Core staple |
| 06_cuboctahedron _147_14-5702-E | AATCAAGTTTATCAAAATTATTTGCACGTAAACCATCACCCA | Core staple |
| 06_cuboctahedron _147_14-5681-E | GGTGCCGTAAGGAAGGGTTAGAACCTACCATTTTGGGGTCGA | Core staple |
| 06_cuboctahedron _147_14-5660-E | GGAACCCTAATGGATTATACTTCTGAATAATAGCACTAAATC | Core staple |
| 06_cuboctahedron _147_14-5639-E | GATTTAGAGCATCAATATAATCCTGATTGTTAGGGAGCCCCC | Core staple |
| 06_cuboctahedron _147_14-5618-E | AGCCGGCGAAATTATCAGATGATGGCAATTCTTGACGGGGAA | Core staple |

| | | |
|---|---|---|
| 06_cuboctahedron_147_15-4569-V | GGAATTATCATTTTTTCATATTCCTGCGTGGCGAGAATTTTTAGGAAGGGAA | Core staple |
| 06_cuboctahedron_147_15-4031-E | CTTTTTAAAAAATCATAGGTCTGATTTTAAAA | Core staple |
| 06_cuboctahedron_147_15-4619-E | GTTTGAGTGCCCGAACGTTATTAAGAGACTAC | Core staple |
| 06_cuboctahedron_147_15-4674-E | AAACAATTCGAAAGAAACCACCAGAAGGAGCTTAGACTTTAC | Core staple |
| 06_cuboctahedron_147_15-4653-E | TAAATCCTTTAACATTATCATTTTGCGGAACACAACTCGTAT | Core staple |
| 06_cuboctahedron_147_15-4012-E | GGTTGGGTTAAGAGTCAATAGTGAATTTATCCCTCCGGCTTA | Core staple |
| 06_cuboctahedron_147_15-3991-E | GTAAATGCTGGCTTAGATTAAGACGCTGAGATATAACTATAT | Core staple |
| 06_cuboctahedron_147_15-3981-E | ATGCAAATCCCATAGCGATA | Core staple |
| 06_cuboctahedron_147_16-3498-E | TATCATTCCATCATTAAAGCCAGAATGGAAACTGTCTTTCCT | Core staple |
| 06_cuboctahedron_147_16-3477-E | TAAACCAAGTTATTCACAAACAAATAAATCCAGAACGGGTAT | Core staple |
| 06_cuboctahedron_147_16-3456-E | CGAGAACAAGAGGTCAGACGATTGGCCTTGAACCGCACTCAT | Core staple |
| 06_cuboctahedron_147_16-3435-E | TATTTTCATCGCATTGACAGGAGGTTGAGGCCAAGCCGTTTT | Core staple |
| 06_cuboctahedron_147_16-3414-E | TACCGCGCCCCCACCACCAGAGCCGCCGCCAGTAGGAATCAT | Core staple |
| 06_cuboctahedron_147_16-3393-E | AATCAGATATCCCTCAGAGCCGCCACCAGAAAATAGCAAGCA | Core staple |
| 06_cuboctahedron_147_17-2344-V | CCGCCACCCTCTTTTTAGAGCCACCAAGAAGGCTTATTTTTTCCGGTATTCT | Core staple |
| 06_cuboctahedron_147_17-1365-E | AAAGACAGGCGGGATCGTCACCCTATCACCGG | Core staple |
| 06_cuboctahedron_147_17-2394-E | AACCAGAGTCTTTTCATAATCAAACAGCAGCG | Core staple |
| 06_cuboctahedron_147_17-2449-E | TCGGTCATAGTCAGAGCCGCCACCCTCAGAACATCGGCATTT | Core staple |
| 06_cuboctahedron_147_17-2428-E | GCGTTTGCCACCACCACCGGAACCGCCTCCCCCCCCTTATTA | Core staple |
| 06_cuboctahedron_147_17-1346-E | GGGTAGCAACAGGGAGTTAAAGGCCGCTTTTCATCGGAACGA | Core staple |
| 06_cuboctahedron_147_17-1325-E | CTTTGAGGACTATTCGGTCGCTGAGGCTTGCGGCTACAGAGG | Core staple |
| 06_cuboctahedron_147_17-1315-E | TAAAGACTTTATAACCGATA | Core staple |
| 06_cuboctahedron_147_18-6732-E | AGCTCATTTTTGCCAGCTGCATTAATGAATCTTTGTTAAATC | Core staple |
| 06_cuboctahedron_147_18-6711-E | GAACGCCATCTTCCAGTCGGGAAACCTGTCGTTAACCAATAG | Core staple |
| 06_cuboctahedron_147_18-6690-E | GCGTCTGGCCGCGTTGCGCTCACTGCCCGCTAAAAATAATTC | Core staple |
| 06_cuboctahedron_147_18-6669-E | AGCTTTCATCGTGAGCTAACTCACATTAATTTTCCTGTAGCC | Core staple |
| 06_cuboctahedron_147_18-6648-E | TGAGCGAGTAAAAGCCTGGGGTGCCTAATGAAACATTAAATG | Core staple |
| 06_cuboctahedron_147_18-6627-E | GATTCTCCGTCGAGCCGGAAGCATAAAGTGTACAACCCGTCG | Core staple |
| 06_cuboctahedron_147_19-6186-V | CTCACAATTCCTTTTTACACAACATAGGGAACAAACGTTTTTGCGGATTGAC | Core staple |
| 06_cuboctahedron_147_19-5207-E | CCAGCCATGTAATATCCAGAACAAACCGAGCT | Core staple |
| 06_cuboctahedron_147_19-6236-E | CGAATTCGTAGAGGATCCCCGGGTTATTACCG | Core staple |
| 06_cuboctahedron_147_19-6291-E | GTGCCAAGCTCCTGTGTGAAATTGTTATCCGAACGACGGCCA | Core staple |
| 06_cuboctahedron_147_19-6270-E | AGGTCGACTCTAATCATGGTCATAGCTGTTTTGCATGCCTGC | Core staple |
| 06_cuboctahedron_147_19-5188-E | AAACGCTCATCTCAAACTATCGGCCTTGCTGTGCAACAGGAA | Core staple |
| 06_cuboctahedron_147_19-5167-E | CATTTTGACGTCACTTGCCTGAGTAGAAGAAGGAAATACCTA | Core staple |

| | | |
|---|---|---|
| 06_cuboctahedron _147_19-5157-E | CTCAATCGTCAGTAATAACA | Core staple |
| 06_cuboctahedron _147_20-3246-V | TAACGAGCGTCTTTTTTTCCAGAGCCGTCAGACTGTTTTTAGCGCGT TTT | Core staple |
| 06_cuboctahedron _147_20-3737-E | ATATTTAAAGTAGGGCTTAATTGATCAAGATT | Core staple |
| 06_cuboctahedron _147_20-3296-E | AGTTGCTAGTTTTGAAGCCTTAAAGAATCGCC | Core staple |
| 06_cuboctahedron _147_20-3351-E | GCGTTTTAGCTATCCTGAATCTTACCAACGCAAGAACGCGAG | Core staple |
| 06_cuboctahedron _147_20-3330-E | CTTGCGGGAGTTTTGCACCCAGCTACAATTTGAACCTCCCGA | Core staple |
| 06_cuboctahedron _147_20-3718-E | TGTAATTTAGAGTATAAAGCCAACGCTCAACCAACGCCAACA | Core staple |
| 06_cuboctahedron _147_20-3697-E | TTCGAGCCAGTGCGTTATACAAATTCTTACCGCAGAGGCATT | Core staple |
| 06_cuboctahedron _147_20-3687-E | TAATAAGAGATAGTATCATA | Core staple |
| 06_cuboctahedron _147_21-3834-V | AATTACTAGAATTTTTAAAGCCTGTTATATAAAGTACTTTTTCGACAAA AGG | Core staple |
| 06_cuboctahedron _147_21-4913-E | GTATTAACGATAAAACAGAGGTGATTGAAATA | Core staple |
| 06_cuboctahedron _147_21-3884-E | CCGACCGTACCTAAATTTAATGGTGGCGGTCA | Core staple |
| 06_cuboctahedron _147_21-3939-E | TCAAATATATAAGAATAAACACCGGAATCATAGAAAACTTTT | Core staple |
| 06_cuboctahedron _147_21-3918-E | TCATCTTCTGGTGATAAATAAGGCGTTAAATTTTAGTTAATT | Core staple |
| 06_cuboctahedron _147_21-4894-E | CAGTGCCACGCCGAACGAACCACCAGCAGAAACCGCCTGCAA | Core staple |
| 06_cuboctahedron _147_21-4873-E | CAGCAAATGAAAAACATCGCCATTAAAAATACTGAGAGCCAG | Core staple |
| 06_cuboctahedron _147_21-4863-E | AAAATCTAAATGATAGCCCT | Core staple |
| 06_cuboctahedron _147_22-5010-V | ATTAGTCTTTATTTTTATGCGCGAACGCATCACCTTGTTTTTCTGAACCT CA | Core staple |
| 06_cuboctahedron _147_22-3002-E | GCCCAATAGATAACCCACAAGAATAACCCTTC | Core staple |
| 06_cuboctahedron _147_22-5060-E | TGACCTGATGGCCAACAGAGATAGTGAGTTAA | Core staple |
| 06_cuboctahedron _147_22-5115-E | AGTCACACGAAGACAATATTTTTGAATGGCTGCAGATTCACC | Core staple |
| 06_cuboctahedron _147_22-5094-E | AGGGACATTCAAGCGTAAGAATACGTGGCACCCAGTAATAAA | Core staple |
| 06_cuboctahedron _147_22-2983-E | AAACAATGAAATTGAGCGCTAATATCAGAGAATAAGAGCAAG | Core staple |
| 06_cuboctahedron _147_22-2962-E | TATCTTACCGCCTGAACAAAGTCAGAGGGTAATAGCAATAGC | Core staple |
| 06_cuboctahedron _147_22-2952-E | AAGCCCTTTTAACTGAACAC | Core staple |
| 06_cuboctahedron _147_23-580-V | CAAAAGAAGTTTTTTTTTGCCAGAGGTCAAAAATCAGTTTTTGTCTTTA CCC | Core staple |
| 06_cuboctahedron _147_23-1071-E | TAAGGGAAGAACGAGGCGCAGACGCTATCATA | Core staple |
| 06_cuboctahedron _147_23-630-E | ACCCTCGTCATAGTAAGAGCAACAGTCAATCA | Core staple |
| 06_cuboctahedron _147_23-685-E | CAGATACATACAAAATAGCGAGAGGCTTTTGTTCAACTAATG | Core staple |
| 06_cuboctahedron _147_23-664-E | AATTACGAGGTTACCAGACGACGATAAAAACACGCCAAAAGG | Core staple |
| 06_cuboctahedron _147_23-1052-E | AACTTTGAAACTGCTCCATGTTACTTAGCCGCCGAACTGACC | Core staple |
| 06_cuboctahedron _147_23-1031-E | AACGGTGTACAATTGTGTCGAAATCCGCGACGAGGACAGATG | Core staple |
| 06_cuboctahedron _147_23-1021-E | AGACCAGGCGTCGCCTGATA | Core staple |
| 06_cuboctahedron _147_24-1168-V | GTACAACGGAGTTTTTATTTGTATCACATAGGCTGGCTTTTTTGACCTT CAT | Core staple |
| 06_cuboctahedron _147_24-2688-E | CAACCGATCGCCAAAGACAAAAGGAGAATACA | Core staple |

| | | |
|---|---|---|
| 06_cuboctahedron _147_24-1218-E | CTAAAACAAAACGAAAGAGGCAAAGCGACATT | Core staple |
| 06_cuboctahedron _147_24-1273-E | TACGTAATGCTTATACCAAGCGCGAAACAAAAACGGGTAAAA | Core staple |
| 06_cuboctahedron _147_24-1252-E | CACCAACCTACTCATCTTTGACCCCCAGCGACACTACGAAGG | Core staple |
| 06_cuboctahedron _147_24-2669-E | AGGTAAATATAAAATTCATATGGTTTACCAGTGAGGGAGGGA | Core staple |
| 06_cuboctahedron _147_24-2648-E | ATTCATTAAATTATTTTGTCACAATCAATAGTGACGGAAATT | Core staple |
| 06_cuboctahedron _147_24-2638-E | GGTGAATTATCGGAATAAGT | Core staple |
| 06_cuboctahedron _147_5-4128-Verte x-3T-R1 | TTCCCTTAGAATTTTTTCCTTGAAAAAATCGCAAGACTTTTTAAAGAAC GCGTTTCCTCCTCCTCCTCCTCCT | Vertex 1a docking strand |
| 06_cuboctahedron _147_21-3834-Vert ex-3T-R1 | AATTACTAGAATTTTTAAAGCCTGTTATATAAAGTACTTTTTCGACAAA AGGTTTCCTCCTCCTCCTCCTCCT | Vertex 1b docking strand |
| 06_cuboctahedron _147_17-2344-Vert ex-3T-R1 | CCGCCACCCTCTTTTTAGAGCCACCAAGAAGGCTTATTTTTTCCGGTATT CTTTTCCTCCTCCTCCTCCTCCT | Vertex 2a docking strand |
| 06_cuboctahedron _147_20-3246-Vert ex-3T-R1 | TAACGAGCGTCTTTTTTTTCCAGAGCCGTCAGACTGTTTTTTAGCGCGT TTTTTTCCTCCTCCTCCTCCTCCT | Vertex 2b docking strand |
| 06_cuboctahedron _147_10-3099-Vert ex-3T-R1 | AGCGCATTAGATTTTTCGGGAGAATTTAAGAAAAGTATTTTTAGCAGAT AGCTTTCCTCCTCCTCCTCCTCCT | Vertex 3a docking strand |
| 06_cuboctahedron _147_11-2785-Vert ex-3T-R1 | AAAAGAAACGCTTTTTAAAGACACCACACCGTCACCGTTTTTACTTGAG CCATTTCCTCCTCCTCCTCCTCCT | Vertex 3b docking strand |
| 06_cuboctahedron _147_9-5304-Verte x-3T-R1 | TGTAGCAATACTTTTTTTCTTTGATTTGAAATGGATTTTTTTATTTACAT TGTTTCCTCCTCCTCCTCCTCCT | Vertex 4a docking strand |
| 06_cuboctahedron _147_22-5010-Vert ex-3T-R1 | ATTAGTCTTTATTTTTATGCGCGAACGCATCACCTTGTTTTTCTGAACCT CATTTCCTCCTCCTCCTCCTCCT | Vertex 4b docking strand |
| 06_cuboctahedron _147_1-1913-Verte x-3T-R1 | ATCACCGTACTTTTTTCAGGAGGTTTTAAAGATTCAATTTTTAAGGGTG AGATTTCCTCCTCCTCCTCCTCCT | Vertex 5a docking strand |
| 06_cuboctahedron _147_3-5755-Verte x-3T-R1 | AGGGCGAAAAATTTTTCCGTCTATCATAGATTTTCAGTTTTTGTTTAAC GTCTTTCCTCCTCCTCCTCCTCCT | Vertex 5b docking strand |
| 06_cuboctahedron _147_2-1609-Verte x-3T-R1 | ACTAAAGGAATTTTTTTGCGAATAATGTTTAATTTCATTTTTACTTTAAT CATTTCCTCCTCCTCCTCCTCCT | Vertex 6a docking strand |
| 06_cuboctahedron _147_13-139-Verte x-3T-R1 | ATTCCCAATTCTTTTTTGCGAACGAGAACTACAACGCTTTTTCTGTAGCA TTTTTCCTCCTCCTCCTCCTCCT | Vertex 6b docking strand |
| 06_cuboctahedron _147_7-286-Vertex-3T-R1 | AGAGTACCTTTTTTTTAATTGCTCCTTTGAGATTTAGTTTTTGAATACCA CATTTCCTCCTCCTCCTCCTCCT | Vertex 7a docking strand |
| 06_cuboctahedron _147_23-580-Verte x-3TR1 | CAAAAGAAGTTTTTTTTTGCCAGAGGTCAAAAATCAGTTTTTGTCTTTA CCCTTTCCTCCTCCTCCTCCTCCT | Vertex 7b docking strand |
| 06_cuboctahedron _147_4-5902-Verte x-3T-R1 | GCCCCAGCAGGTTTTTCGAAAATCCTGGAGCAAACAATTTTTGAGAATC GATTTTCCTCCTCCTCCTCCTCCT | Vertex 8a docking strand |
| 06_cuboctahedron _147_7-6764-Verte x-3T-R1 | TGTTAAAATTCTTTTTGCATTAAATTGGCCAACGCGCTTTTTGGGGAGA GGCTTTCCTCCTCCTCCTCCTCCT | Vertex 8b docking strand |
| 06_cuboctahedron _147_4-5451-Verte x-3T-R1 | TTCCTCGTTAGTTTTTAATCAGAGCGACATTTGAGGATTTTTTTAGAA GTATTTCCTCCTCCTCCTCCTCCT | Vertex 9a docking strand |
| 06_cuboctahedron _147_15-4569-Vert ex-3T-R1 | GGAATTATCATTTTTTCATATTCCTGCGTGGCGAGAATTTTTAGGAAGG GAATTTCCTCCTCCTCCTCCTCCT | Vertex 9b docking strand |
| 06_cuboctahedron _147_2-2060-Verte x-3T-R1 | ATTTCGGAACCTTTTTTATTATTCTGAGAAGATGATGTTTTTAAACAAA CATTTTCCTCCTCCTCCTCCTCCT | Vertex 10a docking strand |

| | | |
|---|---|---|
| 06__cuboctahedron__147_5-3530-Vertex-3T-R1 | TAGAAACCAATTTTTTCAATAATCGGGCGCAGTCTCTTTTTTGAATTTACCGTTTCCTCCTCCTCCTCCTCCT | Vertex 10b docking strand |
| 06__cuboctahedron__147_6-1462-Vertex-3T-R1 | GACAACAACCATTTTTTCGCCCACGCTTCATGAGGAATTTTTGTTTCCATTATTTCCTCCTCCTCCTCCTCCT | Vertex 11a docking strand |
| 06__cuboctahedron__147_24-1168-Vertex-3T-R1 | GTACAACGGAGTTTTTATTTGTATCACATAGGCTGGCTTTTTTGACCTTCATTTTCCTCCTCCTCCTCCTCCT | Vertex 11b docking strand |
| 06__cuboctahedron__147_8-6460-Vertex-3T-R1 | AACCAGGCAAATTTTTGCGCCATTCGTTCCCAGTCACTTTTTGACGTTGTAATTTCCTCCTCCTCCTCCTCCT | Vertex 12a docking strand |
| 06__cuboctahedron__147_19-6186-Vertex-3T-R1 | CTCACAATTCCTTTTTACACAACATAGGGAACAAACGTTTTTGCGGATTGACTTTCCTCCTCCTCCTCCTCCT | Vertex 12b docking strand |
| 06__cuboctahedron__147_21-3884-Edge-3T-R1 | CCGACCGTACCTAAATTTAATGGTGGCGGTCATTTCCTCCTCCTCCTCCTCCT | Edge docking strand |
| 06__cuboctahedron__147_20-3296-Edge-3T-R1 | AGTTGCTAGTTTTGAAGCCTTAAAGAATCGCCTTTCCTCCTCCTCCTCCTCCT | Edge docking strand |
| 06__cuboctahedron__147_10-3172-Edge-3T-R1 | AGAAACGATTCACCAATGAAACCATCGATAGCAATCCAAATATTTCCTCCTCCTCCTCCTCCT | Edge docking strand |
| 06__cuboctahedron__147_22-5060-Edge-3T-R1 | TGACCTGATGGCCAACAGAGATAGTGAGTTAATTTCCTCCTCCTCCTCCTCCT | Edge docking strand |
| 06__cuboctahedron__147_3-6974-Edge-3T-R1 | ATAAATTAATGTTGAGTGTTGTTCCAGTTTGCGTTCTAGCTGTTTCCTCCTCCTCCTCCTCCT | Edge docking strand |
| 06__cuboctahedron__147_12-1839-Edge-3T-R1 | CACCCTCATTCATTTGGGGCGCGAGCTGAAACTCAGAGCCACTTTCCTCCTCCTCCTCCTCCT | Edge docking strand |
| 06__cuboctahedron__147_13-790-Edge-3T-R1 | GTTGGGAAGACAACATGTTTTAAATATGCAACCAGTCAGGACTTTCCTCCTCCTCCTCCTCCT | Edge docking strand |
| 06__cuboctahedron__147_7-6814-Edge-3T-R1 | AAACAGGAGGTTGATAATCAGAAAATATCGCGTTTCCTCCTCCTCCTCCTCCT | Edge docking strand |
| biotin-06__cuboctahedron__147_21-3918-Edge | /5Biosg/TCATCTTCTGGTGATAAATAAGGCGTTAAATTTTAGTTAATT | Biotinylated staple |
| biotin-06__cuboctahedron__147_21-4894-Edge | /5Biosg/CAGTGCCACGCCGAACGAACCACCAGCAGAAACCGCCTGCAA | Biotinylated staple |
| biotin-06__cuboctahedron__147_22-5094-Edge | /5Biosg/AGGGACATTCAAGCGTAAGAATACGTGGCACCCAGTAATAAA | Biotinylated staple |
| biotin-06__cuboctahedron__147_22-2983-Edge | /5Biosg/AAACAATGAAATTGAGCGCTAATATCAGAGAATAAGAGCAAG | Biotinylated staple |
| biotin-06__cuboctahedron__147_10-3130-Edge | /5Biosg/CTTTACAGAGAATCACCAGTAGCACCATTACAAAATAGCAGC | Biotinylated staple |
| biotin-06__cuboctahedron__147_10-3193-Edge | /5Biosg/TTATTTATCCCAGCACCGTAATCAGTAGCGAAAACAGCCATA | Biotinylated staple |
| biotin-06__cuboctahedron__147_20-3330-Edge | /5Biosg/CTTGCGGGAGTTTTGCACCCAGCTACAATTTGAACCTCCCGA | Biotinylated staple |
| biotin-06__cuboctahedron__147_20-3697-Edge | /5Biosg/TTCGAGCCAGTGCGTTATACAAATTCTTACCGCAGAGGCATT | Biotinylated staple |

**Supplementary Table 2 3D wireframe cuboctahedron DNA origami strands.** Scaffold is MP13mp18. To make a pattern on a 3D wireframe cuboctahedron DNA origami, we mix scaffold strands, biotinylated strands, and core staple strands (positions corresponding to the pattern docking positions and biotin positions should be excluded beforehand) along with the corresponding docking strands that make up the pattern.

| Letters | Binary | Letters | Binary |
|---|---|---|---|
| A | 1000001 | T | 1010100 |
| B | 1000010 | U | 1010101 |
| C | 1000011 | V | 1010110 |
| D | 1000100 | W | 1010111 |
| E | 1000101 | X | 1011000 |
| F | 1000110 | Y | 1011001 |
| G | 1000111 | Z | 1011010 |
| H | 1001000 | Space | 100000 |
| I | 1001001 | 1 | 110001 |
| J | 1001010 | 2 | 110010 |
| K | 1001011 | 3 | 110011 |
| L | 1001100 | 4 | 110100 |
| M | 1001101 | 5 | 110101 |
| N | 1001110 | 6 | 110110 |
| O | 1001111 | 7 | 110111 |
| P | 1010000 | 8 | 111000 |
| Q | 1010001 | 9 | 111001 |
| R | 1010010 | 0 | 110000 |
| S | 1010011 | | |

**Supplementary Table 3 Letters to binary and number to binary.** In our demonstration, the last 6 digits of the binary encoding are assigned to the alphabet, while the last 4 digits are allocated to the numbers.

| Strands | Concentration |
|---|---|
| M13mp18 (for 2D RRO and Cuboctahedron) or P8064 (for Tunnel) | 20 nM |
| Core staple (positions corresponding to the pattern docking positions and biotin positions should be excluded beforehand) | 200 nM/strand |
| Biotinylated staple | 1000 nM/strand |
| Corresponding docking strands | 1250 nM/strand |
| TAE $MgCl_2$ buffer | 1× |

**Supplementary Table 4 Mixing concentrations for 2D RRO, cuboctahedron, and Tunnel origami experiments.**

| Imaging Parameters | NSF 2D dataset | 20 nm RRO 32 nt binder | 20 nm RRO 64 nt binder | 14 nm RRO 64 nt binder | 10 nm RRO 64 nt binder | ASU one redundancy 2D dataset | ASU two redundancy 2D dataset | 0407 3D dataset |
|---|---|---|---|---|---|---|---|---|
| DNA origami concentration | 1 nm, no fiduciary drift correction markers | 1 nm, no fiduciary drift correction markers | 1 nM, no fiduciary drift correction markers | 1 nM, no fiduciary drift correction markers | 1 nm, no fiduciary drift correction markers | 1 nm, no fiduciary drift correction markers | 1 nm, no fiduciary drift correction markers | 1.5 nM with 0.5 nM of 20 nm RRO for fiduciary drift correction markers |
| Imager concentration | 5 nM | 5 nM | 5 nM | 5 nM | 2 nM | 1 nM | 1 nM | 1 nM |
| PCA, PCD, Trolox concentration | 1.25X PCA, 1× PCD and 1× Trolox | 1.25X PCA, 1× PCD and 1× Trolox | 1.25X PCA, 1× PCD and 1× Trolox | 1.25X PCA, 1× PCD and 1× Trolox | 1.25X PCA, 1× PCD and 1× Trolox | 1.25X PCA, 1× PCD and 1× Trolox | 1.25X PCA, 1× PCD and 1× Trolox | 1.25X PCA, 1× PCD and 1× Trolox |
| Camera exposure time | 50 ms | 50 ms | 50 ms | 50 ms | 50 ms | 50 ms | 50 ms | 50 ms |
| Laser power density | 800 $W/cm^2$ | 800 $W/cm^2$ | 800 $W/cm^2$ | 800 $W/cm^2$ | 1250 $W/cm^2$ | 1250 $W/cm^2$ | 1250 $W/cm^2$ | 1250 $W/cm^2$ |
| No. of frames | 15,000 | 15,000 | 15,000 | 30,000 | 90,000 | 30,000 | 30,000 | 43,510 |
| TIRF | Yes | Yes | Yes | Yes | Yes | Yes | Yes | Yes |
| 3D lens | No | No | No | No | No | No | No | Yes |

**Supplementary Table 5 DNA-PAINT super-resolution imaging parameters for each experiment**

| Name | Sequence | **Note** |
|---|---|---|
| 5[32]25[31] | ATATCTATTATCTGGTCAGTTGGCTTATCTAATCTTTCCTTACCGCAC | Core staple |
| 12[55]28[32] | AGAAATAATAGATTTTATATTATTTATCCAGCGCATTAGA | Core staple |
| 12[183]29[191] | ATTCGCCAGCAACTGTCGCCACCCCACCCTCAGAGCCCAT | Core staple |
| 0[231]21[231] | TCTGGCCTAGCTTTCACAGGTCAGTACCTTTA | Core staple |
| 6[183]2[184] | AGTTTTAAGACGATAATCTGGTCACAACCAGCTTACGGCTATGCCGGG | Core staple |
| 22[95]3[79] | AAAAACAGCTTGATACCGATACTTAGCGGGTT | Core staple |
| 3[80]2[80] | GAGTGTTGTTCTCCGAGTGGTCAGTTTGGAAC | Core staple |
| 19[192]30[184] | ACAACATTGTTTCATTTGACAGGATTATTCTGAAAGCCAC | Core staple |
| 15[8]28[0] | ATATTCCCCAGAAGAGCTATCGCAAGAAACAATGAA | Core staple |
| 3[24]31[31] | GCCATTGCTGGATTATGAACGCGAAGGGCTTAGAACAAAG | Core staple |
| 6[231]24[208] | ATAGGTCACGTTGGTGGGAGCAAAGAGCGGAATCGTCAT | Core staple |
| 7[48]5[51] | AAATTAAACGCCACCCTCAATCAATAGTCTTTAATG | Core staple |
| 1[216]0[200] | TCTTAGCCTCCTGTTGCTCGTCATAAACATC | Core staple |
| 7[248]24[232] | GATTGTAATCAGAAAAGCTCAGGTCTTATTATAGTCACAGTT | Core staple |
| 10[235]9[252] | TTATAATCATATGTACCCCGGTTGA | Core staple |
| 6[247]3[250] | GCAAATATGCAAAGCGTTTTGTTATAAATTTTTGT | Core staple |
| 3[56]19[55] | ACCTTCTACCCTACTGCGGGATCTTACCAGTATAAAGAAAAAGC | Core staple |
| 23[28]22[5] | TAATATCCGGTATTCTCCCATCCTAATTTA | Core staple |
| 25[8]7[15] | GGGTATTACTAATAAGGAATT | Core staple |
| 2[79]23[71] | AAGAGACAGAGATAGAGACCTGAAAAATCAAGCTATTTTG | Core staple |
| 22[207]21[223] | GCGGATTACCAGCCGGGTCACTGTTGAGTAAGAGCGCCCTAAGAGAG | Core staple |
| 12[79]11[63] | ATTCATTTCAACATATCAAAGACACCACGGTCTTTCCAGTAACAAA | Core staple |
| 12[215]27[215] | AAAAAAGGGTGAGAATAGGATTAGCGGGTG | Core staple |
| 20[159]21[135] | CCAGTCAGGAGCTTGCCCTGACGAGAAGGCAGAAAGAAC | Core staple |
| 1[80]19[95] | TATTAAAGAACCGGTCGCAAGGTGTATTCGGT | Core staple |
| 22[250]21[250] | TGCATCAAAAAAAGCCCGAAAG | Core staple |
| 21[40]4[32] | AGCGAACCCAGATATAAAACGCTCTTTTGAATGGCCAGAA | Core staple |
| 9[136]24[144] | GGTTGGCCGTTCCGGCATTCCACATTTCGCCAAGTACGCT | Core staple |
| 30[79]0[80] | ACCAAAAGTACCCGACTTGAGCCACAACCATCAACCGATAGACTCCAA | Core staple |
| 17[96]15[95] | CGAGGGTATTCATCTTCTGACCTAACGCGAGA | Core staple |
| 26[159]12[144] | CACCCTCCACAGGCTTACCAGTCCCGGAA | Core staple |
| 10[31]27[23] | GAGGATTTAGAAGTATTTAAATCCAATTGAGCTGAGTTAA | Core staple |
| 28[215]12[184] | AGTACTCCTCAAGAGAAGCCACCAGCCGGATAGGCCGGAGACAGGCC | Core staple |
| 23[208]22[208] | TCATTGAATCCCCCTTAAGAGGTCATTTTT | Core staple |
| 28[103]13[103] | ACAAAGGGCGACATTCATGCTGATGCAAAAAC | Core staple |
| 16[191]14[160] | CAATGTGCTGCAAGGCGATTTCAGAGGTGGAGTGCCATCTCTCACCGG | Core staple |
| 13[136]12[120] | AACTGACCTTTGTGAGAGATAGACTTTCTCCG | Core staple |
| 22[183]18[184] | CAACACTAAATGCAGATACATAACGATTCATCGCCAGCATCCAAGGGT | Core staple |
| 25[192]9[207] | TGTTTAGATACCAGGCCAGAATTAATGCCGGA | Core staple |
| 15[80]18[80] | TTGCTAGAAATTTAATGGTTTGAACAGCAGCGAAAGACAGGGGAGTTA | Core staple |
| 27[96]10[96] | GACAAGCCTCTGTTATGTTGGCACGGAAAAATCGGTCTGAGAGACT | Core staple |
| 6[151]22[144] | TTTCCCGTTCAACTTTAATCATTTTATGCGATTGTAAA | Core staple |
| 24[55]7[47] | TCTTACGTTTTTATTTTCATCCTGAATAACCTCAAATATC | Core staple |
| 8[95]12[80] | TGACCTTTAATTAATTCATATGGTCGGCTTAGATAACTATATGGAATT | Core staple |
| 27[216]12[216] | CCTTGAGTAACAGGCTTAATCAACGCAAGGAT | Core staple |
| 8[63]10[56] | AGAGGTGTATTAACACCTACATTTAATGCCTGCAACA | Core staple |
| 7[16]4[5] | GAGGAAGGAAATCAACGAAACCAACCGTTGCCTGAGTAGAAG | Core staple |
| 25[64]24[80] | AGCGAATAAGTTTATTTTGTCACATTGCTTTC | Core staple |
| 10[183]27[191] | CGCTTCTGCCAGGCAAGCCGTCGAGAACCGCCTCCCTCAG | Core staple |
| 15[160]28[168] | CGGGAACGGATCAGCTTACGCAACTTTGCCACTCAGACAT | Core staple |
| 13[144]17[159] | AACTTTGATGAGTTTCCACCGTAACAGAATACCGGATATTCACGG | Core staple |
| 23[5]6[8] | CGAGCATGTAAGTTGAA | Core staple |
| 14[23]16[3] | AGAAACCATGATTATCGTACCGACAAAAGGTAAAGT | Core staple |
| 24[231]26[216] | CAGAAAACCAAGAGAAGTAATCGTAAATTTTGGCTATACTTAACGGGG | Core staple |
| 30[183]16[192] | CAGAACCAGTTGGGTAACGCCTATAACAGTTGCAAATGGT | Core staple |
| 14[159]15[159] | AAACAGGACAGATGAGACCAGGCGCATCCA | Core staple |
| 3[120]24[128] | GCGCCAGGGTGGTCGTGAGGCGAAGAATTATGTTCAACAG | Core staple |
| 19[144]19[127] | ATAAGCGTGTTTTCACGGTCATACCGGGATTGCCCTTCACAACACTCA | Core staple |
| 1[24]18[24] | TGGCAGATGAGTAAAAAATCGCCATATTTAACTGTAATTTAGGACAAC | Core staple |
| 12[31]29[23] | GGTTAGCCCGAACGTTATTTTGCGTAATAAGATTAGAGAG | Core staple |
| 24[127]9[135] | TTTCAGCGGTAAATGAATTTTCTGGAGCCACCAGTTGGGC | Core staple |
| 18[55]2[40] | AATCATAATTACAACAAACGCCTAGCCAACGCCACACGACGCTCAATC | Core staple |
| 27[32]10[32] | AACTGAACAATGGAAGTACCATATCAAAATTACTGAGAGCCAGCATTT | Core staple |
| 28[167]13[167] | AATCAAAATCACCACAAGAATCGGCGAAAC | Core staple |
| 9[80]8[64] | TTCATTTGTTTAATGGAAACAGAAGATAAAAC | Core staple |
| 27[112]14[120] | TTAGCCAGGGATAGCAACAACGCCAATCATAACGACCTGC | Core staple |
| 23[104]5[103] | GCCTTAGAAAGGAACGGGGAGAGGCGGTCCCTTATAAATTAGAATC | Core staple |
| 11[64]9[79] | AGAAGATGATGAAACAAAACAAAATTAACAAT | Core staple |
| 14[119]15[135] | TCCATGTTTATTTGTATCATCGCCTGATAAAT | Core staple |

| | | |
|---|---|---|
| 9[208]25[223] | GAGGGTAAGAGATCCGTCCAATACTGAAT | Core staple |
| 16[119]31[111] | GAGATTTTAGTTAATGCAACGGAATTATTAGCAAAA | Core staple |
| 19[168]3[167] | ACGTTAATTTTAGGAATGTCACTGAGCCAGCGGTGCCGGTGTGGTGCC | Core staple |
| 27[160]9[175] | ACCAGAGCCGCCATGTGGCCTTTAGTGGGAAAGTGCCATGTTTCGTCT | Core staple |
| 11[192]26[208] | TCACCATCAATATGATATTCGGGTCAGGTT | Core staple |
| 17[8]30[3] | TGTCCAGAAGCCCTTTTTA | Core staple |
| 0[39]21[39] | AGGCCACCTCACCAGTTCAACAGTGGCGTTTT | Core staple |
| 31[32]15[31] | TTACCAGAATGAAAATAGCAGCCTAATAACATAATATAAAAGATGATG | Core staple |
| 31[112]1[119] | TCACCAGTCACAGGAAGTTTCCATTTGCCCCAGCAGAG | Core staple |
| 9[48]25[63] | CTAGTCAGAGGCGAAGAGCCCAACGCTAACG | Core staple |
| 10[135]26[112] | ATCGACATGGATCAAACTTAAATTGAGACGCATTTGTAAC | Core staple |
| 28[191]15[183] | CTCAGAACTGGGAAGGCGGGCCTCTTCGCTATGGCGAAAG | Core staple |
| 3[168]5[175] | ATCCCACGGCAGCAACCGCAAGAAATGACTTGTAGAACGT | Core staple |
| 14[87]28[72] | CAATTGAATACCAAGTCTTATTACAGCAAACG | Core staple |
| 11[0]10[2] | ATTCGACAACTCGTATAGACTTTACAAA | Core staple |
| 30[95]14[88] | GTGAATTATTGAGGGAGGGAAGGTCGGTCCAATCGCAAGA | Core staple |
| 6[207]4[208] | GCGCATCGGCACTCAATCCGCCGGGCAACGGGAACAGCGGTTGCGG | Core staple |
| 13[104]9[103] | GAGGCGCGGATAGCCTCATAGGATCTAAAGTTTATTTATCAAAA | Core staple |
| 13[216]14[224] | TTTTAGAACCCTCGCAAAATTAAGCAATAGCAAGGCAAAGAATTAATAT A | Core staple |
| 1[96]17[95] | CACGCTGGTTAAACGGGTAAACAATTTGGGAAGGCTTGCACATCGGAA | Core staple |
| 27[0]12[0] | ATAACCCATTAAAAGT | Core staple |
| 10[215]10[184] | TGCAACCGTTCTAGCTGATACTTTCCGGCAC | Core staple |
| 12[244]27[236] | AAGCCTCAGAGCATTTGA | Core staple |
| 17[64]14[56] | TCACCCTATACCGACAAGACTCTACCAGATGAATATA | Core staple |
| 19[56]30[64] | CTGTTTAGTATCATATTTTGCGTAACGGAAAACTGGC | Core staple |
| 6[95]4[72] | GCTATATGTGAGTGAAAATTTCTTATAGCCCGAGATAGTA | Core staple |
| 29[0]13[7] | ATAGCAATGAGCGGAATTGAGTAA | Core staple |
| 3[5]3[23] | TAGTAATAACATCACCCA | Core staple |
| 27[237]29[244] | TGATACAGGTAAGTTCCA | Core staple |
| 29[192]13[215] | GAAAGTATTGTCGGTGGCGATGTAGGTAAAGATTCAAAT | Core staple |
| 17[24]14[24] | AATAACAGAGGCATTTAATAAGAGAAAAACAGTAATCCTGATTGTCAA | Core staple |
| 6[127]8[120] | TTTCCAGTAATGAGTGAGCTAACTGAGCCGGAAGCATAAA | Core staple |
| 18[111]0[96] | AGGCTTTGACTTTTTCATGTAACGCCTGGCCCTGAGAGAGTTGCA | Core staple |
| 5[152]4[136] | CGTAATCTGCCAACGGCCACAGTTGAGGAT | Core staple |
| 15[184]16[208] | GGGGATAACCTGTTTAGCTATATTTTCATTTATTAGAT | Core staple |
| 23[72]24[56] | CACCCGTGTAACCTTGCTTCTCCTAAAACATAATACCGTCCTGAA | Core staple |
| 31[240]0[232] | ACAAATAATCAAATATTTCGAGCTTCAAAAATAATTCGCG | Core staple |
| 6[159]26[160] | ATAGCTGGAAATTGGAGGTTTCCCTCAGAACAGTATATATACGC | Core staple |
| 16[239]29[231] | AACATCCAAGGTGTTAGTCTCTGA | Core staple |
| 5[52]3[55] | CGCGAACTGATTGGCACAGACAATATATGGAAAT | Core staple |
| 30[207]17[223] | GGAACCTAGGTTGAGGCAGGTCAGACGATTGCAACTAAAAACGAGTA | Core staple |
| 10[55]8[40] | GTGCCACGTTTGCACGAGCCTAATTTGCCCTGAACAAGCACATCACCT | Core staple |
| 22[143]21[159] | TTGGGCTTCCGTGAGCCTCCTAGCACCGTCGGCCCCCTAGAACTG | Core staple |
| 5[104]6[128] | CTTGAGTCGTGCCAGCTGCATTAATGAACGGCTGCCCGC | Core staple |
| 1[5]2[5] | AATTAACCGTCTTCTTTGAT | Core staple |
| 19[208]30[224] | GCTCAACATGTTTTAAATGCAAACGGAAATGGCCTTGATGAATGGAA | Core staple |
| 21[5]1[23] | ACAAGAAAAATAATATAATTACAATATGTAGCAT | Core staple |
| 4[135]22[120] | CCCTCGGCCAACGCGCAACTAAAGTAATAATT | Core staple |
| 0[167]22[168] | AGAATGCGCAGCGCAGTACTTATAGCTCACACATTCAACTTCATAACC | Core staple |
| 27[56]12[56] | ACAACATAAAGGTGGCAATTACCTGAGAAC | Core staple |
| 23[56]1[55] | AATGTTGATTAAGCAAGCAAATTCCCGACTATTTTGACCAGTAATA | Core staple |
| 3[233]5[237] | GGCGCATAAATTATTCTCCGTGGCGGATTGACCGTAA | Core staple |
| 10[159]27[159] | AGTTAAACGATGCTGAAAAGCCGAGAACCGCATGTACCGTAACGGA | Core staple |
| 24[183]6[160] | GAGGGGGTGTATCACCTACCAGACCGGAACGTGCCGGGTC | Core staple |
| 30[119]29[135] | CCGGAGGACTAATACCAAGCGCGAAACAATCTAGAGTAAAAAACCATC | Core staple |
| 2[103]3[119] | GTTTGATGAATCGGCAAAATTTGCGTATTGG | Core staple |
| 18[79]21[79] | AAGGCCGCTGCCGCATGCCCAGTTATACAAATGGTTTTGAAGCGTTGC | Core staple |
| 15[32]29[55] | GCAATTCATCATTTTAGTACCATAGGACAATCCAAATAAG | Core staple |
| 4[207]6[184] | TATGAGCATCAGCGGGGCGCTTTCTAACCGTGCATCTGCC | Core staple |
| 25[96]8[96] | AAATGTCGTCTTTCCCCGAAGAGTCAATAG | Core staple |
| 25[224]10[216] | GACCATAAATAAGTTTAGCATGTCGACCCTGTAATACTTT | Core staple |
| 0[199]2[200] | CCTTACACAGCAAATCGTTTGGGTGGTAAAAC | Core staple |
| 8[39]11[31] | TGCTGATCTTTAGGAGTAGATAATCAGAGGGTTTTGAACC | Core staple |
| 30[23]17[7] | TTACACCGACGACGACATGTTCAGCTAATGCAGAACAATTC | Core staple |
| 17[224]31[239] | GATGCATCAATTCTACTAAAGCCAATTCACAA | Core staple |
| 25[32]9[47] | TCATCGAGAACAAAGTACAGCAAATGAAAAAT | Core staple |
| 11[216]10[236] | AGAAGCCTTTATTAGCTAAATCGGAACA | Core staple |
| 13[40]27[55] | CTGTTGCGAGAAAAATACCAGTTACAAAATAA | Core staple |
| 21[200]1[215] | GAATGGCTTAGAGCTTGCGGCTAAAGGTT | Core staple |

| | | |
|---|---|---|
| 26[207]6[208] | TTGCTCAGCTGGATAGTACAAAGGATTGCCTGAGAGTCTTAGATGG | Core staple |
| 12[119]16[120] | TGGTGAAGAGACGGTCTGTAGCATGACAACGTCACCAATGGTACAACG | Core staple |
| 16[207]19[207] | ACATTTCGATTCCCAATTCTGCGGTACGGTGTCTGGAAATTCTGTA | Core staple |
| 21[136]2[128] | ACCAGAACGAGTATTAGCAGCGTGCCTGTTCTTCGTTTTC | Core staple |
| 28[71]13[71] | TAGAAAATACATGCCCAGGTTTAACGTAAA | Core staple |
| 24[143]5[151] | AAACAACTGTTTAATTTGCGCTCAGTACCGAGCTCGAATT | Core staple |
| 13[72]30[80] | ATCGCGCAGAGGCTAAAACATGTTGCAGTCGATCACCGTC | Core staple |
| 24[79]7[79] | GAGAGCTACAATTTTAAACGAACCACCAGCAG | Core staple |
| 31[176]0[168] | GAGCCGCCAGTTGAGAAAAACGAACTGTGGTGCTGCGGCC | Core staple |
| 27[136]7[151] | AGGAACCCCACCCTCATATGGGATCAACATACCACATTAATTGTGTGT | Core staple |
| 14[55]16[40] | CAGTAACACATCGGGATAAATAAGGCGCCAGT | Core staple |
| 22[119]2[104] | TTTTCACGGGCACCAAAGTGGCGAAAATCCT | Core staple |
| 13[8]26[0] | CATTATCATTAATTCAAGAATGCTAATATCAGAGAG | Core staple |
| 29[56]15[79] | AAACGATTCGTGTGAGAAACAATAACGGATTCGCCTGA | Core staple |
| 0[79]1[79] | CGTCAAAGGGCGAAAACATTCTGGCCATCCAC | Core staple |
| 13[168]25[183] | GTAGCTGCTTCAGCAGCACCACCGGAGGGTTGAGCCCGGAATAGGTAA | Core staple |
| 30[63]17[63] | ATGATTTTTTGTTTAAAATAAGAATAAACTCG | Core staple |
| 8[159]10[160] | GCTCACAAAACGCGGTCCGTTTAAGGGTAA | Core staple |
| 2[183]19[191] | TTACCTGCCGCGCCTGTGCTGTTCTGGTGACTCTAACGGA | Core staple |
| 29[136]27[135] | GATAGCAGGTCACCAGTACAAACTAGCCCAAT | Core staple |
| 4[71]5[87] | AGAATACGAGCGTAAATCGTCGCTATTAATTA | Core staple |
| 19[128]18[112] | TCTGCTCAAAGCTTTGACCCCCAGCGATTCAG | Core staple |
| 30[223]15[232] | AGCCCTGCTGCCCGCAGTTTGACCGGGGCGCGAGCTGAAAA | Core staple |
| 21[80]22[96] | GCCGACAATGAATACGTAATGCCACTACGAATTGAAAATCTCCAAA | Core staple |
| 22[167]7[175] | CTCTTACCGTGAAGTTGTACTCAGTTACCAGAGCACATCC | Core staple |
| 15[96]28[104] | AAACTTTTTCAAATAACTTAGCAAATATTTCCACAG | Core staple |
| 30[159]16[144] | AGCTAGCGATCAGGTTCCGAGGCTGGCTGAC | Core staple |
| 14[223]30[208] | TTTTAAATGCAATGCCTGAGTAATAAGAGGCTGAGTAACTATTTC | Core staple |
| 20[223]21[199] | GATTATTGCTGAATATAATGACAGGTAGAAAGCCAAAAG | Core staple |
| 5[176]22[184] | CAGCGTGGTGCTGCAGGTCATTGGAAACCAAAAGTAAGAG | Core staple |
| 8[191]8[160] | CGGCCTCAGGAAGCGCTGGCAGCCTCCGGTCC | Core staple |
| 7[80]25[95] | TACATAAATCAATTAGTTATCAGCATCAATAG | Core staple |
| 1[56]0[40] | AAAGGGAACCGTCTATCATTATAATCAGTG | Core staple |
| 31[3]20[5] | AGAAAAGTAAGCAGATAGCCATTATAGATAAGTCCTGA | Core staple |
| 29[232]28[216] | ATTTACCGCGTCATACATGTGCCCGTATAAAC | Core staple |
| 9[120]10[136] | CCGCCAGCACCCTCATGAAACAGCAAAAAAATCCCGTAAAATTTGTAC | Core staple |
| 7[176]25[191] | TCAGAGGGGACGACGATTTTGCCATAGTAAAA | Core staple |
| 15[136]13[135] | TGTGTACAACGGTGTCGAAATCCGGGGAACCG | Core staple |
| 21[232]6[232] | ATTAGAGGGATTAGCTCCTTTTGACAAATGCTTTAAAGAATTAATGGG | Core staple |
| 27[24]13[39] | GCCGAATTCGGGACAAGAATTGGATTATACTT | Core staple |
| 30[247]18[240] | ATCCTCATTAATAGTATCAAAGCG | Core staple |
| 17[160]31[175] | CCAGTGCCAAGCTTAACCATAGCCGGTCACCA | Core staple |
| 19[3]0[8] | GCGCCTGTTTATCAACAGAGGAGTCTGTCCATCAC | Core staple |
| 5[88]23[103] | ATTTTCCCTCAAAAGAAAAGGCTCCAAAAGGA | Core staple |
| 2[199]23[207] | GGCATCAGGGAGGTGTCGAGGCATATAGCGAGAGGCTTAT | Core staple |
| 16[143]30[120] | CTTCATCATGACAAGACAAGTTTGCCTTTCATTAGCAAGG | Core staple |
| 7[112]6[96] | GGGTGCCTCGGGAAACCTAAACATAGCGATA | Core staple |
| 9[16]24[8] | GCCGTCAACACTAACAAAACCAAGTATCATTCCAAGAAC | Core staple |
| 2[39]23[55] | GTCTGAAAAACAGGAAGAAGGCTTCGGGTAGGAATCATTACCGCGCCC | Core staple |
| 17[40]18[56] | TCGAGTTACGTCAAAAAGGAAACCGAGGAAACGCAATAAGGAACCGG | Core staple |
| 24[111]7[111] | ATTAATTGTATCGGTATTAAGACGCTGATG | Core staple |
| 1[120]1[151] | ACGGGCAACAGCTGGGTTTCTGCCAGCACTCA | Core staple |
| 5[13]23[27] | ACTATCGGCCTTGCTGGTACAATATTATCAA | Core staple |
| 9[176]11[191] | CGTATCGCACTCCAGCGGATAAGTAGCTCAAA | Core staple |
| 19[96]30[96] | CGCCTAAAGAGGATGATTAGAGCCCATTAAAG | Core staple |
| 26[239]7[247] | TACTGGTAATCAAAAACCCGAACGTCGATAAAAACAGGAA | Core staple |
| 9[104]24[112] | TCATATGCTCATTTGGTGTAAAGAGACGTTAGAGTGAGA | Core staple |
| 2[127]31[143] | TTTTCACCCCTAAAACAAAGAATAAGCACCATTACAGCGTCAGACTGT | Core staple |
| 24[207]8[192] | AAATTTGCAAAAGAAGCAGAGGTCCTATCTAT | Core staple |
| 10[95]27[95] | ACCTTTTTAACCAAGAAACATCTCTTAAAACGAAAAGCCAGCGCCAAA | Core staple |
| 18[183]30[160] | TTTCCCAGTCACGACGTTGTAAAAGCATTTTCCCCTTATT | Core staple |
| 2[250]1[250] | TAAATCAGCTCCAATAGGAACG | Core staple |
| 18[239]3[232] | AACCAGACCTCTTTAACGCGTCAATCATTAACATTTTACATTAAATGTCAACCCGTC | Core staple |
| 1[152]19[167] | GACGATCGCGGGCCTGGGAAGAAAAATCT | Core staple |
| 31[144]19[143] | AGCGCGTTTTCATCGCGATTACCCAAATCAACGTAACATTCAGTGA | Core staple |
| 13[8]26[0] | CATTATCATTAATTCAAGAATGCTAATATCAGAGAGT/3Bio/ | Biotinylated staple |
| 17[8]30[3] | TGTCCAGAAGCCCTTTTTAT/3Bio/ | Biotinylated staple |
| 31[3]20[5] | AGAAAAGTAAGCAGATAGCCATTATAGATAAGTCCTGAT/3Bio/ | Biotinylated staple |
| 23[28]22[5] | TAATATCCGGTATTCTCCCATCCTAATTTAT/3Bio/ | Biotinylated staple |

| | | |
|---|---|---|
| 9[16]24[8] | GCCGTCAACACTAACAAAACCAAGTATCATTCCAAGAACT/3Bio/ | Biotinylated staple |
| 15[8]28[0] | TCCTCCTCCTCCTCCTCCTTATATTCCCCAGAAGAGCTATCGCAAGAAAC AATGAAT/3Bio/ | Biotinylated staple and docking |
| 30[223]15[232] | AGCCCTGCTGCCCGCAGTTTGACCGGGGCGCGAGCTGAAAATTCCTCCT CCTCCTCCTCCT | docking |

**Supplementary Table 6 DNA origami tunnel strands.** Scaffold is p8064. Core staples are all the strands that form the structure. Biotinylated staples are modified with biotin to immobilize origami on coverslip surfaces through a BSA-Biotin-Streptavidin-Biotin-DNA origami arrangement. To form DNA origami tunnel for DNA-PAINT experiment, we mix scaffold strands, biotinylated strands, and core staple strands (strands at positions corresponding to the docking positions and biotin positions should be excluded beforehand) along with the corresponding docking strands.

| Name | Sequence | **Note** |
|---|---|---|
| Tetrapod_10[118] | GATAAAAATTAGCAATAGCTAT | Structure |
| Tetrapod_10[65] | GAGTTTATAAATTAGCAAGGGAGGCCGATTATCCTGAGAGAACTCAATA | Structure |
| Tetrapod_11[126] | CTTACCGAATAACCACCAGCAGAA | Structure |
| Tetrapod_11[137] | TTTAATAATACAAAAATGAAAATA | Structure |
| Tetrapod_13[119] | TTTTTGTTTAACGTAGAGCAAAAATGGAGCCCT | Structure |
| Tetrapod_13[147] | GCAGCCTATTGAGTGGCAGATCTTTAATGCGCGAACGCAAA | Structure |
| Tetrapod_13[54] | CTCGTAATTTGCCAGTTCAATAGCCATTGCCTTG | Structure |
| Tetrapod_13[75] | AATAAACAGCCATATTACAACTATCATGGTTTGAT | Structure |
| Tetrapod_15[143] | ATTTAAGCCCTTAAGAAAAGTAAGCA | Structure |
| Tetrapod_15[77] | AAAACGCATAGATTAGTTGGCAAATCAACCACCGCCTGAGTAAAATCA | Structure |
| Tetrapod_16[83] | ACGCTAATCATATTCCTGATTTGAATAAC | Structure |
| Tetrapod_17[144] | TCAATCAAGGAAACAATA | Structure |
| Tetrapod_18[72] | AAAATTATCACGAGCGTCTTTCCAGAGCCTACCAGAAGTATAATCCAGA | Structure |
| Tetrapod_18[83] | CTTGCTTAGACTACCTTTTTCC | Structure |
| Tetrapod_19[91] | GACAAAAGTATGTGAGTAATGGACCTGAATCTTACCA | Structure |
| Tetrapod_22[153] | AACAAACTTACCGCGCCCAATAGCAAGCAAAGAAGTGATTGC | Structure |
| Tetrapod_25[136] | CAAACAGCTTAGTTTCAGCGGAAACATTGAATGAGTTAGAGTCT | Structure |
| Tetrapod_27[126] | TTTATAAAATATCGCCCACGCATAACCGATAGCACATTGCAAGGAG | Structure |
| Tetrapod_27[136] | TATCCTGATACGAGTGAGATCGGTTTTGTAAATTACATAGTGAGAATGA<br>A | Structure |
| Tetrapod_29[119] | TTCGCATCAAAGGACGTTTAGTTCAACGATACC | Structure |
| Tetrapod_29[56] | AGGTTGGAGGTCAAACTAAAATGTCGTCGTACAAA | Structure |
| Tetrapod_29[77] | AGGATGCAGGTGAGACGAAAGTAAATGAACACT | Structure |
| Tetrapod_3[46] | AAGGCTTTGATCAGAGCGGGAGCTAAACAGCCGCGTACTGAT | Structure |
| Tetrapod_3[94] | CAGCATTGACAGGATTTAAAGGGAGCCCCCGATTTAG | Structure |
| Tetrapod_30[149] | TTTCTTAAATCCCCATTCTGCAATGTGCGATTCGAGGTAGAAAG | Structure |
| Tetrapod_31[77] | ACGTTAGAGGCAAATCATGAGGAAGTTTCGAGTTAAAACCA | Structure |
| Tetrapod_32[69] | CGCTATTACGGGATCGGTCAAACAGACCAACAAAG | Structure |
| Tetrapod_33[144] | TGTCTAACGGGTTTAATT | Structure |
| Tetrapod_37[125] | CGCGTTTTAATTCGAAATATAATGAGATGAACAA | Structure |
| Tetrapod_37[91] | AAGATTAAGTACGGCTGACGAGATTTAGTTGAAAGAGGAAATAAGG | Structure |
| Tetrapod_38[153] | AAACGAAACCAAAAACATTATGACCCTGAATCTACCATTGTG | Structure |
| Tetrapod_39[84] | CTTGCCTGCCAATACTGCGGCGGATTGCATCAAA | Structure |
| Tetrapod_41[151] | TTCTCCGATATCATTGATTC | Structure |
| Tetrapod_41[161] | CAGCTTTCATCAACTCATTGTTAGAAGA | Structure |
| Tetrapod_44[80] | ATACATTCGCATTACGCCAGC | Structure |
| Tetrapod_46[185] | ATATCTGCCACATTA | Structure |
| Tetrapod_47[165] | AAATGTGAGTGTGGCGACTGTAGC | Structure |
| Tetrapod_47[45] | TTGTAAAACGAAACGAGG | Structure |
| Tetrapod_47[70] | CTTTATTATCAAACGCCGCGACCAGGAGCGGCCAGGGATGTG | Structure |
| Tetrapod_9[137] | GAAACTGATATTATTTAC | Structure |
| Tetrapod_9[147] | GACACCACGGAATATATTAGTTCACCAG | Structure |
| Tetrapod_0[108] | TTATGGAACCTTTTGGGCCACGCTGGTTTGCACCCTCGCAGGTC | Structure |
| Tetrapod_0[129] | GCTGTGAAAGTAAAAACCAGGCGAAAATCAACCGCCTATTCACA | Structure |
| Tetrapod_5[101] | TAATCCACCAGAGCGGTGTCGAGGTGCCG | Structure |
| Tetrapod_5[112] | GACAGAATCAAGTTGAGCCACCCCCAGCGTCTA | Structure |
| Tetrapod_5[132] | TAGCGTCAGACTGTACCCTCAGCTGTTT | Structure |
| Tetrapod_6[146] | CGTCAAAGGGCGATTAAGA | Structure |
| Tetrapod_8[104] | TTAGAGCCAGCAAAAGAGTCTTACTTCAAATAC | Structure |
| Tetrapod_13[108] | TAAGAAAATATTTTGACCCGTTGTAGCAAGTCCATCAGGCGGTC | Structure |
| Tetrapod_16[111] | AGCTACAATTTTATAACAGTATTTGAAAGATGA | Structure |
| Tetrapod_16[122] | ATTTGCCGTTTATTACAGTACCTTTTACATAGGCAGAACAACAT | Structure |
| Tetrapod_21[94] | CCGTTATAAAGAACGTCTTACCTTTTTATCAGATGAGATTTTC | Structure |
| Tetrapod_21[114] | TAAATTTCGAGCAGTAACATTTAACA | Structure |
| Tetrapod_21[125] | AAACACCGGAATCATTGTAATTTCGGGA | Structure |
| Tetrapod_22[139] | AAAACAAAATTATTATTTT | Structure |
| Tetrapod_23[105] | ATATACCAGTAATATTCTGTCCAGACGATACCGCACTCATCTA | Structure |
| Tetrapod_24[104] | TTTTATGGAGATGATCATTTTTACCGTAATTTT | Structure |
| Tetrapod_29[108] | TGACCTACGAAGGGATTTAGGAACCCATGCAGGGATCGCTGAGG | Structure |
| Tetrapod_32[90] | ACTATCGACATCATAACTGACCAACTGAAAAAGGAATTACGAGGACTGG<br>A | Structure |
| Tetrapod_32[111] | ATCGAACAAGACCCATTCAACCAGTTGAGAAAC | Structure |
| Tetrapod_32[122] | TGCGCCTCAGATACAGGGTAAATTGGGCTTGCTGTAGCGAACGA | Structure |
| Tetrapod_37[105] | GCCCGAAAGACTTCATGTTTTACGAGTATAGAAAG | Structure |
| Tetrapod_39[105] | ACCAGAAAATATGTATAACAGTTGATTCGAATTAG | Structure |
| Tetrapod_40[96] | ATCGGCCTCAGGAGCGGAGGATCTGGAAAATTAACTCTCAGGCACAGAT<br>CT | Structure |
| Tetrapod_40[117] | GTTTGAGGGGACGAGTAAACGTCGGAAACCAA | Structure |
| Tetrapod_40[139] | CATCGTAACCGTGCCATCAAACGCCTGAATGAA | Structure |

| | | |
|---|---|---|
| Tetrapod_45[101] | GGGAGAGGGTACGTATTCTTGTGCAAGTTGCCATATTCACC | Structure |
| Tetrapod_45[112] | TATTGCTAAACTGGAGCTGATGGGATTGTCGTT | Structure |
| Tetrapod_45[133] | CGAAGTCCGTGAAGAATATGACAGACAGATGAA | Structure |
| Tetrapod_47[112] | CAGTTCAAAATTAAGAGAGTCTGGAGCAACTCATTTT | Structure |
| Tetrapod_1[164] | TTAGCCCCGGGTACCGAGAGTCACACCACCCTGTCACCATTTCCAG | Structure |
| Tetrapod_1[186] | CAGATATAAGTATAGCCGTATCACGAACCGCGCCTGTAACGA | Structure |
| Tetrapod_14[65] | CCTGAGTAGAAAGTGTTCCAGCAGGTCACGCGAA | Structure |
| Tetrapod_14[83] | TAGTAATGGCCACCGCAACAGACACCCGCCAGCCGCCGC | Structure |
| Tetrapod_15[53] | TTACCGCCAGATAATACACCCTCATATTAATTATCAAAAACT | Structure |
| Tetrapod_18[52] | TTTTGAAACCATTAAATCCTTTGCCACAATTCGATT | Structure |
| Tetrapod_2[59] | CGTGTCCAGTCTGTAAAGCCTGGGGGTTAATTTGTAAATCGT | Structure |
| Tetrapod_2[83] | AGCTTGAGCCAGCTATACGAGCCGGAAGAAATTTAAGGT | Structure |
| Tetrapod_20[58] | ATATCATCTTCTGACCTCATAAAGGGGAAACACGCGCGCCTTC | Structure |
| Tetrapod_20[72] | TGGTTGCGTATGGCAATTCATCAAGAGCGGATCGTCGCATCAATACTGA | Structure |
| Tetrapod_21[39] | ATATATTTTATGCCTAACTCACTGGCGTATTAGAC | Structure |
| Tetrapod_23[39] | ATTATTTGCAGCTGATGGTCAATAAGGAAGGTGGC | Structure |
| Tetrapod_23[52] | AAAACTGATTGTTTGGATGAACAAACCCTTAGACCTCAAAAAA | Structure |
| Tetrapod_23[60] | AATAAAGAAAGTTATATATCATAGAGCCGGCTGCGCGTTACA | Structure |
| Tetrapod_25[66] | ACCGGGAGAATGGTAGGTCGGAAATGATTAGCGTCCACTAAAATCC | Structure |
| Tetrapod_25[88] | CCCTAAATGCACTTCCGCTCGAATACTCCTCACTCCAAGATGGTG | Structure |
| Tetrapod_30[62] | CTACAACCACCCTCACCCTCAGTACTGGGGAAAGCACCG | Structure |
| Tetrapod_30[83] | GAGTTTCCAGAGCCGGCCGCTGGGGTCAAATCCTCGCC | Structure |
| Tetrapod_31[53] | TCTAAAGTTTCACTCATCTTTGAGACACTATGGGGTAATTC | Structure |
| Tetrapod_32[51] | ATCATCGCCTACAAAGTCCCC | Structure |
| Tetrapod_36[38] | TTAAAAAATCAGGTCTTTTTATTAGGAACCAGATCAAAAAGTTTGG | Structure |
| Tetrapod_36[59] | ATTGCTATTATAGTCAGAGCATTTTCCGCCTCAATCGGCATTAAAG | Structure |
| Tetrapod_38[52] | CGCATAGGCTGGAACGAGCTCGTTTAACGGCTAAGA | Structure |
| Tetrapod_38[72] | TGAACGGTGTTCATAAGAAGAGCAGACTAAAGATCGTCAGA | Structure |
| Tetrapod_39[42] | ACCCAAACTCAAATAAGTTTTCTGAATTGATGATATGCT | Structure |
| Tetrapod_39[63] | CTGCTCAATAAATATAGTAAACCAGAATTAATAAGGGA | Structure |
| Tetrapod_4[150] | GCCAGCGCGTTTTCATCGAGCAAAGAATCGTCTTCAGTGCAGA | Structure |
| Tetrapod_4[170] | GAACGGTCATAGCCCCCACCCTGAAATCCCCTCAACGTAGG | Structure |
| Tetrapod_4[65] | GGGCGGAAACGTCACCACATTACCTCAGTGAAACATCAAACAGGA | Structure |
| Tetrapod_4[79] | AATCCCTGAGTAATGAATCGGCCACTGTCGTCGGGGAAGTCTGAGCTGT | Structure |
| Tetrapod_6[160] | AACGTGGAAGAGAATTTTAACTTTGCGGGACTTTTAGAATACTGGGTTC | Structure |
| Tetrapod_6[181] | AACAAGAGGGGTTTCAGGAGTGCAGCGAACAGAGGCTTTGACACAACGG | Structure |
| Tetrapod_7[154] | GTTCCGACCTCAGAATTAAAGATGTTTAGCATAGTGGAACCGTACGCAT | Structure |
| Tetrapod_7[175] | CTTATAAAGCCACCGCAGTCTGCCAGAGCATAACCGCGCAGATTGT | Structure |
| Tetrapod_7[46]_nondocking_mid_arm | GGGCAACAGCTATGGTTTGTAGCGCAAATGAATATCAAATTTGAGGACAA | mid arm position structure |
| Tetrapod_7[60] | TGCGGGAGAGGCGGTTTCCCGCTTGCGAGAAGTGAATTTAA | Structure |
| Tetrapod_7[68] | ACCGCCTGGGCGCCGCAACCACCTGCCACGTCTGGTCAGAGCCGTACAA | Structure |
| Tetrapod_8[86] | CCAGTAGCACATGAAACGCTT | Structure |
| Tetrapod_12[177] | ATAAACAGGGAAGCGCATTTGGGAACAAGTAACACCACCAGCAGC | Structure |
| Tetrapod_14[188] | CGTGGCATAGAAAAACTCCTTTTTAACCTGTAAAGGTGTTTG | Structure |
| Tetrapod_15[182] | GGACATTATTGAGCAACCGAGTTTTTGTATACAGAAAAGTAA | Structure |
| Tetrapod_20[164] | ATCGTAGTATCATATGCGTTTGATTAAACGTGCAAGTATCGGATTGCGA | Structure |
| Tetrapod_23[168] | TTTGAATTTAATTGAGATAAGTTTGTATAGATCAATGTTCGG | Structure |
| Tetrapod_25[193] | CGACAAAACATATAGATTTTTATACAGTAGGGCACCAAGTCAATTAC | Structure |
| Tetrapod_28[177] | GTTTGAATTTCGCGTCGTTTGAGCTTGGCTAACAAGTCACGAGTTGGG | Structure |
| Tetrapod_30[188] | TTTAATTAATTATTGATGAAGTTTTCCTGAAGCATGTAATATAGATTTTA | Structure |
| Tetrapod_31[182] | ATAATAATCAAACATGTGCAGTTTTTGGTAAATTTGACCATT | Structure |
| Tetrapod_36[163] | GGAGAAGCAAACTCCAATTTGGAGGGACAATCAACAGACAAATAAAAG | Structure |
| Tetrapod_39[168] | AATTACCGTCATTTGGTCAATTTTATTACGCAGAAGGAGCTA | Structure |
| Tetrapod_41[60] | AGGTCCGGCACCGCTTCTTTCGAGGCGAGGCTTATTCA | Structure |
| Tetrapod_44[170] | GCCTTCGCCATCTCATATTTATAAAAAGAGAAGCAGTC | Structure |
| Tetrapod_46[55] | CTGCAAGAACTGTTCATGACGTTTTTTACGACAAGAAACTCA | Structure |
| Tetrapod_47[175] | ATTACAGGGGTGAGCCCCGGTTTTGAGCTGATTAGCTACCTT | Structure |
| Tetrapod_9[193] | TATTTCAACCGATTGAGTTTCAGGTCAATAAGAGTTATGCGACGTTGG | Structure |
| Tetrapod_11[155] | GATAGCCACAAGATTACAGAGAGAATATTAGGTCACGTTGGTGTAGGGCC | Structure |
| Tetrapod_14[167] | GAATGGCAGTTTATTAGCAAACGTAGAAAATATTTACATTTCGCAAATTTGC | Structure |
| Tetrapod_15[161] | TCACACGGATAACCCGAACAATTAATATTTTGTTAAAATTCGTA | Structure |
| Tetrapod_16[176] | GCGAACCTCCCGACTTGCGGTTCCAGCTTGCGATCGGAAAGGGTGCCAAG | Structure |
| Tetrapod_19[143] | CAGAGCCAACAAATTACTAGAAAAATTACTCGCGGGGATTTATTAGA | Structure |
| Tetrapod_22[174] | CTGAGCAAAATCAGGAAACCAATCAATAATCGTTCAGAGCAGGCAATGGGGA | Structure |

| | | |
|---|---|---|
| Tetrapod_23[150] | ACGGTAACAACACGCGCCTTATGAAGGTTTATAAGTCTATGAG | Structure |
| Tetrapod_24[168] | ATTACATAACAATCCTCGCTTGCCTGTTCCATATTATTCGCCATGATGA | Structure |
| Tetrapod_27[154] | GAGCAAATGTTTCTAGATAACGCTTGTGTTAGCAGCGTGAGTATTACGGCA | Structure |
| Tetrapod_30[167] | GCTTGCTGAAATGATCGGCATACAAATATTCCTTTGTTTATCAACAATAGA | Structure |
| Tetrapod_31[161] | GAACAACATCTTACAAGATTGTTTGGATGAACGGGAAAGAAACTG | Structure |
| Tetrapod_33[179] | CGGTTTTTAGAATGGGAAGACTCCTGTTTTCTTGTTGGGAGACATCAG | Structure |
| Tetrapod_35[143] | TGACATTGCTGGCTTCAAAGCGAACTTATTAAAGGTGAATTATCAAAA | Structure |
| Tetrapod_38[174] | GAAGAAATAATACTGCATCAATTCTACTAATATTGTCAATCATATGTAAAAG | Structure |
| Tetrapod_39[150] | TCAAGAGCTTACATTAGATTCATACATAAAGGTGGCAACAGCCC | Structure |
| Tetrapod_40[181] | GATTGACCGTAATGGGATTTACATAAATCAGAGAACCAGTATATTTTT | Structure |
| Tetrapod_41[179] | AATATTTAAATTGTAAACGTTTTAGTTACCAGTATGTTTTGTCAAGGTAA | Structure |
| Tetrapod_42[79] | CGTCCTCTTCAGATCGCACTCCAGTTGAGGTTTTGAAGCCTTAGGAA | Structure |
| Tetrapod_43[151] | AAACACCATCACGGAAACCAGTTTTTATCAAGCACTGCACTGGCGGT | Structure |
| Tetrapod_43[49] | GATTGTGCTGGAACTGCTTGGCGTTATATAGAGCTGATACCCCAAT | Structure |
| Tetrapod_45[46] | AGCAAAATCAAAACTCAACGTTAAAATGCGAGTATTTAAAGGATTTATCA | Structure |
| Tetrapod_46[73] | TGGCGTGCGGGGAAGCGGTTGCTGTCTTTCCTTATCATAATG | Structure |
| Tetrapod_47[157] | AGCAGTCAAATCTAGCATTTGTAGTAGCATTAACATCCAGTT | Structure |
| Tetrapod_8[168] | ATATTGACGGAAATTATTCTTCAGACCGTGGCTTACTTTAATGTTAATA | Structure |
| Tetrapod_0[97] | CAATTCCACACAACGCGCACTAAATCGGAACCCTCCCGCTCA | Structure |
| Tetrapod_2[139] | AGTAACAGTGCCCGTAGGCCTTGAACCCTCATGCCTT | Structure |
| Tetrapod_3[119] | AGACGATTTAAACAGTTGAAACATTTCCTGTGTGAAATTG | Structure |
| Tetrapod_3[140] | AACAAATAGTGCCTTGGGCTGAGTCGTAATCATGGTCATA | Structure |
| Tetrapod_6[90] | TAAAATAGAGTTGCAGCAAACCACCACCAGGCCATCGATAGCAGCACCG | Structure |
| Tetrapod_6[118] | TCAGTTTATTATTCTAATGCCCCCTGCCTAGGTTGAGAGAGCCGCAGTAGC | Structure |
| Tetrapod_8[115] | CCATTTATTAAAAATACCGAACGATACCGTCACCGACTTGAG | Structure |
| Tetrapod_10[97] | AGTATTAAAGTTGAAAAGCACTAATTTATCCCAATCCAAA | Structure |
| Tetrapod_14[125]_nondocking_vertex | AAAACATCGCCAAGCTCAATCGTCTGGAAACAATGAAACTAAACGAT | vertex position structure |
| Tetrapod_15[98] | CTACATCTTTAGGGGAATTGAGGAAGGTTCAGAGGTGACGCAAATTGGGAA | Structure |
| Tetrapod_18[104] | AATCAATAGTAAAGTAAAGAGAAGTGATAAATAAGGCGT | Structure |
| Tetrapod_18[132] | CGGGTATTAAACCAAGCGACAATAAGGCATTAAGAAT | Structure |
| Tetrapod_19[133] | GTTCAGCTTCCAAGAACATCGTAAATCAAGATTAGTTGCT | Structure |
| Tetrapod_22[111] | ATTTCACAGAGAACAAGCAATGCACCC | Structure |
| Tetrapod_23[84] | AGGTTTTAAACCTCCGGCTTATGGTTTGAAATACCGA | Structure |
| Tetrapod_24[125] | AACTCGCTACGGCGCCCGACAATGACAACAACCGATTATCTG | Structure |
| Tetrapod_26[97] | CTTGCAGGCATTAAACACCTAAATATCTGCATATGATGTC | Structure |
| Tetrapod_30[125] | GATAGTTGCGCAATTGCTAAACAACTCTGAAACGACATCAGCTGGCA | Structure |
| Tetrapod_31[98] | CTGTATGGCACCAGGGTAAAATACGTAATTATTCGGTAGCAAGCGGTTTTG | Structure |
| Tetrapod_34[111] | CAAAATATGCAGATACTCATTCCACAACTAAAGAGGAA | Structure |
| Tetrapod_35[84] | TAGCGTTCTGGAAGTTATAACGCCATACCACGTTAGTA | Structure |
| Tetrapod_35[133] | GTAGATTTAATAAATCGCTAAATTGACCTGGAAGAGTTTC | Structure |
| Tetrapod_38[111] | ATTCATTATAAGCAATAAAGGCAGTTA | Structure |
| Tetrapod_38[132] | CATTATGCATAAAATACAGGCAAGGCAAACCAATTCTGCTCAACAAATAT | Structure |
| Tetrapod_42[118] | TTAACAGAGGTTCGACATTGCCTTGCCGGAGTAAGCG | Structure |
| Tetrapod_43[91] | ACAAAGGCTATCAGGTAGTGAGCGACTTATCACGACAGT | Structure |
| Tetrapod_46[118] | CAGGGTTAGCAATAGGAACGCATCTGCCA | Structure |
| Tetrapod_46[139] | CGAAGAAATAATTATTTTTGTTAAATCAGACAAGAGACCGTTCTAAAGCAA | Structure |
| Tetrapod_47[91] | CTCATGGCTATTTTTGAGCAAAGGTGTTATCTCGGAT | Structure |
| Tetrapod_47[133] | AGATGGTATTCAAATCGATGAACGGTAATCGCATTAACGCGTCTATGGGCG | Structure |
| Tetrapod_0[37] | TGAGCTAACTCACATCGGCTTT | Structure |
| Tetrapod_1[19] | TTTCGGCTACCATTAATTGGCGAAAGGACATCGGCTTT | Structure |
| Tetrapod_3[192] | CGTTATCACCGCGTTTGCCATCTTTTCCATCGGCTTT | Structure |
| Tetrapod_4[38] | GCACACCAGTGGGGCGCCAGGGTGGTTCATCGGCTTT | Structure |
| Tetrapod_4[211] | TTTCGGCTACATAATCAAACCAGTAAGCCATCGGCTTT | Structure |
| Tetrapod_6[192] | TTCCGAATAGCCCGAGATAGCATCGGCTTT | Structure |
| Tetrapod_9[19] | TTTTCGTAATCATCAAGGAACGGTACAAAGCATCAGAACTACTAATGCTTTT | Structure |
| Tetrapod_10[44] | TCTGCCAGAAAAGGGATTTTAGACAGGAACTACTAATGCTTTT | Structure |

| | | |
|---|---|---|
| Tetrapod_12[44] | TAGCCAGAACAAACTATCGGCCTTGCGAACTACTAATGCTTTT | Structure |
| Tetrapod_12[211] | TTTACCCTGAACACGGAATACTTT | Structure |
| Tetrapod_17[19] | TTTTTGAGTAACTAGCGATAGTTT | Structure |
| Tetrapod_20[184] | ACAAATTCTTACCAGTATAGAACTACTAATGCTTTT | Structure |
| Tetrapod_20[211] | TTTTCGTAATCATCAAGAAGCCAACGAATAATATCGAACTACTAATGCTTTT | Structure |
| Tetrapod_22[184] | TTTTACAAAATCGCGCAGAGAACTACTAATGCTTTT | Structure |
| Tetrapod_25[19] | TTTTCGTAATCATCAAGAGTACCGCCCATCGGAACGAACTACTAATGCTTTT | Structure |
| Tetrapod_26[44] | CAGACCCTCACGTACTCAGGAGGTTTGAACTACTAATGCTTTT | Structure |
| Tetrapod_28[44] | AGCTAGCGTAGCATTCCACAGACAGCGAACTACTAATGCTTTT | Structure |
| Tetrapod_28[211] | TTTGTAGTGTCACTCCATGCATTT | Structure |
| Tetrapod_33[19] | TTTACCTGCTCCAAAACCAAATTT | Structure |
| Tetrapod_36[184] | TTGGGATTAGAGAGTACCTGAACTACTAATGCTTTT | Structure |
| Tetrapod_36[211] | TTTTCGTAATCATCAAGTTAATTGCTTATTTTCATGAACTACTAATGCTTTT | Structure |
| Tetrapod_38[184] | AGGATTTTAAGAACTGGCTGAACTACTAATGCTTTT | Structure |
| Tetrapod_41[19] | TTTCGGCTACGCCATTCGCGCAGACCTTCATCGGCTTT | Structure |
| Tetrapod_41[39] | CAGGGAAACCAGGCAAAGCCATCGGCTTT | Structure |
| Tetrapod_44[211] | TTTCGGCTACAATGTGTAGGCCCCAAAACATCGGCTTT | Structure |
| Tetrapod_46[34] | TAACGCCAGCATCGGCTTT | Structure |
| Tetrapod_47[193] | AATAAAAATGCGCATCGGCTTT | Structure |
| Tetrapod_8[213] | TTTAGACAAAAGGGCGACAGGTTTACAAAGCGTACAGAGATAGAACCCTTTT | Structure |
| Tetrapod_10[213] | TTTCCAAAAGAACTGGCATAATAATAAAAGTCAGGAGAATTAACTGAACTTT | Structure |
| Tetrapod_24[214] | TTTACTTGCCCTCTCTGTAGTACGGTCTCCAAAACGTTGAAAATCTCCATTT | Structure |
| Tetrapod_37[16] | TTTGAATGACCATAAATCAACAGTTCACCGGATTTCATCAAGAGTAATCTTT | Structure |
| Tetrapod_0[214] | TTTCGGCTACGCCGTCGAGAGGGTTGTACCAGGTTG | Structure |
| Tetrapod_2[214] | TTTCGGCTACGTCATACATGGCTTTTTAC | Structure |
| Tetrapod_3[16] | TTTCGGCTACGCGGGCGCTAGGGCGCGAAGAAACGTTGCGTGAG | Structure |
| Tetrapod_5[16] | TTTCGGCTACGCTTTCCTCGTTAGAACGA | Structure |
| Tetrapod_6[214] | TTTCGGCTACGGTTGAGTGCGGATAAGTCATCGGCTTT | Structure |
| Tetrapod_8[213] | TTTAGACAAAAGGGCGACAGGTTTACAAAGCGTACAGAGATAGAACCCT | Structure |
| Tetrapod_10[213] | TTTCCAAAAGAACTGGCATAATAATAAAAGTCAGGAGAATTAACTGAAC | Structure |
| Tetrapod_11[16] | TTTTCGTAATCATCAAGCCTTGCTGAAATCCTTGAGAAGACAAATCCTCAA | Structure |
| Tetrapod_13[16] | TTTTCGTAATCATCAAGACTTTACAACGAACGTTTTTGCGTATACTTCAAA | Structure |
| Tetrapod_14[214] | TTTTCTGACCTGCAGCGCCAATTT | Structure |
| Tetrapod_15[16] | TTTTCGTAATCATCAAGTGGTAATATAAGTATTAGGAACTACTAATGCTTTT | Structure |
| Tetrapod_19[16] | TTTCTTAGATTAAGACGCTGAAAACAATTATCATATTAATTTTAAAAGT | Structure |
| Tetrapod_21[16] | TTTACGCGAGAAAACTTTTAATCGCAACCATATCTGAATAATGGAAGGG | Structure |
| Tetrapod_22[214] | TTTTCGTAATCATCAAGGGCGAATTATCCGGTATTGAACTACTAATGCTTTT | Structure |
| Tetrapod_23[16] | TTTTTAGAACCTAGACAAAGATTT | Structure |
| Tetrapod_24[214] | TTTACTTGCCCTCTCTGTAGTACGGTCTCCAAAACGTTGAAAATCTCCA | Structure |
| Tetrapod_27[17] | TTTTCGTAATCATCAAGGAGGGTAGCACCAGACGCAAAAGGCTTTT | Structure |
| Tetrapod_30[214] | TTTAAAAAAAGGGGAAACGATTTT | Structure |
| Tetrapod_39[16] | TTTTTGACAAGAAGAAAACGATTT | Structure |
| Tetrapod_46[214] | TTTCGGCTACCCGCCTGAAACCCGTCGGCATCGGCTTT | Structure |
| Tetrapod_16[214] | TTTTCGTAATCATCAAGCTAAGAACGTTTTGGTGCCGCTGCGCGCGATTAACGTTT | Structure |
| Tetrapod_18[214] | TTTTCGTAATCATCAAGCCATCCTAATTTACTGGGGCACCATCATATTGCAAACTTT | Structure |
| Tetrapod_34[214] | TTTTCGTAATCATCAAGTTGGGGCGCTTTTGATAATAGCAAATGTGAGCGAACGGCGTTT | Structure |
| Tetrapod_40[214] | TTTCGGCTACATTCTCCGTTTTTAGACGGAGGGTACTGGCCAAAGAATATTT | Structure |
| Tetrapod_42[214] | TTTCGGCTACACAGGAAGATTTGAAACGCGATTAAGTTCATTT | Structure |
| Tetrapod_43[16] | TTTCGGCTACTTCCATGAATTTATCCGGTTTATGTAATCATTT | Structure |
| Tetrapod_7[16]_docking_a | TTTCGGCTACTTTCTTTTCGTATAACGTCATCGGCTTTCCTCCTCCTCCTCCT | docking strand a |
| Tetrapod_31[16]_docking_b | TTTTCGTAATCATCAAGCCTCATAGTGATTATACCGAACTACTAATGCTTTTCCTCCTCCTCCTCCT | docking strand b |
| Tetrapod_38[214]_docking_c | TTTTCGTAATCATCAAGCATTATACCCTTTATTTCGAACTACTAATGCTTTTCCTCCTCCTCCTCCT | docking strand c |

| | | |
|---|---|---|
| Tetrapod_28_47[16]_docking_d | TTTCGGCTACGGTTTTCCCGTAGGGAAACATCGGCTTTCCTCCTCCTCCTCCT | docking strand d |
| Tetrapod_29[17]_biotin_b1 | /5Biosg/TTTTCGTAATCATCAAGAAGCGCGAAGATAAATTTAGCCGGCTGACCATTCATT | biotinylated strand b1 |
| Tetrapod_35[16]_biotin_b2 | TTTATAGCGAGAGGCTTTTGACGATAATGTTACTGTGTCGAAATCCGCGTTT/3Bio/ | biotinylated strand b2 |
| Tetrapod_32[215]_biotin_c1 | /5Biosg/TTTTCGTAATCATCAAGAACGCAAGGTTTTATTTTATTCAAAACGCAACAGCT | biotinylated strand c1 |
| Tetrapod_44[191]_biotin_c2 | AGAAATGCAATGCCTGAGTCATCGGCTTT/3Bio/ | biotinylated strand c2 |
| Tetrapod_8_45[16]_biotin_d1 | /5Biosg/TTTCGGCTACCTGCGTGTTTTTTCTTCACACCGTACTTTTTTCAGGAGCC | biotinylated strand d1 |
| Tetrapod_26[214]_biotin_d2 | TTTGACATCACGAAGGTGTGTCTTGTGATGATGAGCGATGCCAGAGTCTTTT/3Bio/ | biotinylated strand d2 |
| Tetrapod_7[16]_nondocking_a | TTTCGGCTACTTTCTTTTCGTATAACGTCATCGGCTTT | structure at position docking strand a (nondocking a) |
| Tetrapod_31[16]_nondocking_b | TTTTCGTAATCATCAAGCCTCATAGTGATTATACCGAACTACTAATGCTTTT | structure at position docking strand b (nondocking b) |
| Tetrapod_38[214]_nondocking_c | TTTTCGTAATCATCAAGCATTATACCCTTTATTTCGAACTACTAATGCTTTT | structure at position docking strand c (nondocking c) |
| Tetrapod_47[16]_nondocking_d | TTTCGGCTACGGTTTTCCCGTAGGGAAACATCGGCTTT | structure at position docking strand d (nondocking d) |
| Tetrapod_29[17]_nonbiotin_b1 | TTTTCGTAATCATCAAGAAGCGCGAAGATAAATTTAGCCGGCTGACCATTCATT | structure at position biotinylated strand b1 (nonbiotinylated b1) |
| Tetrapod_35[16]_nonbiotin_b2 | TTTATAGCGAGAGGCTTTTGACGATAATGTTACTGTGTCGAAATCCGCGTTT | structure at position biotinylated strand b2 (nonbiotinylated b2) |
| Tetrapod_32[215]_nonbiotin_c1 | TTTTCGTAATCATCAAGAACGCAAGGTTTTATTTTATTCAAAACGCAACAGCT | structure at position biotinylated strand c1 (nonbiotinylated c1) |
| Tetrapod_44[191]_nonbiotin_c2 | AGAAATGCAATGCCTGAGTCATCGGCTTT | structure at position biotinylated strand c2 (nonbiotinylated c2) |
| Tetrapod_45[16]_nonbiotin_d1 | TTTCGGCTACCTGCGTGTTTTTTCTTCACACCGTACTTTTTTCAGGAGCC | structure at position biotinylated strand d1 (nonbiotinylated d1) |
| Tetrapod_26[214]_nonbiotin_d2 | TTTGACATCACGAAGGTGTGTCTTGTGATGATGAGCGATGCCAGAGTCTTTT | structure at position biotinylated strand d2 (nonbiotinylated d2) |
| Tetrapod_14[125]_docking_vertex | AAAACATCGCCAAGCTCAATCGTCTGGAAACAATGAAACTAAACGATTTTCCTCCTCCTCCTCCT | vertex docking strand |
| Tetrapod_7[46]_docking_mid_arm | GGGCAACAGCTATGGTTTGTAGCGCAAATGAATATCAAATTTGAGGACAATTTCCTCCTCCTCCTCCT | mid arm docking strand |
| short | GCCGATGGTAGCCG | short staple scaffold |
| long | AGCATTAGTAGTTCCTTGATGATTACGA | long staple scaffold |
| Tetrapod_7[16]_dimer_connector_monomer_1 | GTACGGTCAACGGCTACTTTCTTTTCGTATAACGTCATCGGCGTACGGTCAA | dimerization connector strands for monomer 1 |
| Tetrapod_5[16]_dimer_connector_monomer_1 | GTACGGTCAACGGCTACGCTTTCCTCGTTAGAACGA | dimerization connector strands for monomer 1 |
| Tetrapod_3[16]_dimer_connector_monomer_1 | GTACGGTCAACGGCTACGCGGGCGCTAGGGCGCGAAGAAACGTTGCGTGAG | dimerization connector strands for monomer 1 |
| Tetrapod_15[16]_dimer_connector_monomer_1 | GTACGGTCAATCGTAATCATCAAGTGGTAATATAAGTATTAGGAACTACTAATGCT | dimerization connector strands for monomer 1 |
| Tetrapod_21[16]_dimer_connector_monomer_1 | ACGTTAGCATACGCGAGAAAACTTTTAATCGCAACCATATCTGAATAATGGAAGGG | dimerization connector strands for monomer 1 |
| Tetrapod_23[16]_dimer_connector_monomer_1 | ACGTTAGCATTTAGAACCTAGACAAAGAACGTTAGCAT | dimerization connector strands for monomer 1 |
| Tetrapod_19[16]_dimer_connector_monomer_1 | ACGTTAGCATCTTAGATTAAGACGCTGAAAACAATTATCATATTAATTTTAAAAGT | dimerization connector strands for monomer 1 |
| Tetrapod_13[16]_dimer_connector_monomer_1 | ACGTTAGCATTCGTAATCATCAAGACTTTACAACGAACGTTTTTGCGTATACTTCAAA | dimerization connector strands for monomer 1 |

| | | |
|---|---|---|
| Tetrapod_7[16]_dimer_connector_monomer_2 | ATGCTAACGTCGGCTACTTTCTTTTCGTATAACGTCATCGGCATGCTAACGT | dimerization connector strands for monomer 2 |
| Tetrapod_5[16]_dimer_connector_monomer_2 | ATGCTAACGTCGGCTACGCTTTCCTCGTTAGAACGA | dimerization connector strands for monomer 2 |
| Tetrapod_3[16]_dimer_connector_monomer_2 | ATGCTAACGTCGGCTACGCGGGCGCTAGGGCGCGAAGAAACGTTGCGTGAG | dimerization connector strands for monomer 2 |
| Tetrapod_15[16]_dimer_connector_monomer_2 | ATGCTAACGTTCGTAATCATCAAGTGGTAATATAAGTATTAGGAACTACTAATGCT | dimerization connector strands for monomer 2 |
| Tetrapod_14_21[16]_dimer_connector_monomer_2 | TTGACCGTACACGCGAGAAAACTTTTAATCGCAACCATATCTGAATAATGGAAGGG | dimerization connector strands for monomer 2 |
| Tetrapod_23[16]_dimer_connector_monomer_2 | TTGACCGTACTTAGAACCTAGACAAAGATTGACCGTAC | dimerization connector strands for monomer 2 |
| Tetrapod_19[16]_dimer_connector_monomer_2 | TTGACCGTACCTTAGATTAAGACGCTGAAAACAATTATCATATTAATTTTAAAAGT | dimerization connector strands for monomer 2 |
| Tetrapod_13[16]_dimer_connector_monomer_2 | TTGACCGTACTCGTAATCATCAAGACTTTACAACGAACGTTTTTGCGTATACTTCAAA | dimerization connector strands for monomer 2 |
| Tetrapod_7[16]_no_dimerization | TTTCGGCTACTTTCTTTTCGTATAACGTCATCGGCTTT | no dimerization strands |
| Tetrapod_5[16]_no_dimerization | TTTCGGCTACGCTTTCCTCGTTAGAACGA | no dimerization strands |
| Tetrapod_3[16]_no_dimerization | TTTCGGCTACGCGGGCGCTAGGGCGCGAAGAAACGTTGCGTGAG | no dimerization strands |
| Tetrapod_15[16]_no_dimerization | TTTTCGTAATCATCAAGTGGTAATATAAGTATTAGGAACTACTAATGCTTTT | no dimerization strands |
| Tetrapod_14_21[16]_no_dimerization | TTTACGCGAGAAAACTTTTAATCGCAACCATATCTGAATAATGGAAGGG | no dimerization strands |
| Tetrapod_23[16]_no_dimerization | TTTTTAGAACCTAGACAAAGATTT | no dimerization strands |
| Tetrapod_19[16]_no_dimerization | TTTCTTAGATTAAGACGCTGAAAACAATTATCATATTAATTTTAAAAGT | no dimerization strands |
| Tetrapod_13[16]_no_dimerization | TTTTCGTAATCATCAAGACTTTACAACGAACGTTTTTGCGTATACTTCAAA | no dimerization strands |

**Supplementary Table 7 DNA origami tetrapod strands.** Scaffold is p8634. Structure staples are all the strands that form the structure. Biotinylated staples are modified with biotin to immobilize origami on coverslip surfaces through a BSA-Biotin-Streptavidin-Biotin-DNA origami arrangement. Docking staples were used for DNA-PAINT experiments. Dimerization connectors are for dimer assembly.

| Strands | monomer with 4 dockings | 1 at 0 | | 7 at 1 | | 7 at 2 | | 6 at 3 | |
|---|---|---|---|---|---|---|---|---|---|
| | | **Monomer 1** | **Monomer 2** | **Monomer 1** | **Monomer 2** | **Monomer 1** | **Monomer 2** | **Monomer 1** | **Monomer 2** |
| P8634 | Y | Y | Y | Y | Y | Y | Y | Y | Y |
| Structure | Y | Y | Y | Y | Y | Y | Y | Y | Y |
| vertex position structure | Y | Y | Y | N | Y | N | N | N | N |
| mid arm position structure | Y | N | N | N | N | N | N | N | Y |
| docking strand a | Y | N | N | N | N | N | N | N | N |
| docking strand b | Y | N | Y | N | Y | N | Y | N | Y |
| docking strand c | Y | N | N | N | Y | N | N | N | Y |
| docking strand d | Y | N | Y | Y | Y | Y | Y | Y | Y |
| vertex docking strand | N | N | N | Y | N | Y | Y | Y | N |
| mid arm docking strand | N | Y | Y | Y | Y | Y | Y | Y | Y |
| biotinylated strand b1 | Y | N | N | N | N | N | N | N | N |
| biotinylated strand b2 | Y | N | N | N | N | N | N | N | N |
| biotinylated strand c1 | Y | Y | Y | Y | Y | Y | Y | Y | Y |
| biotinylated strand c2 | Y | Y | Y | Y | Y | Y | Y | Y | Y |
| biotinylated strand d1 | Y | Y | Y | Y | Y | Y | Y | Y | Y |
| biotinylated strand d2 | Y | Y | Y | Y | Y | Y | Y | Y | Y |
| structure at position docking strand a (nondocking a) | N | Y | Y | Y | Y | Y | Y | Y | Y |
| structure at position docking strand b (nondocking b) | N | Y | N | Y | N | Y | N | Y | N |
| structure at position docking strand c (nondocking c) | N | Y | Y | Y | N | N | Y | Y | N |
| structure at position docking strand d (nondocking d) | N | Y | N | N | N | N | N | N | N |
| structure at position biotinylated strand b1 (nonbiotinylated b1) | N | Y | Y | Y | Y | Y | Y | Y | Y |
| structure at position biotinylated strand b2 (nonbiotinylated b2) | N | Y | Y | Y | Y | Y | Y | Y | Y |
| structure at position biotinylated strand c1 (nonbiotinylated c1) | N | N | N | N | N | N | N | N | N |
| structure at position biotinylated strand c2 (nonbiotinylated c2) | N | N | N | N | N | N | N | N | N |
| structure at position biotinylated strand d1 (nonbiotinylated d1) | N | N | N | N | N | N | N | N | N |
| structure at position biotinylated strand d2 (nonbiotinylated d2) | N | N | N | N | N | N | N | N | N |
| short staple scaffold | Y | Y | Y | Y | Y | Y | Y | Y | Y |
| long staple scaffold | Y | Y | Y | Y | Y | Y | Y | Y | Y |
| dimerization connector strands for monomer 1 | N | Y | N | Y | N | Y | N | Y | N |
| dimerization connector strands for monomer 2 | N | N | Y | N | Y | N | Y | N | Y |
| no dimerization strands | Y | N | N | N | N | N | N | N | N |

**Supplementary Table 8 Tetrapod Pattern Mixing.** The assembly contained 30 nM scaffold P8634; 300 nM of each structure strand; 6 $\mu$M of each biotinylated strand; 4.5 $\mu$M of each short and long staple scaffold; 300 nM of both non-dimerizing strands and dimerization connector strands specific to the monomer; 5 $\mu$M of each docking strand; and 300 nM of each non-docking strand.

| Parameters | Values |
|---|---|
| Box side length | 7 for 2D, and 9 for 3D |
| Min. Net Gradient | 15,000 for 2D, and 10,000 for 3D<br>(filtering can be done later using Picasso Filter module) |
| EM Gain | 1 |
| Baseline | 100 |
| Sensitivity | 0.46 |
| Quantum efficiency (at Cy3B emission) | 0.82 |
| Pixel size | 117 nm |
| Method | MLE, integrated Gaussian for 2D, and LQ, Gaussian for 3D |
| 3D via Astigmatism | Empty for 2D, and Use a calibration file for 3D |

**Supplementary Table 9 Picasso localize module parameters.** The parameters are based on the Hamamatsu ORCA-Flash4.0 V3 digital sCMOS camera.

| Parameter | NSF 2D dataset | ASU one-redundancy 2D dataset | ASU two-redundancy 2D dataset | 0407 3D dataset |
|---|---|---|---|---|
| $N$ | 3 | 9 | 9 | 0 |
| $M$ | 13 | 49 | 49 | 13 |
| $T_{\mathrm{I}}$ | 0.15 | 0.2 | 0.2 | 95% |
| $T_{\mathrm{S}}$ | 0.5 | 0.7 | 0.8 | Not applicable |
| $W_{\mathrm{O}}$ | 1.5 | 1.5 | 1.5 | 1 |
| Alignment | Rough | Differential evolution | Differential evolution | Steps of translation followed by rotation concerning z-axis |

**Supplementary Table 10 Parameter selection.** It is done by empirically finding good values for $N$ and $M$. If origami with higher numbers of binding sites is imaged, we may need a higher value of $M$ to account for false positives. $T_{\mathrm{I}}$ and $T_{\mathrm{S}}$ were selected through a grid search. $W_{\mathrm{O}}$ was selected empirically. The best method for alignment for each dataset was empirically selected.

| **Readout Modality** | **Spatial Resolution** | **Estimated Throughput** | **Dimensional Capability** | **Cryptographic Potential** | **Ref.** |
|---|---|---|---|---|---|
| **AFM (Atomic Force Microscopy)** | Apparent ≈30-50 nm (limited by streptavidin imaging); Localization AFM ≈1 nm | ≈10–100 origami $h^{-1}$ | 1D, 2D | Low – Biotin–streptavidin labeling reduces effective resolution and bit density; limited key space due to 1D and 2D only capability | [3–5] |
| **DNA-PAINT Super-Resolution Imaging (2D / 3D / RESI)** | 5-10 nm (standard DNA-PAINT); 1 nm (RESI) | $10^3$–$10^4$ origami $h^{-1}$ (high-speed DNA-PAINT ); | 1D, 2D, 3D | High – Sequence-key docking sites; 3D scaffold-routing key space; multicolor scalability | [6–8], this work |
| **Nanopore Electrical Readout (Solid-State / Super-Resolution / Multi-Level)** | ≈4–7 nm | $10^4$–$10^5$ molecules $h^{-1}$ (single pore) | 1D | Moderate to High – sequence specific access; barcoded electrical signature | [9–11] |

**Supplementary Table 11 Benchmarking of nanoscale readout modalities for DNA-based data storage.**

## Supplementary Figures

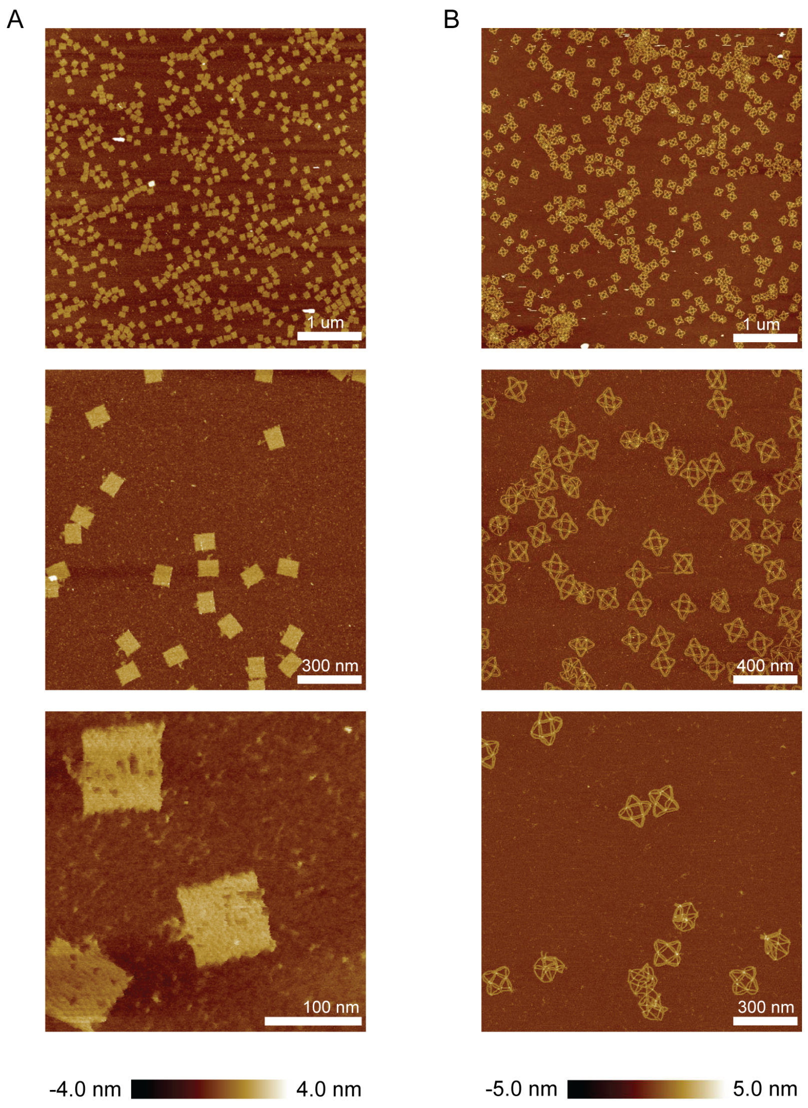


**Supplementary Fig. 1 Additional AFM images of 2D RRO and 3D wireframe cuboctahedron DNA origami.**(**A**) AFM images of 2D RRO with varying fields of view. (**B**) AFM images of 3D cuboctahedron DNA origami with varying fields of view.

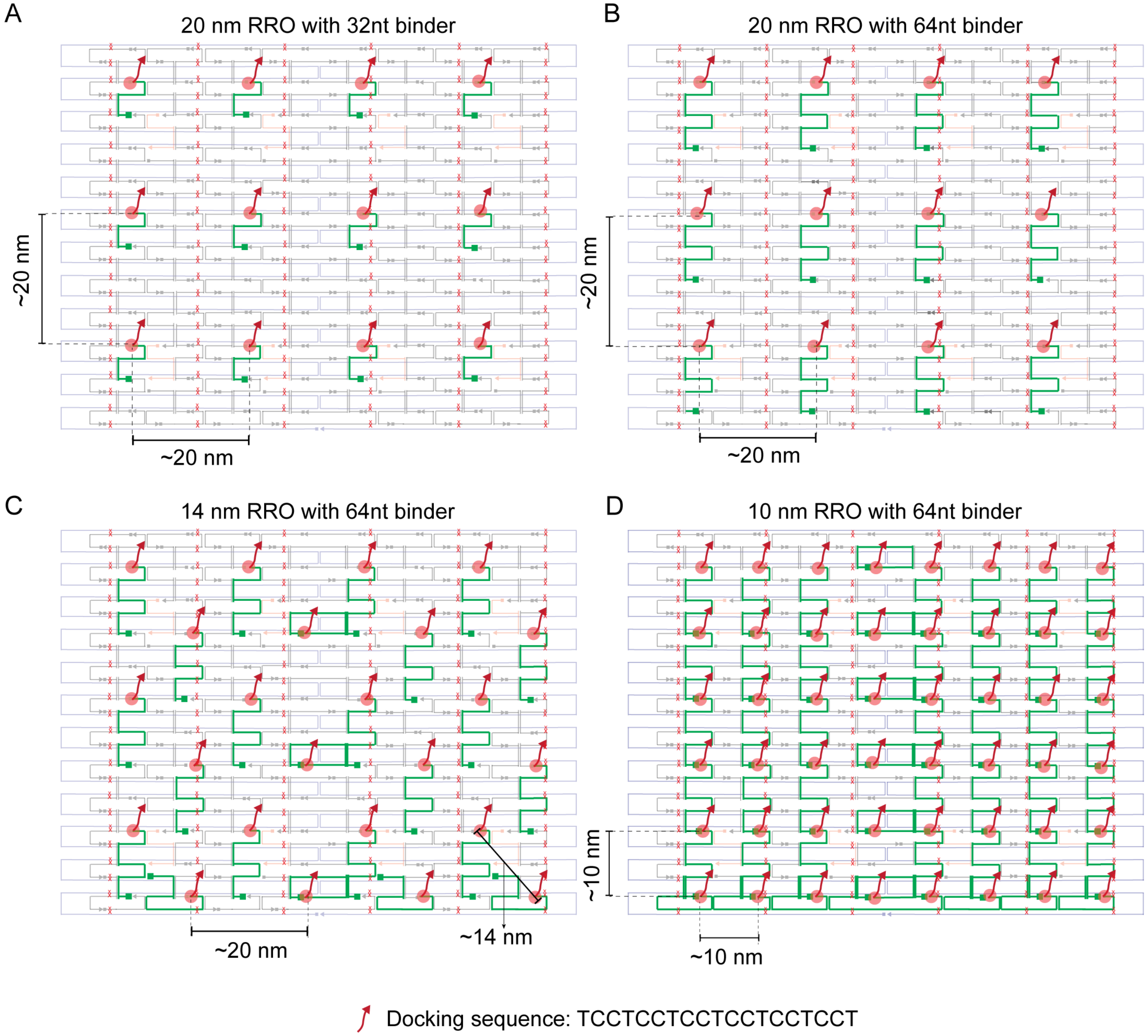


**Supplementary Fig. 2 2D RRO cadnano design showing scaffold routing and staple strands interlacing** (**A**) Map of 20 nm 2D RRO with 32 nt binder and 20 nm separation between imager binding locations. (**B**) Map of 20 nm 2D RRO with 64nt binder and 20 nm separation between imager binding locations. (**C**) Map of 14 nm 2D RRO with long 64 nt binder and 14 nm and 20 nm separation between imager binding locations. (**D**) Map of 10 nm 2D RRO with 64 nt binder and 10 nm separation between imager binding locations.

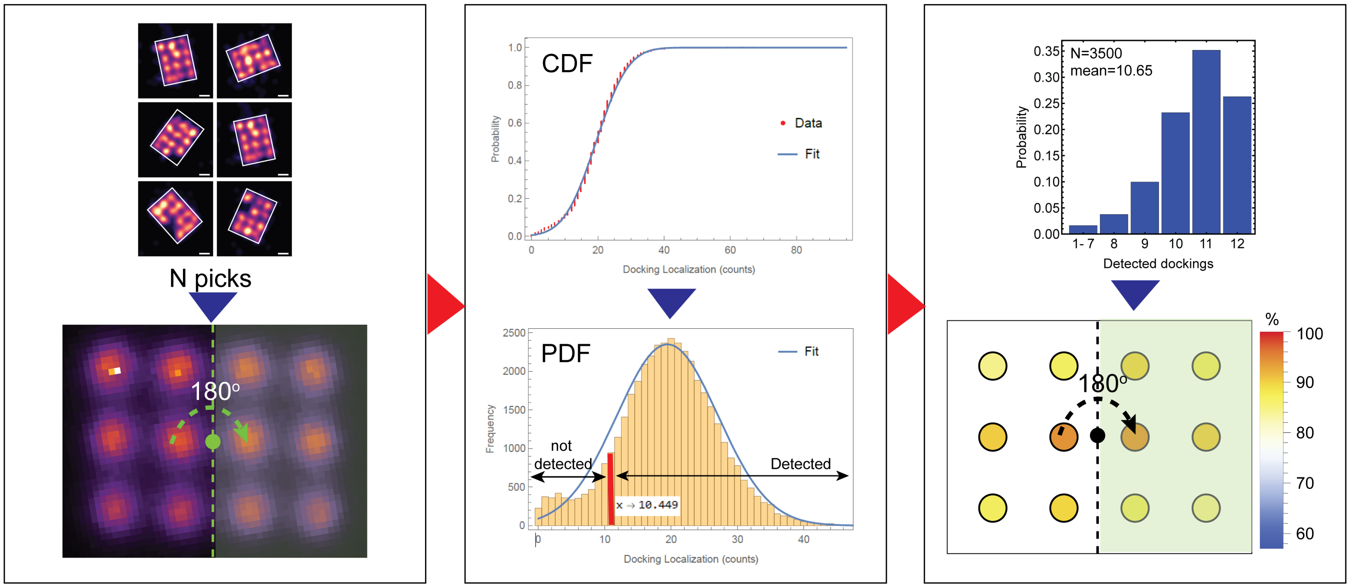


Picks alignment with Picasso Average and localization data extraction

Fitting using CDF and thresholding with FWHM

Detection efficiency

**Supplementary Fig. 3 Detection efficiency analysis procedure in 20 nm RRO with 32 nt binder.** Procedure for docking detection efficiency of 20 nm RRO with 32 nt binder. The leftmost panel shows the Picasso Render picking process (top) as an input to the Picasso average module to align the picks (bottom). The middle panel shows the incorporation distribution of localization for each docking in all picks (bottom), fitted using the cumulative distribution function (CDF) (top), and thresholded using the full-width half-maximum (FWHM). The rightmost panel shows the localization distribution after thresholding and each docking detection efficiency from all picks.

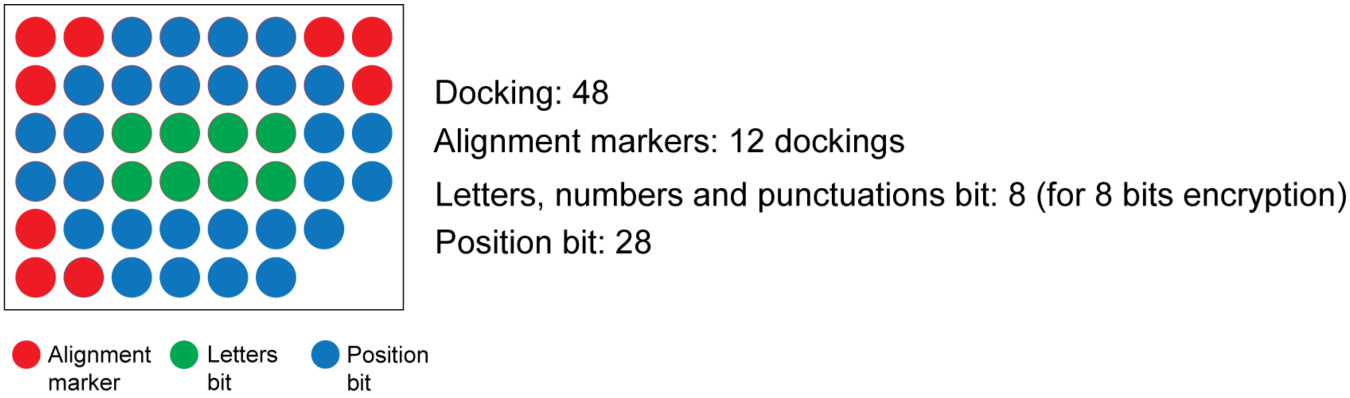


**Supplementary Fig. 4 Theoretical design of 10 nm resolution encryption pattern resulting in $2^{28}$ combinations of numbers, letters, and punctuation marks forming texts assuming 100% incorporation efficiency.** Design of 10 nm encryption showing the alignment marker with 12 docking, which breaks the symmetry of the design by including only 9 dockings in design (red), letters bit with 8 dockings (green), and position bit with 28 dockings (blue).

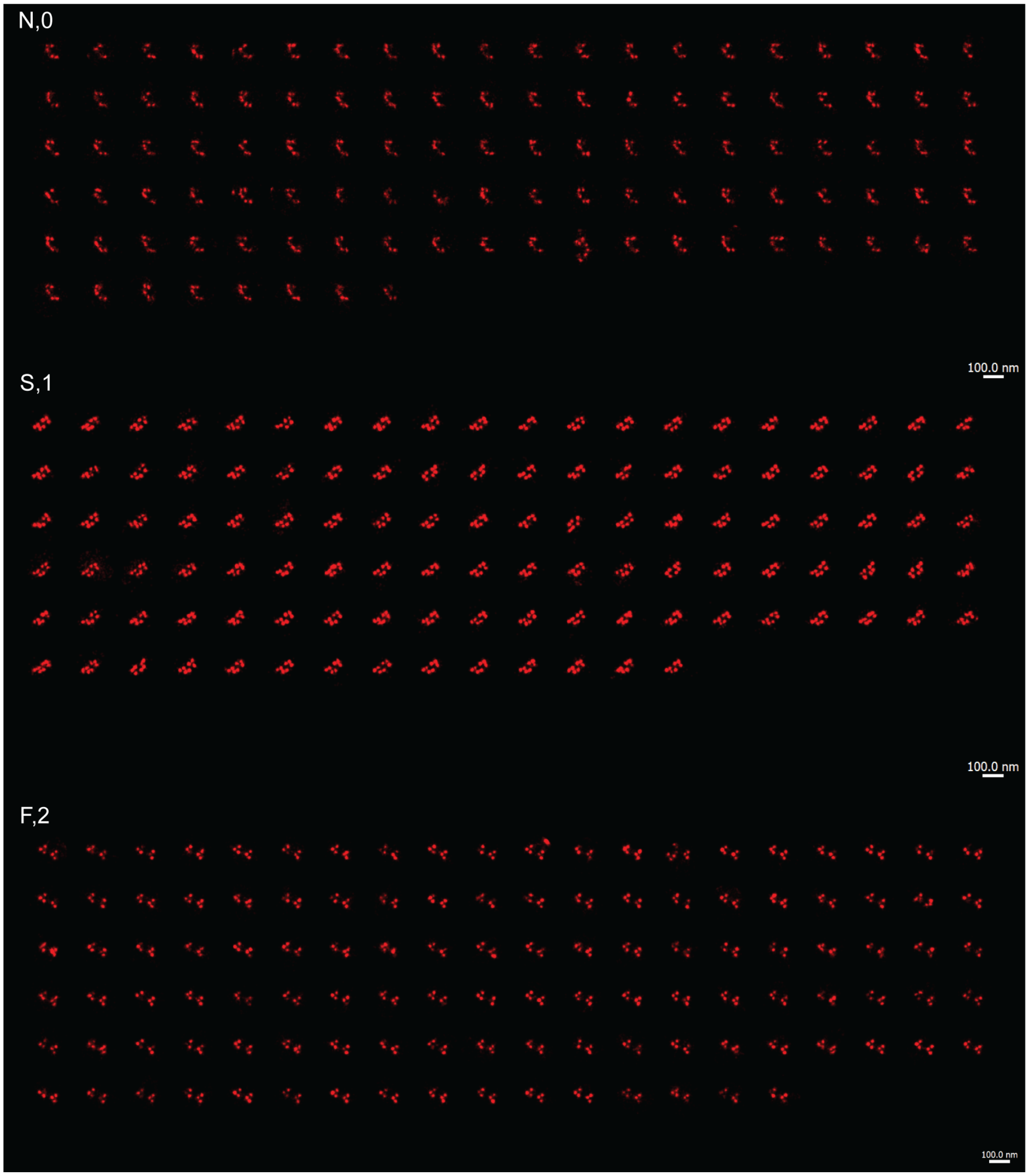


**Supplementary Fig. 5 All picks in the analyzed NSF dataset.** Full data set of 20 nm encrypted NSF following Picasso Average alignment and Picasso render unfolding with a 100 nm scale bar.

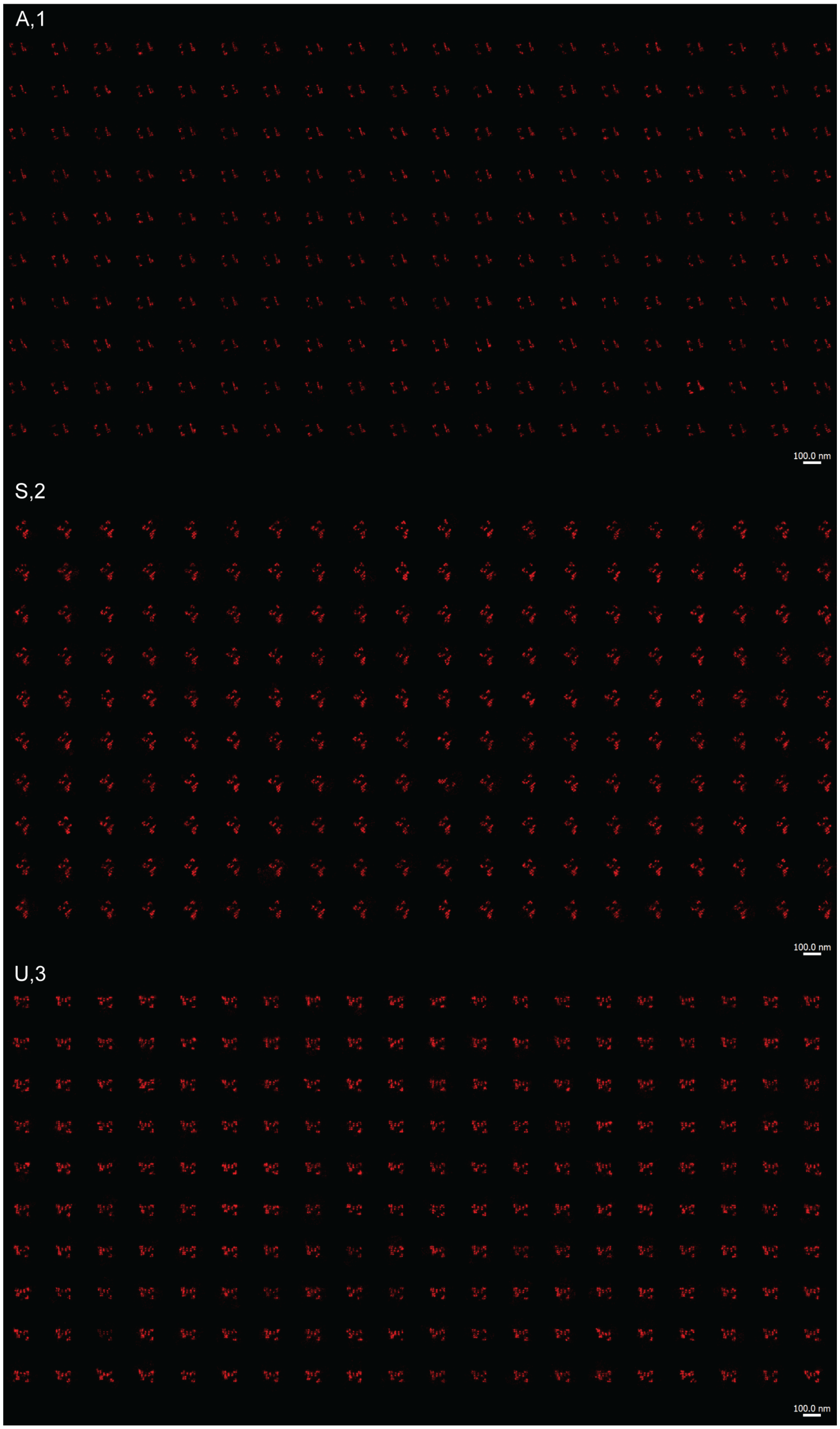


**Supplementary Fig. 6 All picks in the analyzed ASU one redundancy dataset.** Full data set of 10 nm 1 redundancy encrypted ASU following Picasso Average alignment and Picasso render unfolding with a 100 nm scale bar.

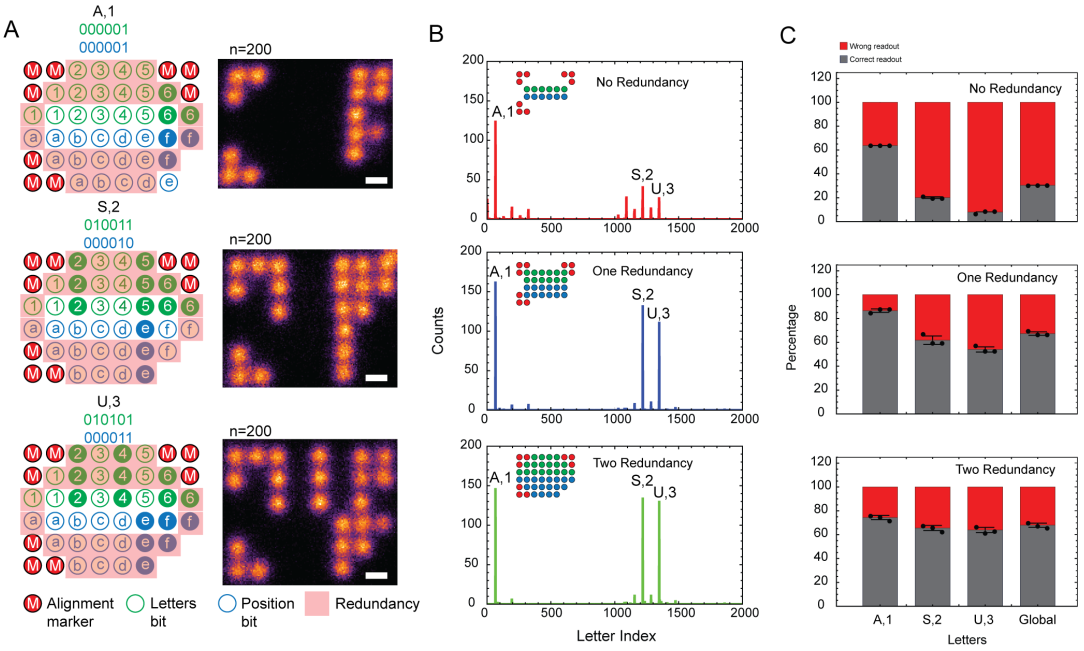


**Supplementary Fig. 7 ASU two redundancy dataset on higher density 2D RRO along with All analyzed picks.** (**A**) Pattern encryption rules for ASU two redundancy dataset that shows the alignment marker, letters bit, position bit, and the redundancy (left) and the summed DNA-PAINT images of 3 letters of ASU with a scale bar of 10 nm (right), N = 193 (top), 199 (middle), and 196 (bottom). (**B**) A readout from a single run the ASU dataset presented as letter index vs. counts analyzed by not including the redundancy (top) and the redundancy (middle and bottom). (**C**) Readout percentage of correct and incorrect readouts for each letter and global. Data are presented as mean values±SD with $N$ = 3 runs of the process in (A) using the same dataset.

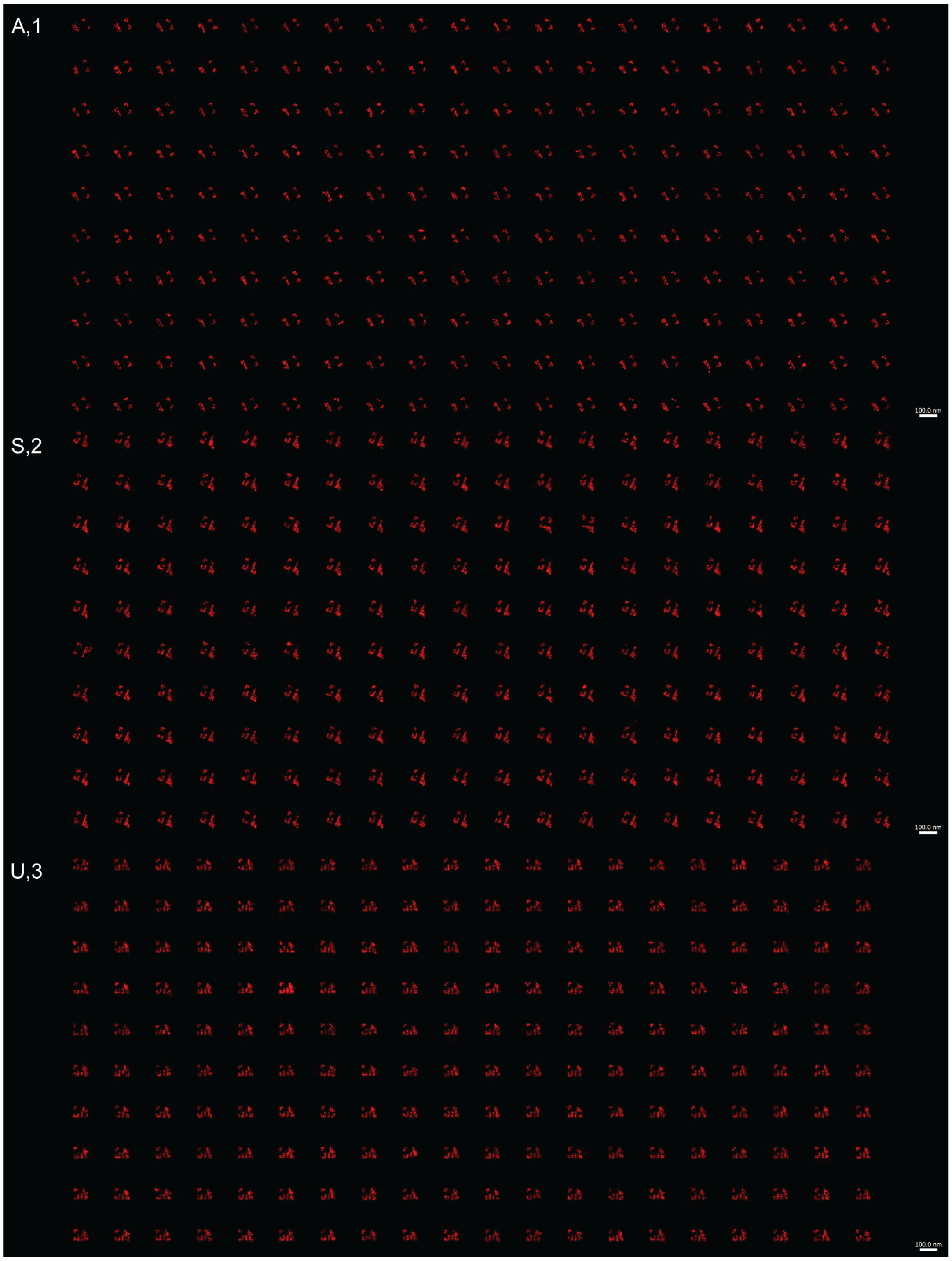


**Supplementary Fig. 8  All analyzed picks in ASU two redundancy dataset on higher density 2D RRO.** Full data set of 10 nm 2 redundancy encrypted ASU following Picasso Average alignment and Picasso render unfolding with a 100 nm scale bar.

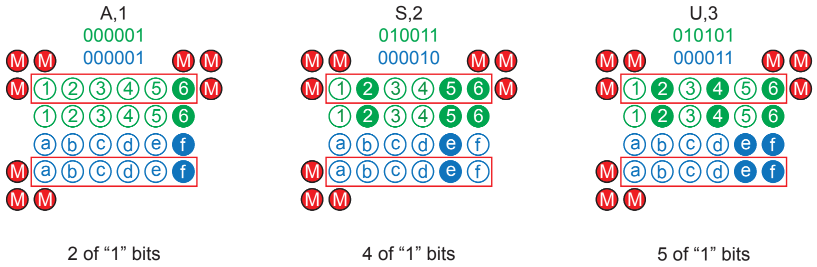


**Supplementary Fig. 9 Argument for the readout accuracy with increasing bit usage by a pattern.** Encryption pattern schematic for ASU 1 redundancy with different bit 1 usage (top). According to Figure 2 in the main text, the estimated detection efficiency of docking was 85–90%. Assuming a detection efficiency of 85%, the probability of at least 1 pair of dockings (with 1 redundancy) being detected was $1-0.15^2$, resulting in a probability of 0.978. For the A,1 pattern with 2 1 bits, the probability of correctly reading the pattern was $0.978^2$=0.956. The same estimation applies for S,2 with 4 1 bits and U,3 with 5 1 bits, resulting in probabilities of $0.978^4$=0.915 and $0.978^5$=0.895, respectively. However, alignment accuracy and k-means assignment accuracy must also be considered. Assuming an alignment and k-means accuracy of around 90%, both processes contribute to an accuracy of $\sim$81%. Using this value, the overall probability is (0.956)·(0.81)=0.77 for A,1 pattern, (0.915)·(0.81)=0.74 for S,2 pattern, and (0.895)·(0.81)=0.72 for U,3 pattern. The correct readout percentage decreases as the number of 1 bits increases. Although the values do not exactly match the experimental results, this may be due to an additional factor in alignment accuracy that tends to decrease with more 1 bits.

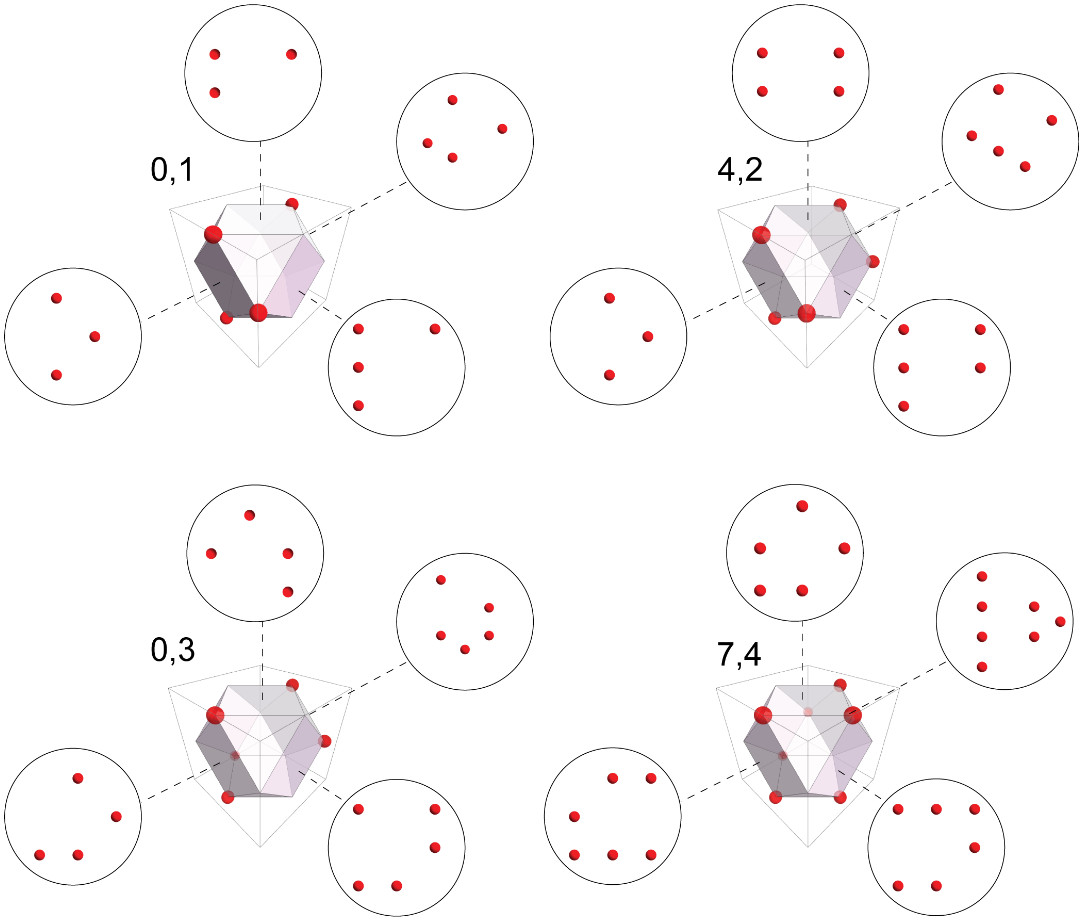


**Supplementary Fig. 10 Schematics of confused patterns due to 2D projections from 3D DNA origami encryption design.** The biotinylated strands dictate the 2D projections of each pattern when imaged using 2D DNA-PAINT only.

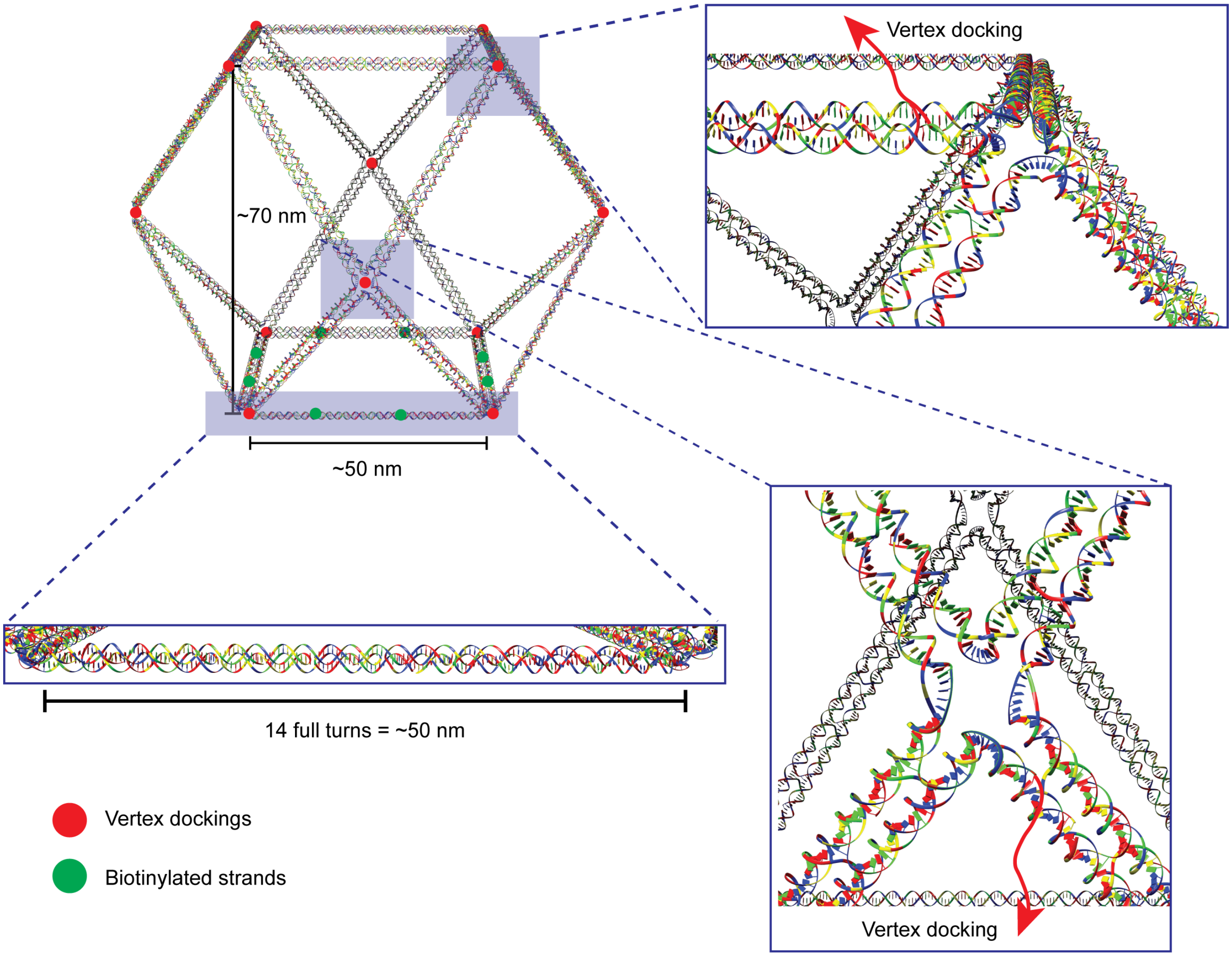


**Supplementary Fig. 11 3D wireframe cuboctahedron DNA origami design.** The 3D wireframe cuboctahedron has a height of ∼70 nm and square faces with 50 nm side length due to 14 full-duplex turns (left). Vertex dockings and biotinylated strands are indicated by red and green circles, respectively. The right panels show zoomed-in images of 2 vertices.

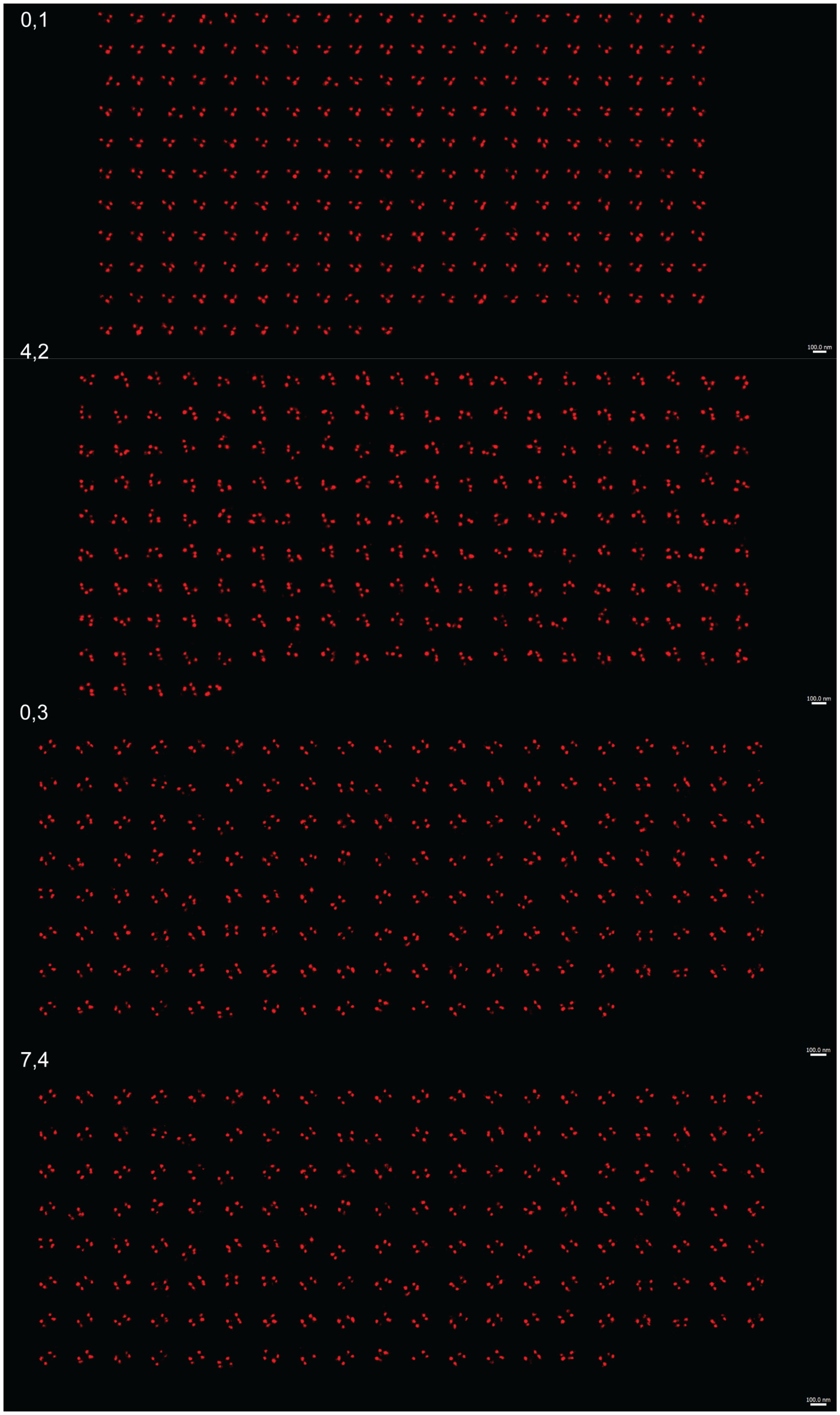


**Supplementary Fig. 12  2D view of all picks analyzed in 3D DNA-PAINT 0407 dataset.** Full dataset of 2D projection of 0407 dataset following Picasso Average alignment and Picasso render unfolding. Scale bar: 100 nm.

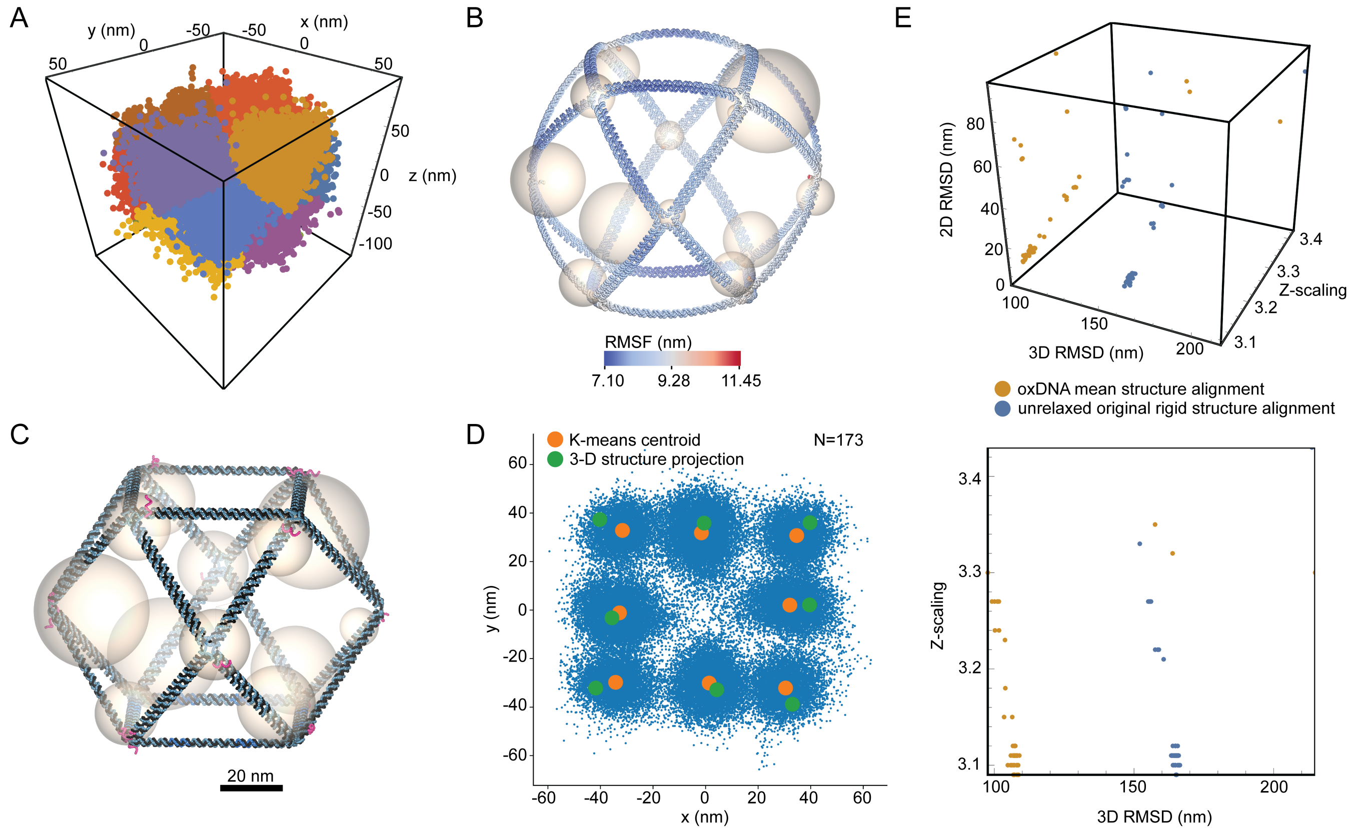


**Supplementary Fig. 13 3D clustering and alignment of DNA-PAINT experimental data and 3D cuboctahedron DNA origami structure.** (**A**) 3D k-means clustering of the 3D DNA-PAINT localization data by assigning K=12. (**B**) 3D alignment of centroids from the 3D k-means clustering results from **A** with the mean structure obtained from the oxDNA simulation. The size of each sphere depicts the distance between the k-means centroid and the center of mass of the closest docking handle. (**C**) 3D alignment of centroids from 3D k-means clustering result from **A** with an unrelaxed structure. The size of each sphere depicts the distance between the k-means centroid and the center of mass of the closest docking handle. Scale bar in **B** and **C**: 20nm. (**D**) Example of 2D alignment of the unrelaxed structure with docking handles' center of mass projected onto the x-y plane (the two stacking docking handles in $z$ direction are averaged). (**E**) (Top) Plot of 3D RMSD vs. 2D projection RMSD after 3D alignment vs. Z-scaling of the mean and unrelaxed structures. (Bottom) Plot of Z-scaling vs 3D RMSD showing the mean structure provides better RMSD, thus better alignment with the k-means centroid of experimental DNA-PAINT data compared to the unrelaxed structure alignment.

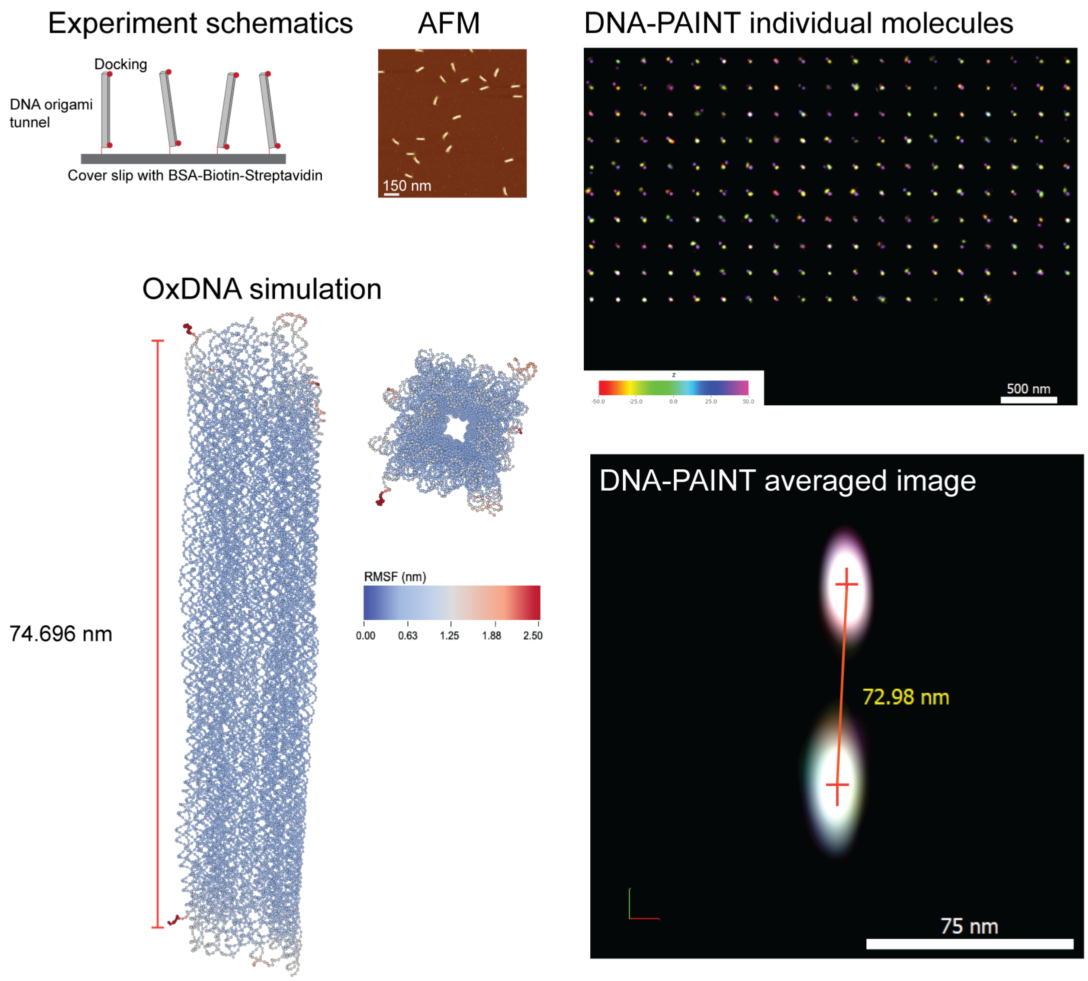


**Supplementary Fig. 14 3D tunnel DNA origami.** DNA-PAINT schematic of the 3D tunnel DNA origami (top left). AFM images of the origami (top middle), DNA-PAINT results of all picks and the averaged image showing distance of 72.98 nm (right) in agreement with the oxDNA simulation result (left)

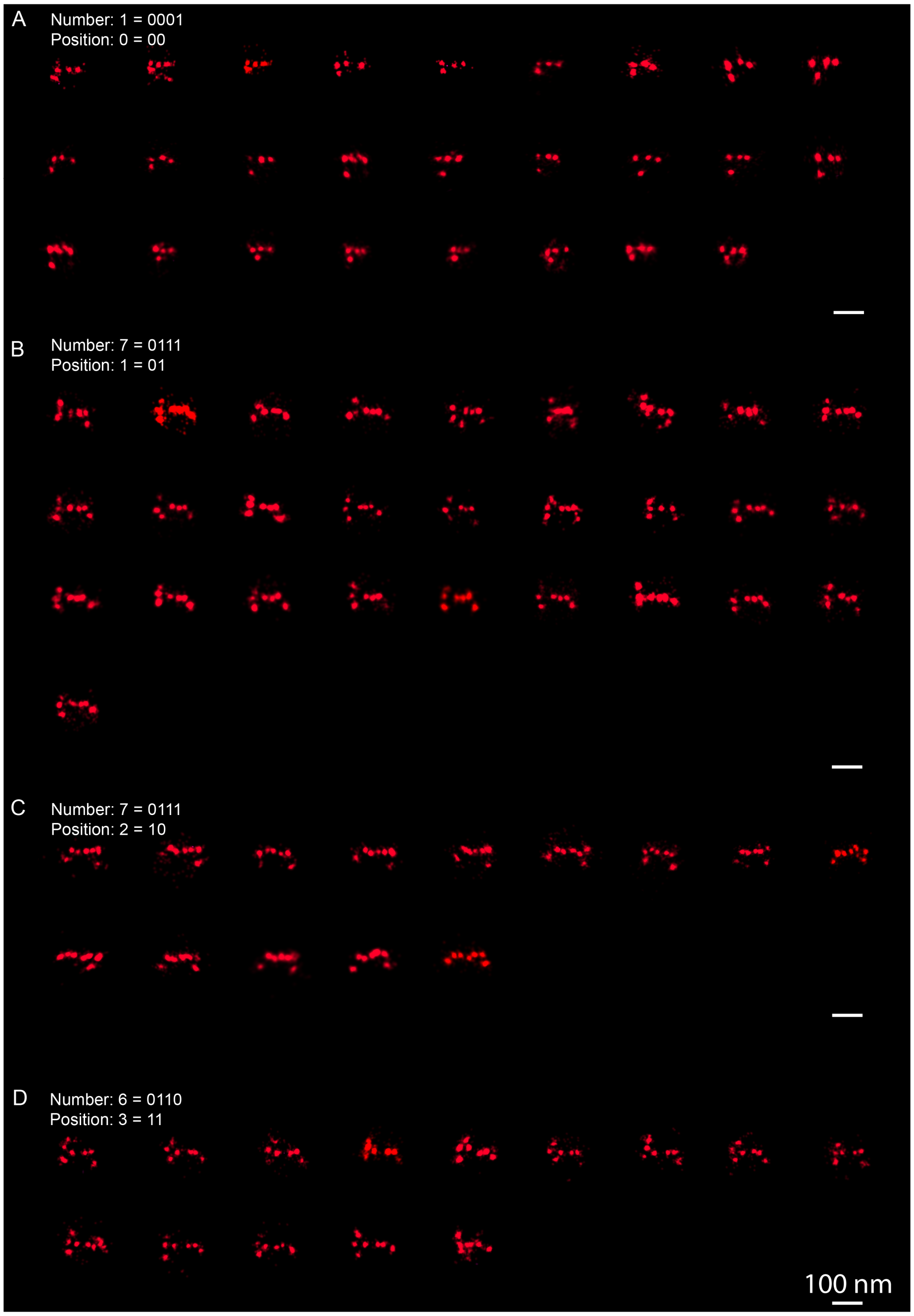


**Supplementary Fig. 15 2D view of all picks analyzed in 3D DNA-PAINT 1776 dataset from 3 different individual runs for each number.** Full dataset of 2D projection of 1776 dataset. Scale bar: 100 nm.

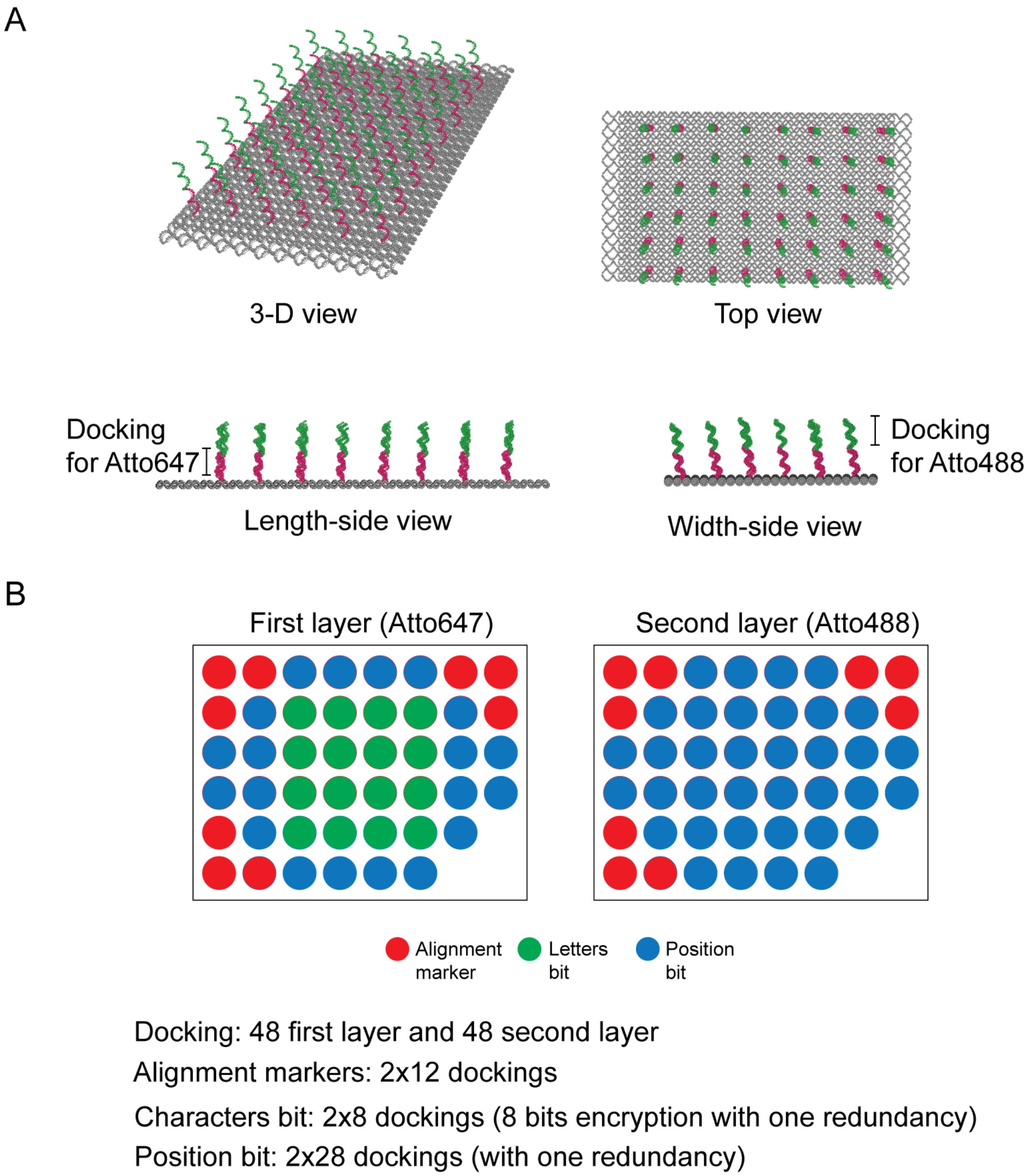


**Supplementary Fig. 16 Design of two color 2D RRO encryption for high density information.** (**A**) The RRO schematic shows two docking types for two different fluorophores. (**B**) Pattern encryption rules for two docking layers.

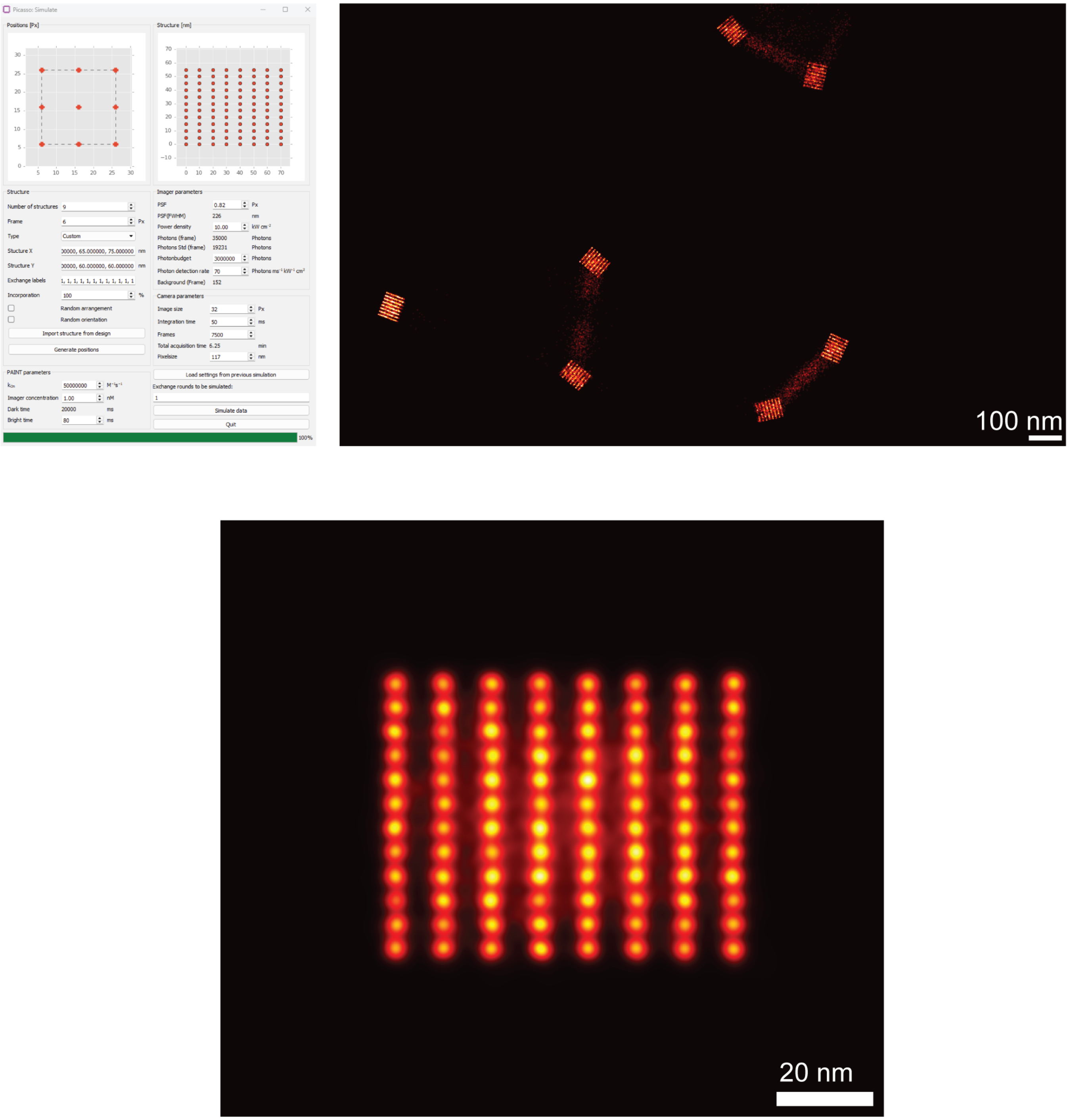


**Supplementary Fig. 17  Simulated DNA-PAINT using the Picasso Simulate module** Illustration of data density when 4 bytes are used per origami. The averaged image over 9 structures shows a clear 5 nm resolution in the vertical direction and 10 nm resolution in the horizontal direction (bottom).

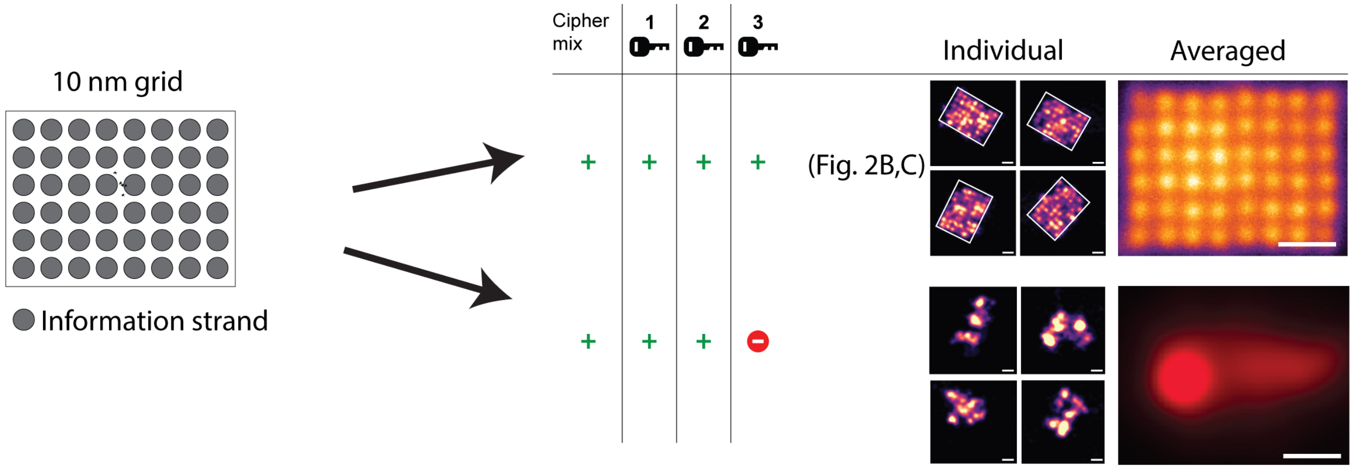


**Supplementary Fig. 18 Incomplete staple incorporation prevents encrypted pattern formation.** The structure was assembled using the M13mp18 scaffold with docking strands for a 10 nm grid (key 2), but other staples (key 3) were skipped. DNA-PAINT imaging and image reconstruction showed no detectable 10 nm grid pattern.

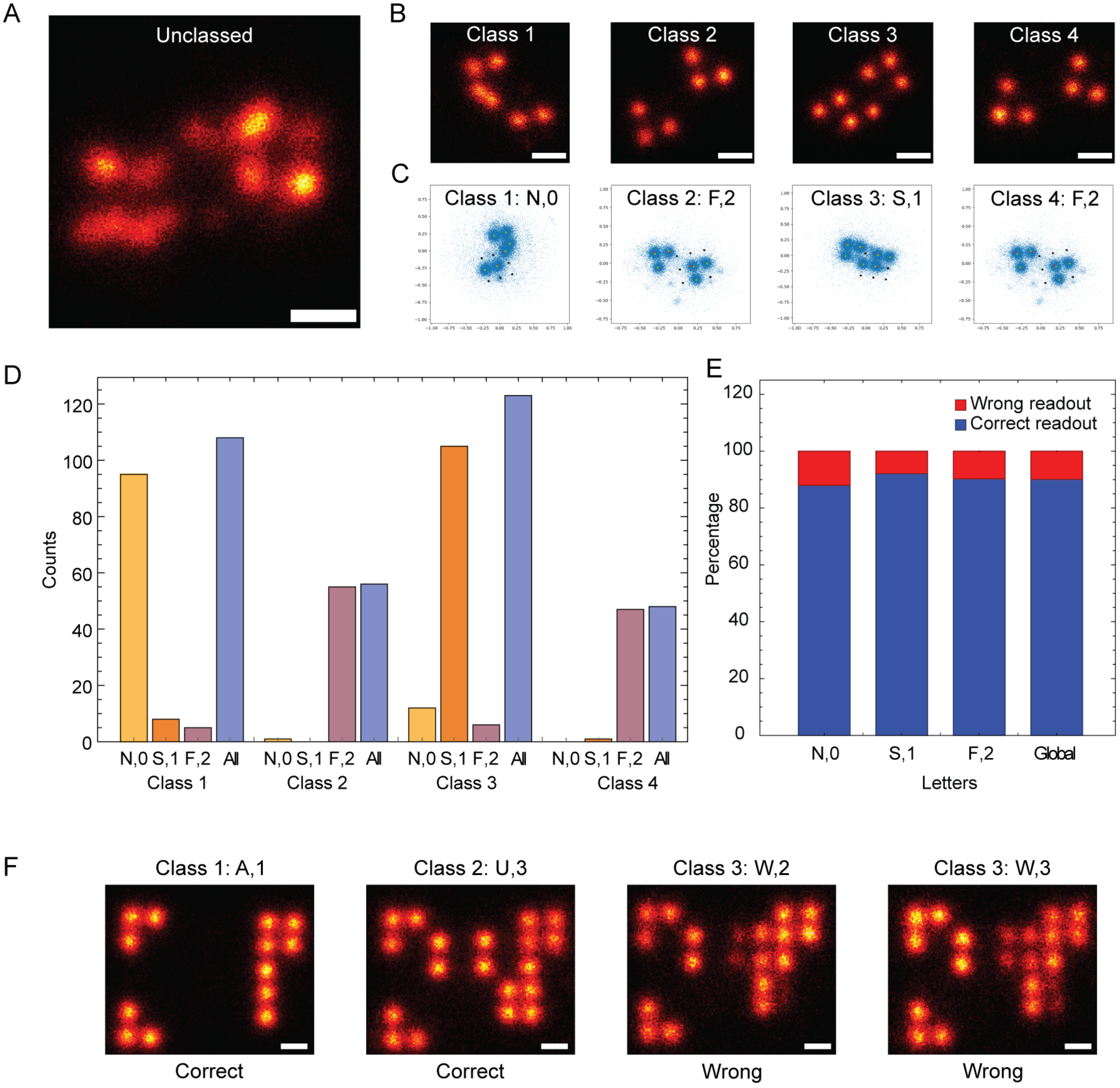


**Supplementary Fig. 19 Unsupervised classification result on NSF and ASU dataset.** (**A**) The superparticles of NSF before classification, with N = 108 for N letter, 114 for S letter, and 113 for F letter.(**B**) Superparticles of each class after a single run of classification showing 4 classes. (**C**) A representative readouts for each class in a single run with similar results in other 2 different runs. (**D**) Class members on a single run show a few misclassified patterns, thus not affecting the superparticles with similar results in other 2 different runs.(**E**) read-out accuracy for each class on a single run with similar results in other 2 different runs.(**F**) The classification of ASU is 1 redundancy dataset showing two correct classes and two wrong classes, thus making it impossible to recover ASU, with N = 200 for each A, S, and U letter.” Scale bar: 20 nm in (A) and (B), 10 nm in (F).

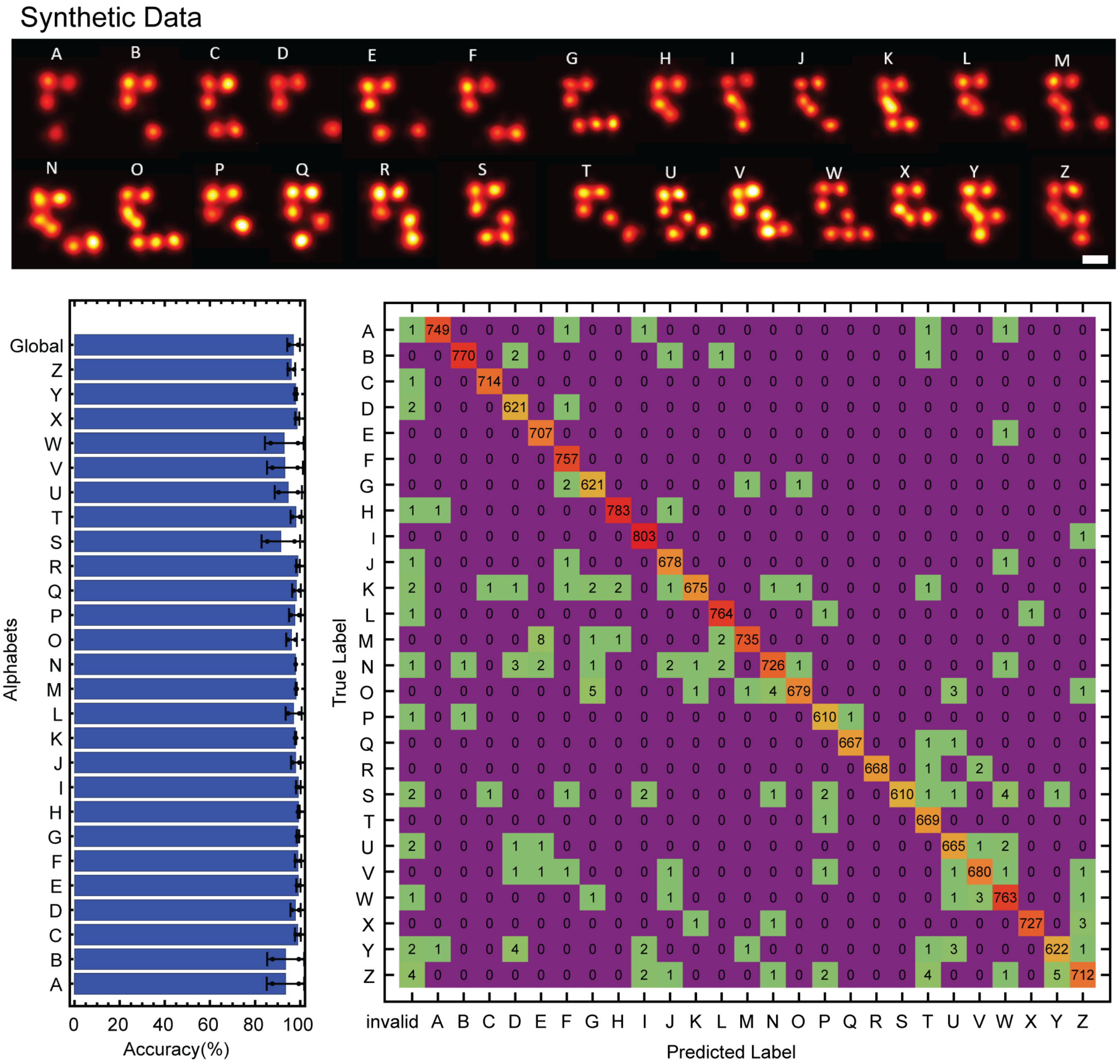


**Supplementary Fig. 20 ResNet CNN results on synthetic data generated by Picasso Simulate module of letters A-Z without position encoding.** Examples of the 26 alphabets of synthetic data generated through the Picasso Simulate module (top). The accuracy of each alphabet with ResNet-50 on two runs of classification on a dataset with N = 18,347 for run 1 and N = 4,957 (bottom left) where the data are presented as means$\pm$SD overlaid with the individual run data and the confusion matrix (bottom right) of run 1

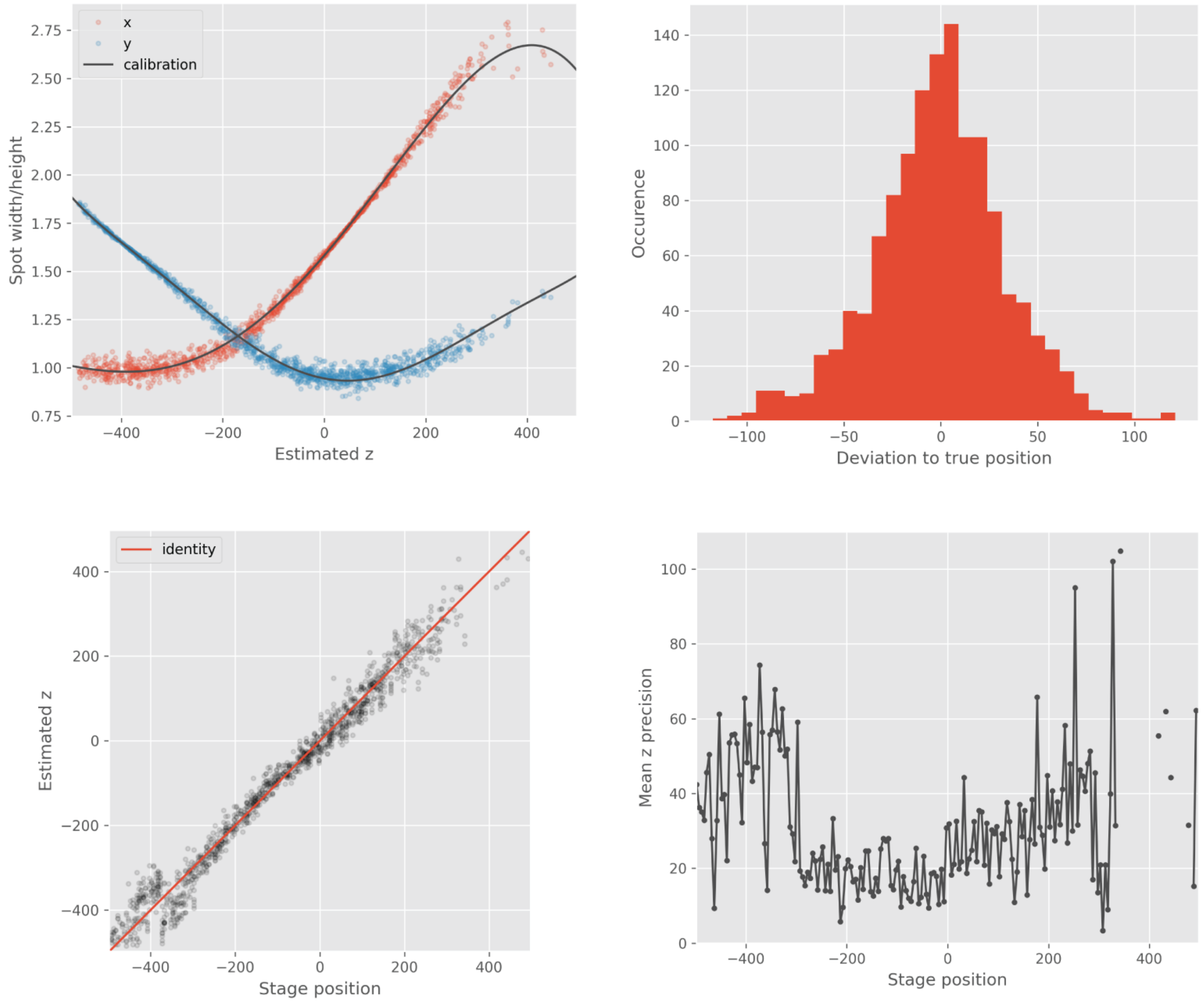


**Supplementary Fig. 21  3D calibration curve generated by the Localize feature of Picasso.** (Top left) The localization spot widths and heights with the fit. (Top right) Distribution of deviation from true position. (Bottom left) Estimation of the $z$ coordinate as a function of stage position. (Bottom right) The mean $z$ precision is a function of stage position.

## Supplementary References


1. Huijben TA, et al. (2021) Detecting structural heterogeneity in single-molecule localization microscopy data. *Nat. Commun.* 12(1):3791.
2. He K, Zhang X, Ren S, Sun J (2016) Deep residual learning for image recognition in *Proceedings of the IEEE conference on computer vision and pattern recognition.* pp. 770–778.
3. Zhang Y, et al. (2019) DNA origami cryptography for secure communication. *Nat. Commun.* 10:5469.
4. Heath GR, et al. (2021) Localization atomic force microscopy. *Nature* 594(7863):385–390.
5. Voigt NV, et al. (2010) Single-molecule chemical reactions on dna origami. *Nature nanotechnology* 5(3):200–203.
6. Dickinson GD, et al. (2021) An alternative approach to nucleic acid memory. *Nat. Commun.* 12(1):2371.
7. Reinhardt SC, et al. (2023) Ångström-resolution fluorescence microscopy. *Nature* 617(7962):711–716.
8. Dai M, Jungmann R, Yin P (2016) Optical imaging of individual biomolecules in densely packed clusters. *Nat. Nanotechnol.* 11(9):798–807.
9. Zhu J, Ermann N, Chen K, Keyser UF (2021) Image encoding using multi-level DNA barcodes with nanopore readout. *Small* 17(28):2100711.

10. Chen K, et al. (2018) Digital data storage using DNA nanostructures and solid-state nanopores. *Nano Lett.* 19(2):1210–1215.
11. Chen K, et al. (2023) Super-resolution detection of DNA nanostructures using a nanopore. *Adv. Mater.* 35(12):2207434.

nature portfolio

Corresponding author(s): Hariadi, Rizal
Wisna, Gde Bimananda Mahardika

Last updated by author(s): October 22, 2025

# Reporting Summary

Nature Portfolio wishes to improve the reproducibility of the work that we publish. This form provides structure for consistency and transparency in reporting. For further information on Nature Portfolio policies, see our Editorial Policies and the Editorial Policy Checklist.

## Statistics

For all statistical analyses, confirm that the following items are present in the figure legend, table legend, main text, or Methods section.

| n/a | Confirmed | |
|---|---|---|
| ☐ | ☒ | The exact sample size (*n*) for each experimental group/condition, given as a discrete number and unit of measurement |
| ☐ | ☒ | A statement on whether measurements were taken from distinct samples or whether the same sample was measured repeatedly |
| ☒ | ☐ | The statistical test(s) used AND whether they are one- or two-sided<br>*Only common tests should be described solely by name; describe more complex techniques in the Methods section.* |
| ☒ | ☐ | A description of all covariates tested |
| ☒ | ☐ | A description of any assumptions or corrections, such as tests of normality and adjustment for multiple comparisons |
| ☐ | ☒ | A full description of the statistical parameters including central tendency (e.g. means) or other basic estimates (e.g. regression coefficient) AND variation (e.g. standard deviation) or associated estimates of uncertainty (e.g. confidence intervals) |
| ☒ | ☐ | For null hypothesis testing, the test statistic (e.g. *F*, *t*, *r*) with confidence intervals, effect sizes, degrees of freedom and *P* value noted<br>*Give P values as exact values whenever suitable.* |
| ☒ | ☐ | For Bayesian analysis, information on the choice of priors and Markov chain Monte Carlo settings |
| ☒ | ☐ | For hierarchical and complex designs, identification of the appropriate level for tests and full reporting of outcomes |
| ☒ | ☐ | Estimates of effect sizes (e.g. Cohen's *d*, Pearson's *r*), indicating how they were calculated |

*Our web collection on statistics for biologists contains articles on many of the points above.*

## Software and code

Policy information about availability of computer code

| | |
|---|---|
| Data collection | A Benchtop Nanoimager S Mark II with total internal reflection fluorescence (TIRF) setup, with NIMos software was used for operating the microscope and acquiring the data.(https://oni.bio/nanoimager/software/nimos-software/). A Bruker multimode AFM was used for imaging DNA origami. |
| Data analysis | DNA-PAINT movies were processed using open source software FIJI to select specific region of interest (ROI) and Picasso to localize the point spread function, to render the results and to pick the DNA-PAINT molecules which is developed by Jungmann Group (https://github.com/jugmannlab/picasso). The Incorporation efficiency analysis was done using a custom Matlab script. 2D and 3D data clustering, template alignment, and analysis were performed with a custom Python and Matlab. The custom codes are available on: (https://github.com/Jonathanzhao02/smlm_classification2d) and (https://github.com/gwisna/DNA-origami-cryptography-code-and-data) The oxDNA simulation and 3D alignment of the unrelaxed and mean structure with experimental DNA-PAINT data were carried out using open source software oxDNA platform developed by Sulc group and their colleagues (https ://github.com/lorenzo-rovigatti/oxDNA). |

For manuscripts utilizing custom algorithms or software that are central to the research but not yet described in published literature, software must be made available to editors and reviewers. We strongly encourage code deposition in a community repository (e.g. GitHub). See the Nature Portfolio guidelines for submitting code & software for further information.

## Data

Policy information about availability of data

All manuscripts must include a data availability statement. This statement should provide the following information, where applicable:

- Accession codes, unique identifiers, or web links for publicly available datasets
- A description of any restrictions on data availability
- For clinical datasets or third party data, please ensure that the statement adheres to our policy

The source data in this study have been deposited in the repository https://doi.org/10.5281/zenodo.17362995.

## Research involving human participants, their data, or biological material

Policy information about studies with human participants or human data. See also policy information about sex, gender (identity/presentation), and sexual orientation and race, ethnicity and racism.

| | |
|---|---|
| Reporting on sex and gender | N/A |
| Reporting on race, ethnicity, or other socially relevant groupings | N/A |
| Population characteristics | N/A |
| Recruitment | N/A |
| Ethics oversight | N/A |

Note that full information on the approval of the study protocol must also be provided in the manuscript.

# Field-specific reporting

Please select the one below that is the best fit for your research. If you are not sure, read the appropriate sections before making your selection.

☒ Life sciences ☐ Behavioural & social sciences ☐ Ecological, evolutionary & environmental sciences

For a reference copy of the document with all sections, see nature.com/documents/nr-reporting-summary-flat.pdf

# Life sciences study design

All studies must disclose on these points even when the disclosure is negative.

| | |
|---|---|
| Sample size | Sample sizes for incorporation efficiency study were determined based on the number of non-aggregating DNA origami in a 256 pixel by 256 pixel pixel of DNA-PAINT super-resolution images where 1 pixel is 117 nm in size. For the clustering and alignment data processing for decryption study, the sample size was predetermined to be at least 100 and at most 300 based on the technical feasibility such as time, data storage and computational resources. Visual inspection was involved to pick the molecules from ROI to ensure non-aggregating molecules were picked instead of aggregating ones.<br><br>The exact sample sizes are:<br>Fig. 2: Panels C, D, E from top to bottom: N = 3500,5993,1793,765 molecules.<br>Fig. 3: N = 108 for N letter, 114 for S letter,and 116 for F letter. All picked molecules are also presented in the supporting information figures.<br>Fig. 4: N = 200 molecules for each data set. All picked molecules are also presented in the supporting information figures.<br>Fig. 5: N = 161, 107 for , 108, and 168 (Panel C, left to right). All picked molecules are also presented in the supporting information figures.<br>Fig. 6: N = 173 molecules.<br>Fig. 7: N = 26, 28, 14, and 14 (panel G, left to right). All picked molecules are also presented in the supporting information figures. |
| Data exclusions | Aggregating particles of DNA origami in DNA-PAINT super-resolution images were excluded in our picked molecules. |
| Replication | All experiments were repeated at least three times independently. All replications were successful. For the decryption process, the replicates were from the data analysis of running clustering, template alignment and pattern matching to obtain the standard deviation of the readout. |
| Randomization | We randomly picked DNA origami molecules from an ROI. The only criteria that we used was excluding the aggregating particles in our picks. |
| Blinding | Blinding is not relevant or necessary for this study. The research involves nanostructure fabrication, imaging, and data analysis, not subjective measurement or treatment assignment. Reliability is ensured through algorithmic validation and experimental controls, not through participant or investigator blinding |

# Reporting for specific materials, systems and methods

We require information from authors about some types of materials, experimental systems and methods used in many studies. Here, indicate whether each material, system or method listed is relevant to your study. If you are not sure if a list item applies to your research, read the appropriate section before selecting a response.

## Materials & experimental systems

| n/a | Involved in the study |
| --- | --- |
| ☒ | ☐ Antibodies |
| ☒ | ☐ Eukaryotic cell lines |
| ☒ | ☐ Palaeontology and archaeology |
| ☒ | ☐ Animals and other organisms |
| ☒ | ☐ Clinical data |
| ☒ | ☐ Dual use research of concern |
| ☒ | ☐ Plants |

## Methods

| n/a | Involved in the study |
| --- | --- |
| ☒ | ☐ ChIP-seq |
| ☒ | ☐ Flow cytometry |
| ☒ | ☐ MRI-based neuroimaging |

# Plants

| | |
| --- | --- |
| Seed stocks | Not relevant to the study. |
| Novel plant genotypes | Not relevant to the study. |
| Authentication | Not relevant to the study. |
