# Supplementary material for: High-speed 3D DNA PAINT and unsupervised clustering for unlocking 3D DNA origami cryptography
Source: Nat Commun. 2025 Dec 13;16:11514. doi: 10.1038/s41467-025-66338-y (PMC12749235; doi:10.1038/s41467-025-66338-y)
Supplement: Supplementary file 2 — Reporting Summary [file 41467_2025_66338_MOESM2_ESM.pdf]

nature portfolio

Corresponding author(s): Hariadi, Rizal
Wisna, Gde Bimananda Mahardika

Last updated by author(s): October 22, 2025

Reporting Summary

Nature Portfolio wishes to improve the reproducibility of the work that we publish. This form provides structure for consistency and transparency in reporting. For further information on Nature Portfolio policies, see our Editorial Policies and the Editorial Policy Checklist.

Statistics

For all statistical analyses, confirm that the following items are present in the figure legend, table legend, main text, or Methods section.

n/a	Confirmed	
☐	☒	The exact sample size (*n*) for each experimental group/condition, given as a discrete number and unit of measurement
☐	☒	A statement on whether measurements were taken from distinct samples or whether the same sample was measured repeatedly
☒	☐	The statistical test(s) used AND whether they are one- or two-sided<br>*Only common tests should be described solely by name; describe more complex techniques in the Methods section.*
☒	☐	A description of all covariates tested
☒	☐	A description of any assumptions or corrections, such as tests of normality and adjustment for multiple comparisons
☐	☒	A full description of the statistical parameters including central tendency (e.g. means) or other basic estimates (e.g. regression coefficient) AND variation (e.g. standard deviation) or associated estimates of uncertainty (e.g. confidence intervals)
☒	☐	For null hypothesis testing, the test statistic (e.g. *F*, *t*, *r*) with confidence intervals, effect sizes, degrees of freedom and *P* value noted<br>*Give P values as exact values whenever suitable.*
☒	☐	For Bayesian analysis, information on the choice of priors and Markov chain Monte Carlo settings
☒	☐	For hierarchical and complex designs, identification of the appropriate level for tests and full reporting of outcomes
☒	☐	Estimates of effect sizes (e.g. Cohen's *d*, Pearson's *r*), indicating how they were calculated

Our web collection on statistics for biologists contains articles on many of the points above.

Software and code

Policy information about availability of computer code

Data collection	A Benchtop Nanoimager S Mark II with total internal reflection fluorescence (TIRF) setup, with NIMos software was used for operating the microscope and acquiring the data.(https://oni.bio/nanoimager/software/nimos-software/). A Bruker multimode AFM was used for imaging DNA origami.
Data analysis	DNA-PAINT movies were processed using open source software FIJI to select specific region of interest (ROI) and Picasso to localize the point spread function, to render the results and to pick the DNA-PAINT molecules which is developed by Jungmann Group (https://github.com/jugmannlab/picasso). The Incorporation efficiency analysis was done using a custom Matlab script. 2D and 3D data clustering, template alignment, and analysis were performed with a custom Python and Matlab. The custom codes are available on: (https://github.com/Jonathanzhao02/smlm_classification2d) and (https://github.com/gwisna/DNA-origami-cryptography-code-and-data)<br>The oxDNA simulation and 3D alignment of the unrelaxed and mean structure with experimental DNA-PAINT data were carried out using open source software oxDNA platform developed by Sulc group and their colleagues (https ://github.com/lorenzo-rovigatti/oxDNA).

For manuscripts utilizing custom algorithms or software that are central to the research but not yet described in published literature, software must be made available to editors and reviewers. We strongly encourage code deposition in a community repository (e.g. GitHub). See the Nature Portfolio guidelines for submitting code & software for further information.

Data

Policy information about availability of data

All manuscripts must include a data availability statement. This statement should provide the following information, where applicable:

- Accession codes, unique identifiers, or web links for publicly available datasets
- A description of any restrictions on data availability
- For clinical datasets or third party data, please ensure that the statement adheres to our policy

The source data in this study have been deposited in the repository https://doi.org/10.5281/zenodo.17362995.

Research involving human participants, their data, or biological material

Policy information about studies with human participants or human data. See also policy information about sex, gender (identity/presentation), and sexual orientation and race, ethnicity and racism.

Reporting on sex and gender	N/A
Reporting on race, ethnicity, or other socially relevant groupings	N/A
Population characteristics	N/A
Recruitment	N/A
Ethics oversight	N/A

Note that full information on the approval of the study protocol must also be provided in the manuscript.

Field-specific reporting

Please select the one below that is the best fit for your research. If you are not sure, read the appropriate sections before making your selection.

☒ Life sciences ☐ Behavioural & social sciences ☐ Ecological, evolutionary & environmental sciences

For a reference copy of the document with all sections, see nature.com/documents/nr-reporting-summary-flat.pdf

Life sciences study design

All studies must disclose on these points even when the disclosure is negative.

Sample size	Sample sizes for incorporation efficiency study were determined based on the number of non-aggregating DNA origami in a 256 pixel by 256 pixel pixel of DNA-PAINT super-resolution images where 1 pixel is 117 nm in size. For the clustering and alignment data processing for decryption study, the sample size was predetermined to be at least 100 and at most 300 based on the technical feasibility such as time, data storage and computational resources. Visual inspection was involved to pick the molecules from ROI to ensure non-aggregating molecules were picked instead of aggregating ones.<br><br>The exact sample sizes are:<br>Fig. 2: Panels C, D, E from top to bottom: N = 3500,5993,1793,765 molecules.<br>Fig. 3: N = 108 for N letter, 114 for S letter,and 116 for F letter. All picked molecules are also presented in the supporting information figures.<br>Fig. 4: N = 200 molecules for each data set. All picked molecules are also presented in the supporting information figures.<br>Fig. 5: N = 161, 107 for , 108, and 168 (Panel C, left to right). All picked molecules are also presented in the supporting information figures.<br>Fig. 6: N = 173 molecules.<br>Fig. 7: N = 26, 28, 14, and 14 (panel G, left to right). All picked molecules are also presented in the supporting information figures.
Data exclusions	Aggregating particles of DNA origami in DNA-PAINT super-resolution images were excluded in our picked molecules.
Replication	All experiments were repeated at least three times independently. All replications were successful. For the decryption process, the replicates were from the data analysis of running clustering, template alignment and pattern matching to obtain the standard deviation of the readout.
Randomization	We randomly picked DNA origami molecules from an ROI. The only criteria that we used was excluding the aggregating particles in our picks.
Blinding	Blinding is not relevant or necessary for this study. The research involves nanostructure fabrication, imaging, and data analysis, not subjective measurement or treatment assignment. Reliability is ensured through algorithmic validation and experimental controls, not through participant or investigator blinding

Reporting for specific materials, systems and methods

We require information from authors about some types of materials, experimental systems and methods used in many studies. Here, indicate whether each material, system or method listed is relevant to your study. If you are not sure if a list item applies to your research, read the appropriate section before selecting a response.

Materials & experimental systems

n/a	Involved in the study
☒	☐ Antibodies
☒	☐ Eukaryotic cell lines
☒	☐ Palaeontology and archaeology
☒	☐ Animals and other organisms
☒	☐ Clinical data
☒	☐ Dual use research of concern
☒	☐ Plants

Methods

n/a	Involved in the study
☒	☐ ChIP-seq
☒	☐ Flow cytometry
☒	☐ MRI-based neuroimaging

Plants

Seed stocks	Not relevant to the study.
Novel plant genotypes	Not relevant to the study.
Authentication	Not relevant to the study.
